# Comprehensive quantitative modeling of translation efficiency in a genome‐reduced bacterium

**DOI:** 10.15252/msb.202211301

**Published:** 2023-08-29

**Authors:** Marc Weber, Adrià Sogues, Eva Yus, Raul Burgos, Carolina Gallo, Sira Martínez, Maria Lluch‐Senar, Luis Serrano

**Affiliations:** ^1^ Centre for Genomic Regulation (CRG) The Barcelona Institute of Science and Technology Barcelona Spain; ^2^ Universitat Pompeu Fabra (UPF) Barcelona Spain; ^3^ ICREA Barcelona Spain

**Keywords:** data integration, *Mycoplasma pneumoniae*, protein synthesis, ribosome profiling, translation efficiency, Microbiology, Virology & Host Pathogen Interaction, Translation & Protein Quality

## Abstract

Translation efficiency has been mainly studied by ribosome profiling, which only provides an incomplete picture of translation kinetics. Here, we integrated the absolute quantifications of tRNAs, mRNAs, RNA half‐lives, proteins, and protein half‐lives with ribosome densities and derived the initiation and elongation rates for 475 genes (67% of all genes), 73 with high precision, in the bacterium *Mycoplasma pneumoniae* (*Mpn*). We found that, although the initiation rate varied over 160‐fold among genes, most of the known factors had little impact on translation efficiency. Local codon elongation rates could not be fully explained by the adaptation to tRNA abundances, which varied over 100‐fold among tRNA isoacceptors. We provide a comprehensive quantitative view of translation efficiency, which suggests the existence of unidentified mechanisms of translational regulation in *Mpn*.

## Graphical abstract

Integration of the absolute quantifications of mRNAs, RNA half‐lives, proteins, protein half‐lives, tRNAs, with ribosome densities offers a comprehensive quantitative study of translation efficiency in the genome‐reduced bacterium *Mycoplasma pneumoniae* (*Mpn*).

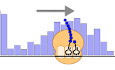

## Introduction

Quantitative analysis of gene expression mainly relies on the measurement of mRNA levels by means of high‐throughput sequencing methods. Nonetheless, mRNA level is only an approximate proxy for protein abundance, with a coefficient of determination in the range of ∼0.4–0.6 (Schwanhäusser *et al*, 2011; Wilhelm *et al*, 2014), suggesting that protein synthesis and decay rates are important layers of gene expression regulation. Translational control has been shown to play an important role in shaping the cellular proteome in bacteria (Li *et al*, 2014; Samatova *et al*, 2020; Tollerson & Ibba, 2020; Fan *et al*, 2021), eukaryotes (Leppek *et al*, 2018), human cells (Gingold *et al*, 2014; Jang *et al*, 2015), cancer cells (Gonzalez *et al*, 2014; Truitt & Ruggero, 2016; Robichaud *et al*, 2019), and virus‐infected cells (Stern‐Ginossar *et al*, 2019; Hernandez‐Alias *et al*, 2021). In *Escherichia coli*, proteins that are members of multiprotein complexes are produced in precise proportion to their stoichiometry mostly by tuning translation rates (Li *et al*, 2014).

In principle, translation can be regulated at any of its essential phases, which include initiation, elongation, and termination. As initiation is in general the slowest phase of translation, it is usually assumed to control the frequency at which a mRNA is translated into a protein (Shah *et al*, 2013; Cambray *et al*, 2018; Riba *et al*, 2019), i.e. the *translation efficiency* (TE). The translation initiation rate can be controlled by various mechanisms. In many bacteria, the ribosome interacts with specific nucleotide sequence motifs near the 5′ end of the mRNA called Shine‐Dalgarno (SD) motifs, which promotes the assembly of the translation initiation complex and helps the recognition of the correct translation start codon on the mRNA (Kozak, 1999). The strength of this interaction, depending on the degree of complementation between the SD motif and the anti‐SD (aSD) sequence found in the 16S rRNA of the ribosomal small unit, can modulate more than 200‐fold the translation initiation rate (Levin‐Karp *et al*, 2013; Ding *et al*, 2020). In a similar manner, the presence of A/U‐rich sequences can be recognized by the ribosomal protein S1 in *E. coli*, which may favor ribosome binding (Boni *et al*, 1991; Komarova *et al*, 2005; Cifuentes‐Goches *et al*, 2019). Secondary structure in the region close to the start codon can also influence the initiation rate by hindering or facilitating access of the ribosome to the translation initiation region (Chiaruttini & Guillier, 2020).

Translation elongation can also affect translation efficiency. Multiple factors have been identified that can influence elongation rates, including codon usage and codon arrangement along the coding sequence (Hanson & Coller, 2018), codon context (Gamble *et al*, 2016), adaptation to the tRNA pool (Gorochowski *et al*, 2015; Mohammad *et al*, 2019), specific amino acid sequences that induce translation stalling (Woolstenhulme *et al*, 2013), positively charged amino acids in the nascent peptide (Dao Duc & Song, 2018), and mRNA secondary structure along the coding sequence (Tholstrup *et al*, 2012; Gorochowski *et al*, 2015). In the absence of frameshifting, translation termination is generally assumed to occur at a rate that is faster than elongation and with high efficiency as observed in *E. coli* (Gorochowski *et al*, 2019).

The ribosome profiling method (Ingolia *et al*, 2009) has been central to gaining insight into the mechanisms of translation control. Analysis of ribosome profiling data using mathematical models has revealed determinants of local variation in ribosome densities and translation elongation rates (Ciandrini *et al*, 2013; Shah *et al*, 2013; Gritsenko *et al*, 2015; Dao Duc & Song, 2018; Szavits‐Nossan & Ciandrini, 2020). However, ribosome density alone is not sufficient to determine both the initiation and the elongation rates of a gene, as a global increase in ribosome density can be equally caused by an increase in the initiation rate or by a decrease in the average elongation rate along the transcript. Therefore, additional measurements of the rates of protein synthesis, turnover, and protein abundances are needed to estimate translation efficiency (Lahtvee *et al*, 2017; Riba *et al*, 2019).

Despite many advances in our understanding of the features that control translation efficiency, the existing models still fail to account for a large part of the differences in protein production. In *E. coli*, a recent study using a randomized library of mRNA sequences could explain ∼50% of the variance in protein abundance based on known determinants (Cambray *et al*, 2018). In yeast, known sequence features together with mRNA levels could explain ∼50–60% of the variance in translation efficiency (Weinberg *et al*, 2016; Riba *et al*, 2019). In addition, protein turnover, while rarely taken into account in quantitative studies, has been reported to play an important role in fine‐tuning protein levels (Pratt *et al*, 2002; Belle *et al*, 2006; Helbig *et al*, 2011). This suggests the existence of other unknown features influencing translation in prokaryotes and eukaryotes.

In this study, we aimed to achieve a quantitative understanding of translation efficiency in a genome‐reduced model bacterium, *Mycoplasma pneumoniae* (*Mpn*). With a reduced genome and a small set of tRNA genes, this organism is well‐suited to study the general mechanisms controlling translation. Previously we quantified its mRNA levels (Maier *et al*, 2011) and their half‐lives (Junier *et al*, 2016), the role that the identity of the C‐terminal residue has on protein abundance (Weber *et al*, 2020), as well as protein half‐lives (Burgos *et al*, 2020). Herein we measured genome‐wide ribosome densities by means of ribosome profiling, in standard growth condition. To quantify translation rates, we measured absolute protein abundances using a quantitative mass spectrometry approach for 75% of the genes (528 proteins). By integrating the absolute quantifications of mRNA abundances, protein abundances, and protein half‐lives with ribosome density, we derived initiation and elongation rates for 475 genes, 73 with high precision. We found that, although the initiation rate varied over 160‐fold among genes, most of the known factors had little impact on translation efficiency in *Mpn*. Rather, our data suggest a general trend of elongation rate adaptation to the initiation rate such as to maintain the ribosome density approximately constant. In order to understand local variations in translation rates along the transcripts, we derived codon elongation rates in absolute units from the ribosome density data. In addition, we measured cellular abundances of all tRNA species by means of hydro‐tRNA‐seq (Gogakos *et al*, 2017). We found that tRNA abundances varied over 100‐fold, but that these variations could not fully explain codon elongation rates. Moreover, we found evidence that internal SD‐like motifs slow down local elongation rate. The above results suggest as in other species the existence of unidentified mechanisms of translational regulation in *Mpn*. On the whole, we provide a comprehensive quantitative view of translation efficiency in a genome‐reduced organism.

## Results

### Ribosome profiling in *Mycoplasma pneumoniae*

The ribosome profiling protocol was adapted to the characteristics of *Mpn* (see Materials and Methods). As a control, we also performed ribosome profiling in *E. coli*. Micrococcal nuclease (MNase) treatment did not significantly change the polysome profile as compared to undigested control samples (see Materials and Methods, Appendix Fig S17A and B), suggesting that in *Mpn* most ribosomes are found assembled as 70S complexes and that the proportion of polysomes is low. Indeed, recent studies using cryo‐electron tomography in *Mpn* cells indicated that approximately 16–25% of the detected 70S ribosomes were in the closely assembled polysome configuration (O'Reilly *et al*, 2020; Xue *et al*, 2022).

**Figure 1:**
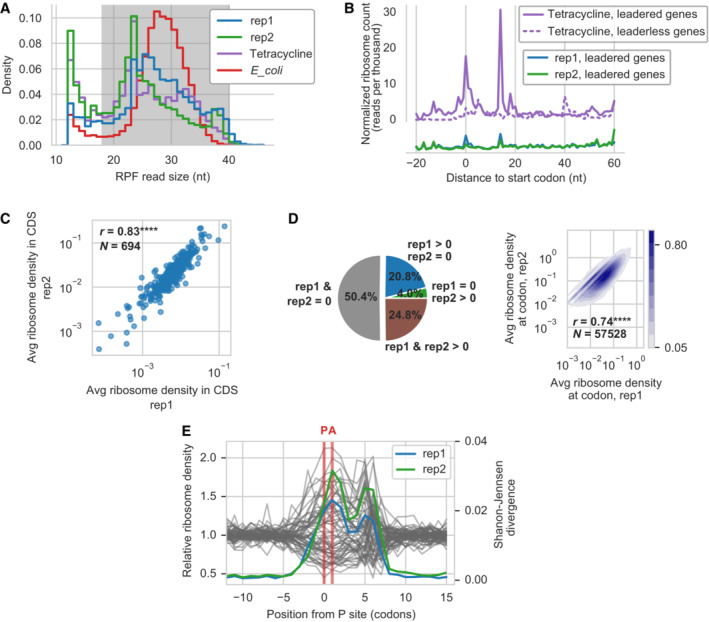
Quality control of ribosome profiling data in *Mpn* Ribosome protected footprints (RPFs) length distribution for samples: two replicates of *Mpn* in exponential growth phase condition at 6 h of growth (rep1, rep2), *Mpn* at 6 h of growth with tetracycline treatment (tetracycline) and *E. coli* sample (*E*_*coli*). Footprints of length between 18 and 40 nt (shaded area) were selected for downstream analyses.Metagene profile of normalized ribosome counts aligned to the first nucleotide of the start codon of CDSs. Tetracycline‐treated sample showed a peak of ribosomes at the start codon of leadered (length of 5′UTR > 8) genes, validating the calibration of the P‐site shift of footprints. The peak was absent for leaderless‐like (length of 5′UTR ≤ 8) genes. Samples treated with chloramphenicol in standard growth conditions (rep1, rep2) showed a similar peak with lower intensity.Reproducibility at the gene level. Correlation of the average ribosome density in CDSs for the two replicates rep1 and rep2. Pearson correlation coefficient in linear space *r* = 0. 83 (*N* = 695, *P* ≈ 0, two‐sided test).Reproducibility at the codon level. Proportion of codons in the transcriptome of *Mpn* with non‐null ribosome occupancy in the two standard growth replicates rep1 and rep2 (left). Correlation of ribosome density at codons between the two replicates (right), limited to the subset of codons with non‐null ribosome occupancy (24.8% of total codons). The color code indicates the density of data points. Pearson correlation coefficient in linear space *r* = 0. 74 (*N* = 57,528, *P* ≈ 0).Metafootprint analysis of relative ribosome density normalized by CDS average (gray lines), depending on the codon context (only sample rep1 is shown). The relative entropy in ribosome density depending on the codon identity at a specific position from the P‐site was quantified as the Jensen‐Shannon divergence (colored lines, samples rep1 and rep2). P‐site and A‐site positions are indicated by vertical red lines. Data information: *P*‐value legend: ****P < 10^−4^.

Ribosome profiling studies in *E. coli* and *Bacillus subtilis* have shown that bacterial ribosome protected footprints (RPFs) vary widely in length (Mohammad *et al*, 2019), in the range 15–40 nt, for reasons that remain unclear. RPFs in *Mpn* exhibited a broad range of lengths (see Fig 1A), with a majority of reads in the range 18–40 nt. The most frequent length for the standard growth condition in *Mpn* was 24 nt (average between the two replicates rep1 and rep2). The RPFs in the *E. coli* sample showed a most frequent length of 27 nt, slightly longer than the typical footprint length reported in previous studies (Mohammad *et al*, 2019). We also observed a large number of RPFs of 12 nt length in all samples, the minimum read length allowed in the processing pipeline. Relaxing the minimum mapping quality threshold, inversely related to the uniqueness of the read alignment, increased the number of reads of lengths close to 12 nt (Appendix Fig S1), which suggests that at least part of those reads originated from incorrect alignment to the genome due to their small size.

The calibration of the P‐site position relative to the footprint fragment is crucial for obtaining reliable ribosome density at the codon resolution level. Tetracycline has been shown to produce a sharp accumulation of footprints at the location where the initiation codon is at the P‐site of the ribosome, corresponding to the translation initiation complex (Nakahigashi *et al*, 2016). Thus, in order to calibrate the P‐site shifts, we used a tetracycline‐treated sample and computed the accumulated coverage of the 5′ or 3′ ends of the footprints when aligning all CDSs to the start codon (see Materials and Methods, Dataset EV1). Given the large variability of footprint lengths and the need to gather sufficient statistics, we selected a broad range of footprint sizes, from 18 to 40 nt (both included) (Fig 1A), and used our calibrated P‐site shifts to produce ribosome occupancy profiles at the single nucleotide resolution. Hereinafter, we refer to the ribosome occupancy position as the first nucleotide of the ribosome P‐site. The metagene profile of ribosome occupancy (Fig 1B) reflected the observed primary peak at the start codon, and also a secondary peak at position 14 nt, which remained unexplained (see Discussion). This secondary peak corresponds to an accumulation of ribosomes with their P‐site on the 5th codon. The primary and the secondary peaks could not be detected in the metagene profile of leaderless‐like transcripts, which were defined here as mRNAs with a 5′UTR length of 8 nt or smaller (50 cases), following the rationale that such a short length would exclude the presence of a ribosome binding site (Osterman *et al*, 2013). Metagene profiles for the chloramphenicol treated samples in exponential phase (rep1 and rep2) showed a similar albeit smaller peak at the same position from the start codon (Fig 1B), supporting the calibration.

In the downstream analyses, ribosome occupancy was summed at the three sub‐codon positions to yield a ribosome occupancy profile at the codon level. Ribosome occupancy was further divided by the mRNA abundance, as measured by RNA‐seq, to obtain ribosome density.

We assessed the reproducibility of ribosome count and ribosome density by comparing the two biological replicates of *Mpn* cultured in standard growth conditions. The total ribosome counts per gene showed excellent reproducibility between the two replicates (Pearson *r* = 0.99, *N* = 695, *P* ≈ 0, two‐sided test) (Appendix Fig S2A–C). As expected, the average ribosome density at the gene level (Fig 1C) showed a slightly lower correlation (*r* = 0.83, *N* = 694, *P* ≈ 0), due to the combined noise of mRNA and ribosome footprints measurements.

At the codon level, ribosome occupancy was sparse (Fig 1D), with 50.4% of codons in the transcriptome of *Mpn* not showing any ribosome count in any of the replicates, while only 24.8% of codons had non‐zero ribosome count in both replicates. Despite this sparsity in ribosome coverage, the correlation at codon positions with non‐zero ribosome counts was moderately high (*r* = 0.74, *N* = 57,528, *P* ≈ 0).

To evaluate the general influence of codon identity and mRNA sequence on the ribosome density, and validate the calibration of the P‐site, we followed an approach similar to Artieri and Fraser (2014), and O'Connor *et al* (2016) (see Materials and Methods and Fig 1E). The resulting metafootprint profile revealed that the highest divergence in the distribution of average ribosome densities (lowest entropy) was found for the A‐site, suggesting that the codon identity at this position had the strongest impact on elongation speed. This result also supports the calibration of the P‐site shift determined above. The positions of positive divergence were contained within an area ranging from position −4 to position +8 codons. A second peak in divergence could be observed at position +5, for the codon located 15–17 nt from the P‐site. This position is close to the 3′ of footprints, suggesting that this contribution might be due to sequence biases in library generation and MNase cleavage site (O'Connor *et al*, 2016). The same sequence biases of the MNase could be responsible for the lack of 3 nt periodicity observed in the metagene profile, as was suggested for ribosome profiling data in *E. coli* (Hwang & Buskirk, 2017) (Fig 1B, see Materials and Methods). Thus, our results suggest that the codon identities in a window of 12 codons have a significant influence on ribosome density. This result is in agreement with previous studies of ribosome profiling data in yeast (Tunney *et al*, 2018), where the local sequence features in the window −4 to +5 codons were found to best predict ribosome density.

### Absolute protein and mRNA quantification

To quantify the absolute rate of translation of proteins, we measured the absolute levels of individual transcripts and proteins in *Mpn*.

For the quantification of proteins, a set of 77 labeled peptides were synthesized and used as internal standard peptides to measure the absolute concentrations of 73 reference proteins in a tandem mass spectrometer (Gerber *et al*, 2003) (Datasets EV2 and EV3, see Materials and Methods). A calibration curve between the absolute amount of reference proteins and the proteome‐wide protein intensities measured by label‐free mass spectrometry allowed the estimation of absolute protein abundances for all detected proteins (Schmidt *et al*, 2016) (Dataset EV4). In order to further improve the quantification, four additional label‐free mass spectrometry datasets of *Mpn* (Miravet‐Verde *et al*, 2019) using two different protein extraction methods (SDS and urea) were combined with the original dataset, which allowed to quantify the abundances of 528 proteins (75% of the proteome) (see Materials and Methods). The total number of proteins per cell obtained ranged between 1.15 × 10^5^ and 1.54 × 10^5^ depending on the sample, with an average over all samples of 1.207 × 10^5^ copies per cell.

The absolute numbers of mRNA molecules were estimated from the normalized read counts for six RNA‐seq datasets of *Mpn* in exponential growth (Dataset EV5) (Junier *et al*, 2016). The number of assembled ribosome complexes has been estimated to be between 300 and 500 by cryo‐electron tomography (Seybert *et al*, 2006; Kühner *et al*, 2009; Maier *et al*, 2011; O'Reilly *et al*, 2020). This number agreed well with the abundances of the ribosomal proteins determined by our absolute quantification based on label‐free mass spectrometry (median 300 copies per cell). By using the normalized read counts of the 23S rRNA as a reference, and assuming that 90% of the rRNA is bound to the ribosome, we computed copy numbers per cell for all cellular mRNAs. This method of calibration depended only on the estimation of the number of ribosomes per cell, determined by two independent experimental methods: cryo‐EM and mass spectrometry. The conversion resulted in a total number of 46.5 mRNA molecules (average for the 6 samples), assuming one mRNA species per gene (disregarding polycistronic mRNAs). Due to its small volume (0.075 μm^3^ compared to 1.5–4.4 μm^3^ for *E. coli*; Volkmer & Heinemann, 2011) and relatively slow transcription rates (average of 0.47 h^−1^, compared to 3–300 h^−1^ in *E. coli*; Chen *et al*, 2015; Iyer *et al*, 2016), the average copy number of each mRNA molecule in *Mpn* is well below unity (0.066 copies), and the total mRNA concentration approximately three times smaller compared to *E. coli* (Bartholomäus *et al*, 2016; Gorochowski *et al*, 2019). Given the absolute mRNA copy number of each gene, we calculated a total amount of 1.400 × 10^4^ codons present in the cell at a given time. As the vast majority of the 70S ribosome complexes identified in cryo‐EM studies in *Mpn* were found in an active elongation phase (Xue *et al*, 2022), we estimated a number of 240 copies of ribosomes actively translating. Based on this estimate, a ribosome will be found, on average, every 58 codons along the coding sequences. We used this average value to convert the relative ribosome profiling data to ribosome densities in absolute units. Importantly, the relatively low ribosome density, similar to the density observed in yeast (Riba *et al*, 2019) (∼0.02 ribosome codon^−1^), suggests that ribosome collisions are infrequent for most of the genes in *Mpn*.

### Translation efficiency derived from absolute quantifications

**Figure 2:**
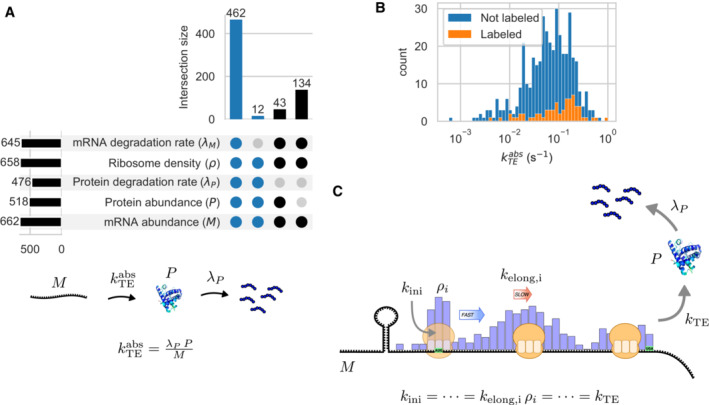
Modeling approach: integration of omics data and definition of translation efficiency Coverage in terms of the number of genes of the absolute quantifications: mRNA levels (by RNA‐seq), protein abundances (by mass spectrometry), protein degradation rates (by SILAC mass spectrometry), RNA half‐lives (by time‐course RNA‐seq), as well as ribosome density (by ribosome profiling). The number of genes for each of the experimental variables and the size of their intersections are shown in an upset plot. Subsets of size smaller than 10 were not shown. At steady‐state, the absolute translation efficiency ($k_{\mathrm{TE}}^{\mathrm{abs}}$), can be expressed as a function of the three absolute quantifications: number of molecules of mRNA (*M*), number of molecules of protein (*P*), and degradation rate of protein (*λ*_*P*_). Gene subsets for which $k_{\mathrm{TE}}^{\mathrm{abs}}$ could be calculated are shown in blue.Distribution of absolute translation efficiency $k_{\mathrm{TE}}^{\mathrm{abs}}$ for proteins that were quantified by labeled peptides (labeled) or label‐free mass spectrometry (not labeled).Schema of the initiation‐limited translation model: translation initiation rate (*k*_*ini*_), local translation elongation rate (*k*_*elong,i*_), local ribosome density (*ρ*_*i*_, blue bars) and translation efficiency (*k*_*TE*_). The assumptions of conservation of ribosome flow along the mRNA and the absence of ribosome queuing led to a simple relationship between local ribosome density, local elongation rate, and translation efficiency.

Translation efficiency can be determined in absolute units from the absolute quantification of mRNA levels, protein abundances, and protein degradation rates (Lahtvee *et al*, 2017) (Fig 2A). Protein half‐lives were previously measured by means of SILAC pulse‐chase method for *Mpn* cells in exponential phase (Burgos *et al*, 2020).

We selected 475 genes for which the three quantities (mRNA levels, protein abundances, and protein degradation rates) could be measured, representing 67% of the total number of genes and 87% of the genes for which we could detect at least one unique peptide in 116 mass spectrometry experiments (Miravet‐Verde *et al*, 2019). We computed, for these genes, the omics‐derived absolute translation efficiency $k_{\mathrm{TE}}^{\mathrm{abs}}$ (Fig 2A; Dataset EV6). The values of the absolute translation efficiency varied over 160‐fold (1–99 percentile) among genes, with a median value of 0.072 s^−1^ (Fig 2B). This value agreed well with translation initiation rates measured in *E. coli* (median 0.180 s^−1^) (Gorochowski *et al*, 2019), which under initiation‐limiting conditions are equivalent to translation efficiencies, and to absolute translation efficiencies measured in yeast (median 0.057 s^−1^) (Lahtvee *et al*, 2017).

As the expression of $k_{\mathrm{TE}}^{\mathrm{abs}}$ depended on three quantities, its relative error could be estimated as the sum of the relative errors of each measurement. The variability in the RNA copy number was relatively low (average coefficient of variation across replicates, CV_M_ = 0.16), similarly for the variability in protein degradation rate (CV_dP_ = 0.11). The abundances of proteins quantified by labeled peptides showed a relatively small variability (CV_P,labeled_ = 0.13). In contrast, the protein abundances estimated from the label‐free protein intensities exhibited as expected (Steen & Pandey, 2002) higher variability (CV_P,label‐free_ = 0.34) (see Materials and Methods). In this case, the real error in protein quantification was probably larger than the observed variability. Therefore, the average final coefficient of variation of $k_{\mathrm{TE}}^{\mathrm{abs}}$ for proteins with a labeled peptide was ${\mathrm{CV}}_{k_{\mathrm{TE}}^{\mathrm{abs}}}$ = 0.4, and ${\mathrm{CV}}_{k_{\mathrm{TE}}^{\mathrm{abs}}}$ > 0.61 for label‐free proteins. Indeed, the range of $k_{\mathrm{TE}}^{\mathrm{abs}}$ values was smaller, 129‐fold (1–99 percentile), for labeled proteins (Fig 2B).

### Determinants of translation initiation rates

To interpret ribosome profiling data, we used a simple model of translation in which we assumed that initiation is the rate‐limiting step, as suggested by the low average density of ribosomes on mRNAs in *Mpn* (Fig 2C and Materials and Methods). Although this assumption is under debate, several studies have suggested that it is valid for most of the genes in yeast (Shah *et al*, 2013; Riba *et al*, 2019) and *E. coli* (Cambray *et al*, 2018).

We first investigated which features control the initiation rate. We found that $k_{\mathrm{TE}}^{\mathrm{abs}}$ was weakly negatively correlated to the strength of the secondary structure in the region of the start codon (Pearson *r* = −0.24, *N* = 420, *P* = 1.1 × 10^−6^) (Appendix Fig S3A). This result is in agreement with a previous study using a library of randomized 5′UTR (Yus *et al*, 2017), where the presence of local hairpin structures close to the start codon decreased the expression of a reporter gene.

The strength of the RBS, based on the hybridization energy with the anti‐Shine‐Dalgarno (aSD) sequence, had no impact on translation efficiency (Appendix Fig S3B), in agreement with previous results (Yus *et al*, 2017). This is also consistent with the relatively low usage of SD sequences in *Mpn* (Ma *et al*, 2002; Nakagawa *et al*, 2010), where only 8.1% (55 out of 676 genes) of the genes harbor a SD motif and those are mostly found inside an operon. No differences were found in translation efficiency between leadered and leaderless‐like genes (Appendix Fig S4A).

Recent studies have shown that A‐rich sequences upstream of start codons promote initiation and play a role in the selection of the correct canonical start codon (Baez *et al*, 2019; Saito *et al*, 2020). We did not find any significant correlation (Pearson coefficient |*r*| < 0.2, *P* > 0.01) between the absolute translation efficiency and nucleotide composition in a window of 15 nt upstream of the start codon (Appendix Figs S4B and S5).

Similarly, the first codons at the N‐terminal of the coding sequence have been proposed to influence early elongation rates (Plotkin & Kudla, 2011; Verma *et al*, 2019). Because the elongation rate in the 5′ region of the CDS could become the limiting step in translation and effectively control the gene‐level translation efficiency, features in this region were analyzed against the absolute translation efficiency, $k_{\mathrm{TE}}^{\mathrm{abs}}$. We examined the 5′ amino acid composition at the first 15 residues and did not find any significant differences in $k_{\mathrm{TE}}^{\mathrm{abs}}$ (Appendix Fig S6). The same was true for the tRNA Adaptation Index (tAI) of the first 30 codons (Appendix Fig S3C) (Pearson coefficient *r* = 0.12, *N* = 474, *P* = 0.01). Moreover, the biophysical interactions between positively charged residues and the ribosome exit tunnel have been suggested to favor elongation of the nascent peptide at the N‐terminal (Dao Duc & Song, 2018). We found that the fraction of positively charged amino acids (Lys, Arg, His) in the first 35 residues at the N‐terminal did not correlate with $k_{\mathrm{TE}}^{\mathrm{abs}}$ (*r* = −0.04, *N* = 474, *P* = 0.3); however, the fraction of hydrophobic residues (Ala, Ile, Leu, Met, Val, Phe, Trp, Tyr) showed a weak negative correlation (*r* = −0.16, *N* = 474, *P* = 3.5 × 10^−4^) and the fraction of negatively charged residues (Glu, Asp) a weak positive correlation (*r* = 0.21, *N* = 474, *P* = 4.4 × 10^−6^) with the translation efficiency (Appendix Fig S7A–F). This suggests that hydrophobic residues at the N‐terminal could slow down the rate of translation initiation, while negative residues could have the opposite effect. Finally, no correlation was found between $k_{\mathrm{TE}}^{\mathrm{abs}}$ and RNA degradation rate (Appendix Fig S4C) (*r* = 0.08, *N* = 462, *P* = 0.08).

As a summary, we found that the translation initiation rate, or translation efficiency, was weakly negatively correlated to the strength of the secondary structure in the initiation region and to the fraction of hydrophobic residue at the N‐terminal, while it was weakly positively correlated to the fraction of negatively charged residues at the N‐terminal. Other features known to control translation initiation in other bacteria, such as the strength or presence of the SD motif, did not show any significant correlation.

### Elongation rates

**Figure 3:**
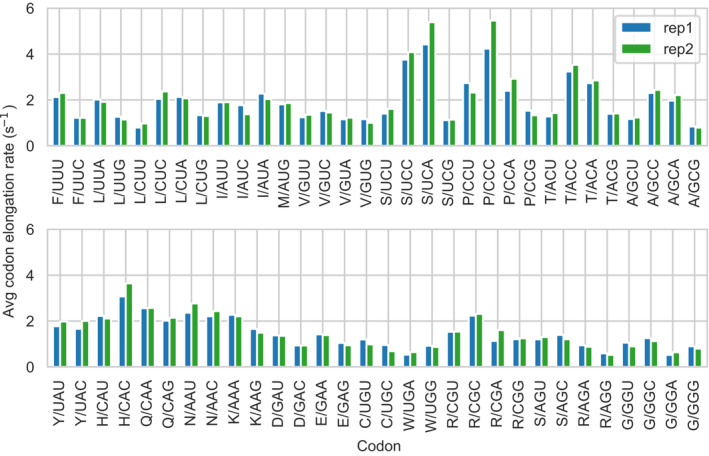
Estimation of average codon elongation rates Average codon elongation rates in absolute units, computed from the integration of ribosome densities and absolute translation efficiencies.

Following our modeling approach, we estimated average codon elongation rates in absolute units by averaging the local elongation rates *k*_*elong,i*_ over all identical codon positions in the genome (see Materials and Methods). We found that average codon elongation rates, *k*_*codon*_, varied 9‐fold (average between the two replicates), from 0.55 to 4.91, with a median of 1.46 codon·s^−1^ (Fig 3). The rates obtained showed a high reproducibility between the two replicates (Pearson correlation *r* = 0.98). These rates are approximately ∼5–10 times slower compared to the average elongation rate measured in *E. coli* (8–18 codon·s^−1^ depending on the growth condition) (Dai *et al*, 2016), 2 times slower compared to the inferred codon elongation rates in yeast (∼2–10 codons·s^−1^) (Dao Duc & Song, 2018) and 3 times slower than measured average elongation rate in mammalian cells (5.6 codons·s^−1^) (Ingolia *et al*, 2011). Between the species *Mpn*, *E. coli*, and yeast, the median elongation rate roughly scaled with the growth rate under good nutrient availability, as suggested by the approximately constant ratio *<k*_*codon*_> (s^−1^)/growth rate (s^−1^) = 6.1 × 10^4^ in *Mpn* for a doubling time of 8 h, 5.8 × 10^4^ in *E. coli* for a doubling time of 0.7 h (Dai *et al*, 2016), and 8.2 × 10^4^ in yeast for a doubling time of 100 min (Di Talia *et al*, 2007). This suggests that the slower translation elongation rate in *Mpn* could be related to its slow growth, probably due to a relaxed evolutionary pressure for higher protein synthesis rates.

**Figure 4:**
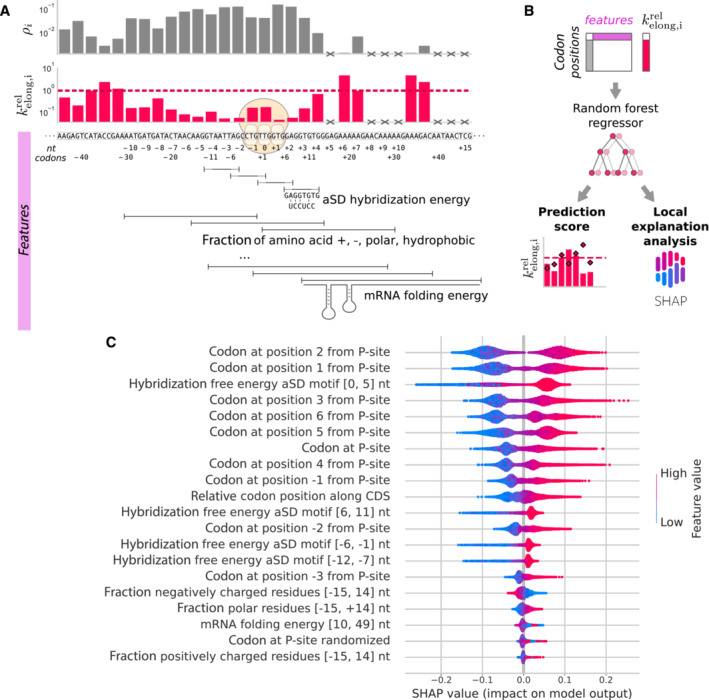
Determinants of the local variation in elongation rate The local variation in elongation rate $k_{\text{elong},i}^{\mathrm{rel}}$ was derived from the local ribosome density *ρ*_*i*_ and normalized by the average for each CDS. Codon positions with zero ribosome counts were excluded from the analysis (crosses). Multiple local features of the mRNA sequence were taken into account (features), such as codon identity or mRNA folding energy, and were computed in windows at upstream, centered, and downstream positions relative to the ribosome P‐site.A random forest regressor was trained on a subset of the codon positions to predict the local variation of elongation rate from local features of the mRNA sequence. Performance of the predictor was then evaluated on the test set, and the impact of input features on individual predictions was assessed with the TreeExplainer tool (local explanation analysis, SHAP values).Summary plot of the local explanation impact values in the random forest regressor. The SHAP values (horizontal axis) explain the impact on the model output, which is the local variation in elongation rate $k_{\text{elong},i}^{\mathrm{rel}}$, for each feature individually (vertical axis). Features were ordered by global relative importance. The distribution of local SHAP values for each codon position in the test set was shown as dots whose color represents the value of the feature (color code). For example, positions with more negative free energy of hybridization to the aSD sequence (blue dots), i.e. stronger SD‐like motifs, produced a negative impact on the model output (negative SHAP values), i.e. smaller local elongation rates. Codon identity features were encoded as ordinal numbers from 0 to 63 (color code) and ordered by the average A‐site codon elongation rates determined genome‐wide, such that slow codons (blue dots) had a negative impact on the local elongation rate, while fast codons had a positive impact (red dots).

At the local level, the velocity of elongation can be influenced by many features along the mRNA sequence. To see which factors could have an effect we analyzed the influence of local features of the mRNA sequence (Fig 4A) by computing their correlation to the local variation $k_{\text{elong},i}^{\mathrm{rel}}$ (see Materials and Methods and Appendix Fig S8). We included features such as the strength of internal SD‐like motifs (free energy of hybridization to the aSD sequence) (Li *et al*, 2014), the fraction of positively charged (Lys, Arg, His), negatively charged (Glu, Asp), polar (Asn, Gln, Ser, Thr), or hydrophobic residues (Ala, Ile, Leu, Met, Val, Phe, Trp, Tyr) in a 30 nt window (Dao Duc & Song, 2018), the presence of polyproline and polyglycine motifs (Artieri & Fraser, 2014), mRNA folding energy in a window of 40 nt, and the relative position along the CDS. Features were computed in windows at upstream, centered, and downstream positions relative to the ribosome P‐site (see Materials and Methods). Due to its higher coverage, only the first replicate was considered.

Then, to study the influence of sequence context in more detail, we built a random forest regressor to predict $k_{\text{elong},i}^{\mathrm{rel}}$ (Fig 4B) from the above features that we found to have a significant Pearson correlation (Appendix Fig S8), including as additional features the identity of the codons at positions −3 to +6. The regressor was only able to predict 9.6% of the variance of $k_{\text{elong},i}^{\mathrm{rel}}$ for the test set, suggesting that other unknown mechanisms control the elongation. The relative importance of the different features was assessed by using the SHAP TreeExplainer tool, a method based on Shapley values from game theory (Lundberg *et al*, 2020). The summary plot of the SHAP values (Fig 4C), which reflect the magnitude and direction of the impact on the model output for each feature individually, showed that the codon identity at positions +2 and +1 (A‐site) were the features with the highest impact on the local variation of the elongation rate.

The strength of SD‐like motifs in the window [0, 5] nt showed the third highest importance. Stronger SD‐like motifs (more negative free energy) had a negative impact on the predicted $k_{\text{elong},i}^{\mathrm{rel}}$. This result suggests that internal SD‐like motifs slow down the local rate of elongation and that the magnitude of their impact is similar to the one of the A‐site codon identity. The strength of SD‐like motifs in windows positioned farther away from the P‐site ([−12, −7], [−6, −1] and [6, 11] nt) had a weaker but significant influence as well.

The following six features in order of importance revealed that the codon identity in the vicinity of the A‐site, at positions −1 (E‐site), 0 (P‐site), +3, +4, +5, and +6, also had a moderate influence. The patterns of impact of the codon identity at all these positions were similar, which could be explained by the blurring of the ribosome density signal to the neighboring positions.

The relative codon position along the CDS had a positive impact, revealing a global trend of increasing elongation rate from the 5′ to the 3′ of the coding sequence. As the elongation rate is inversely proportional to the ribosome density, this effect revealed a trend of higher ribosome density at the 5′ of CDSs. At least two potential phenomena could produce this apparent trend. As previously described, samples treated with chloramphenicol show an artifactual accumulation of ribosomes at the 5′‐end of CDSs (Mohammad *et al*, 2019), because ribosomes continue initiating translation as the antibiotic inhibits elongation. In addition, ribosome drop‐off during elongation can also lead to a decrease in ribosome density toward the 3′ end of CDSs. In order to quantify these effects, we computed the normalized ribosome density profile averaged across all genes in bins of 33 codons. The profile showed a decrease in the first 400 codons, followed by a plateau (Appendix Fig S9A and B). We estimated a putative drop‐off rate of 3.07 × 10^−5^ codon^−1^ for rep1 and 3.66 × 10^−5^ codon^−1^ for rep2, 7.2‐fold lower compared to the average drop‐off rate observed in *E. coli* in normal growth condition (Sin *et al*, 2016). Thus, ribosome drop‐off and chloramphenicol bias probably have a limited impact on the distribution of ribosomes along mRNA sequences in *Mpn*.

The fraction of negatively charged residues in the window [−15, 14] nt had a small but consistent negative impact on $k_{\text{elong},i}^{\mathrm{rel}}$, while the fraction of polar residues in the same window had the opposite effect. The folding energy of the mRNA in the window [10, 49] nt had little global impact on $k_{\text{elong},i}^{\mathrm{rel}}$. However, a more detailed view of its local impact for all codon positions (Appendix Fig S10) revealed a U‐shaped impact function, where positions with a moderately strong structure (folding energy ∈ [−8, −3] kcal/mol) had a negative impact on the local elongation rate, while stronger structures (folding energy < −10 kcal/mol) had a positive impact. Finally, the randomized codon identity at the P‐site (negative control) showed one of the smallest importance, together with the fraction of positively charged residues, which had a negligible effect on the local elongation rate.

### tRNA levels

The relative abundances of the different tRNAs have long been hypothesized to influence translation elongation speed. In *E. coli*, the rates for aminoacyl‐tRNA binding to different codons span a 25‐fold range (Curran & Yarus, 1989), and preferred codons have been shown to be associated with more highly abundant tRNAs (Ikemura, 1981; Duret, 2000). tRNA repertoires are believed to have coevolved with codon usage to achieve a balance between supply and demand during translation (Bulmer, 1987; dos Reis *et al*, 2004; Yona *et al*, 2013). *Mpn*, characterized by a slow growing rate and a genome reduced along evolution, possesses 36 tRNA isoacceptors genes, all present at a single copy in the genome. Although this set of tRNA genes is small compared to fast‐growing bacteria (*E. coli*, ∼86 genes), *Mpn* has one of the largest numbers of tRNAs among Mollicutes (28–37 genes) (Grosjean *et al*, 2014). How tRNA levels may influence translation elongation in species with such a reduced set of tRNA genes, and how much tRNA abundances may vary, remains an open question.

**Figure 5:**
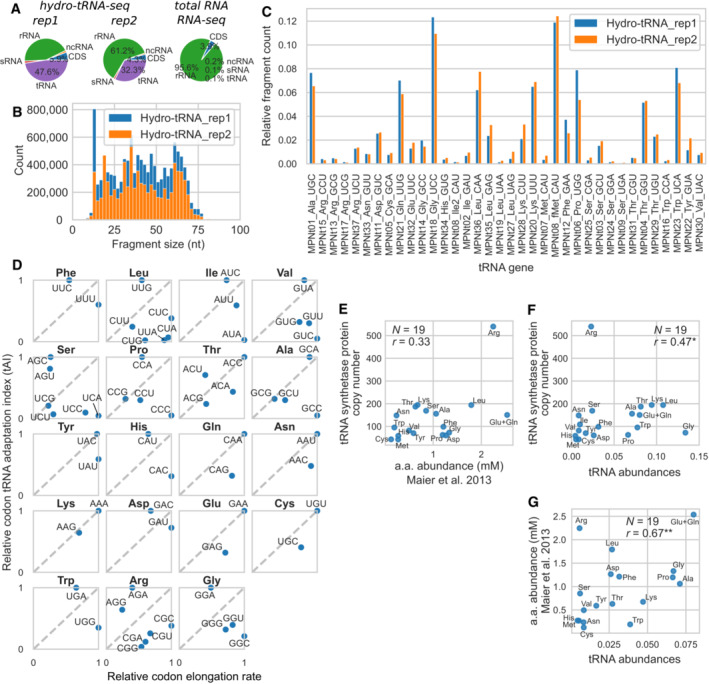
tRNA abundances measured by hydro‐tRNA‐seq and comparison to codon elongation rates, tRNA synthetases abundances and amino acid abundances Proportion of reads from the two replicates of the hydro‐tRNA‐seq experiment (left), or standard total RNA‐seq experiment (right), mapping to either tRNA genes, sRNAs, rRNA genes, ncRNAs, and CDSs.Fragment size distribution for hydro‐tRNA‐seq reads that mapped to tRNA genes.Relative fragment counts (normalized by total counts) of tRNA genes. The gene name indicates the anticodon and corresponding amino acid, and tRNA genes are ordered by the traditional order of the genetic code.Relative codon tRNA adaptation index, computed from the tRNA abundances, normalized within each synonymous codon family, versus relative codon elongation rates derived from the ribosome profiling data, also normalized within each family.Cytosolic abundances of amino acids determined by metabolomics (Maier *et al*, 2013) versus tRNA synthetases copy number. Pearson correlation coefficients (*r*) and associated two‐sided test *P*‐values in panels (E–G), were computed disregarding the outlier Arg.tRNA abundances (sum of isoacceptors) versus tRNA synthetases copy number.tRNA abundances (sum of isoacceptors) versus cytosolic abundances of amino acids. Data information: *P*‐value legend: *P < 0.05, **P < 10^−2^.

We measured abundances of tRNAs by means of hydro‐tRNA‐seq (Gogakos *et al*, 2017). The proportion of reads that mapped to the tRNA genes was very high (39.9%) compared to the same proportion in a normal RNA‐seq experiment (0.1%) (Fig 5A), confirming that the hydrolysis and size selection procedures successfully enriched the library for tRNA fragments. We selected reads that overlapped in their full length with a tRNA gene, in order to exclude fragments that could originate from immature tRNA transcripts, which would contain 5′ or 3′ flanking regions. The selected fragments showed a broad size distribution ranging from 12 to 80 nt (Fig 5B) (see Materials and Methods).

The relative tRNA abundances (Fig 5C, Dataset EV7), as measured by the relative fragment counts averaged over the two replicates, showed a moderate correlation with values measured by tiling array (Güell *et al*, 2009) (Pearson correlation *r* ≈ 0.25–0.35) and small RNA‐seq (Yus *et al*, 2012) (*r* ≈ 0.45). The tRNA abundances spanned a 139‐fold range, also showing large differences between tRNA isoacceptors. These differences were larger than the overall ∼25 fold differences in tRNA levels measured in *E. coli* by deep‐sequencing using a different library preparation method (Wang *et al*, 2021). For example, Thr‐tRNA‐GGU (MPNt04) was 10 times more abundant than Thr‐tRNA‐CGU (MPNt31).

These large differences raised the question of the role of tRNA abundances on codon decoding efficiencies, and thus elongation rates. We computed the tRNA Adaptation Index (tAI) (dos Reis *et al*, 2004) derived from the measured tRNA levels, by adapting the weights of the wobble base pairing to the characteristic set of tRNAs and codon decoding rules in *Mpn*. Overall, the tAI of codons showed no correlation with the codon elongation rates computed from the ribosome profiling data (Spearman rank correlation coefficient *r*_s_ = −0.16) (Appendix Fig S11A). This result suggests that the adaptation to the tRNA levels is not the main determinant of elongation speed in *Mpn*.

We then reasoned that the effect of the tRNA pool adaptation on elongation rates would be easier to isolate from other factors when examining the relative tAI among synonymous codons. We observed that in some synonymous families, the relative codon elongation rate followed the same pattern as the codon tAI (Fig 5D). In the Thr family and in the Arg 4‐codon box family, the two measures of elongation efficiency correlated well (Pearson coefficient *r* = 0.70, *N* = 4, *P* = 0.3, for Thr and *r* = 0.96, *N* = 4, *P* = 0.04 for Arg), but not for the other codon boxes. The small number of data points within each synonymous family limited the statistical significance of these comparisons.

We then asked whether tRNA abundances are adapted to the levels of amino acids and aminoacyl‐tRNA synthetases (aaRS) (see Materials and Methods). Arg appeared clearly as an outlier (Fig 5E). The arginyl‐tRNA‐synthetase, with 539 copies per cell, was much more abundant than any other aaRS (average of 109 copies). Even though Arg was the second most abundant amino acid, the four tRNA^Arg^s had relatively low abundances (percentiles 5, 20, 29, and 51) (Fig 5F). The cytosolic amino acid abundances, as previously measured by mass spectrometry (Maier *et al*, 2013), showed no significant correlation with the aaRSs copy number (Fig 5E, Dataset EV8) (Pearson *r* = 0.33, *N* = 19, *P* = 0.2, two‐sided test). When discarding Arg, we found a statistically significant correlation between the tRNA abundances and amino acids (*r* = 0.55, *N* = 19, *P* = 0.02) and aaRS levels (*r* = 0.47, *N* = 19, *P* = 0.05) (Fig 5F and G).

### Gene‐averaged elongation rates and consistency between translation efficiency and ribosome profiling data

**Figure 6:**
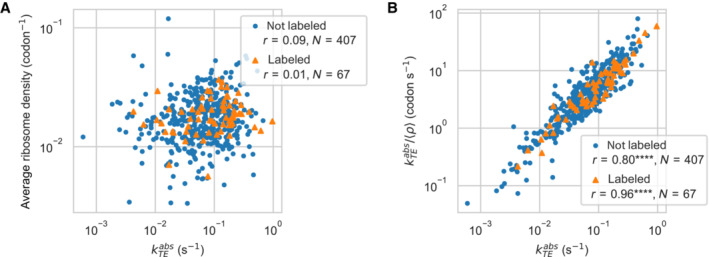
Consistency between translation efficiency and ribosome density Correlation between the average ribosome density at the gene level <*ρ*> and the absolute translation efficiency $k_{\mathrm{TE}}^{\mathrm{abs}}$. The precision of the translation efficiency was higher for proteins whose abundances were quantified using labeled peptides (labeled), compared to proteins quantified by label‐free mass spectrometry (not labeled). Pearson correlation (*r*) was calculated in the linear space.Correlation between the ratio of absolute translation efficiency to ribosome density, $k_{\mathrm{TE}}^{\mathrm{abs}}$/<*ρ*>, and the absolute translation efficiency $k_{\mathrm{TE}}^{\mathrm{abs}}$. In the initiation‐limited model of ribosome flow, the ratio $k_{\mathrm{TE}}^{\mathrm{abs}}$/<*ρ*> is a proxy for the average elongation rate along the coding sequence. Data information: *P*‐value legend: ****P < 10^−4^.

We studied the consistency between the ribosome density measured by the ribosome profiling data and the absolute translation efficiency derived from the omics quantifications. In the framework of the initiation‐limited ribosome flow model, the average ribosome density of a gene is related to the translation efficiency and the average elongation rate by the simple expression <*ρ*_*i*_> = $k_{\mathrm{TE}}^{\mathrm{abs}}$/<*k*_*elong,i*_>. As suggested by previous studies (Shah *et al*, 2013; Li *et al*, 2014), we expect most of the variations in the local elongation rate along a coding sequence to average out at the gene level, such that the average elongation rate will vary little among genes. In this case, we would expect the ribosome density to increase linearly with the translation initiation rate. Unexpectedly, the ribosome density at the gene level did not correlate with $k_{\mathrm{TE}}^{\mathrm{abs}}$, neither for labeled (Pearson correlation *r* = 0.01, *N* = 67, *P* = 0.96, two‐sided test) nor for unlabeled proteins (*r* = 0.09, *N* = 407, *P* = 0.06). Moreover, we observed that while $k_{\mathrm{TE}}^{\mathrm{abs}}$ spanned a 129‐fold range, the ribosome density only varied 6.5‐fold (Fig 6A).

On one hand, several genes exhibited a high translation efficiency while maintaining an average ribosome density similar to the median of all the other genes. For example, the outB gene (MPN562), coding for a probable NH_3_‐dependent NAD^+^ synthetase, had the highest translation efficiency of all genes (0.97 s^−1^), producing at steady‐state the amount of 743 protein copies per cell (percentile 94) from a moderately abundant mRNA (0.021 copies per cell, percentile 39). However, its density of ribosomes remained very similar to the other genes, with 0.016 codon^−1^ (percentile 47). In order to produce such a moderate ribosome density, the average translation elongation rate in the outB coding sequence must be higher than in the other genes.

On the other hand, several other genes exhibited a low translation efficiency, while also maintaining an average ribosome density similar to the median. For example, the *ftsA*‐like gene (MPN316), involved in cell division, had a very low translation efficiency (0.004 s^−1^, percentile 3), producing at steady‐state only 12.8 protein copies per cell (percentile 13) from an abundant mRNA (0.20 copies per cell, percentile 94). Its density of ribosomes was very similar to the median, 0.020 codon^−1^ (percentile 63). Thus, in order to achieve this moderate ribosome density, the average elongation rate in the *ftsA*‐like coding sequence must be smaller than in the other genes.

These results suggest the existence of large differences in the average elongation rate from gene to gene. Following our modeling framework, we analyzed the ratio $k_{\mathrm{TE}}^{\mathrm{abs}}$/<*ρ*>, which we considered a reliable proxy for the gene‐average elongation rate in the case of proteins quantified by labeled peptides. Among the 73 labeled proteins, the ratio spanned a 40‐fold range from 0.69 codon·s^−1^ to 18.6 codon·s^−1^ (percentiles 5 and 95). This range was very similar when considering all proteins (0.55–18.7 codon·s^−1^, percentiles 5 and 95). We further investigated the relationship between elongation and initiation rates by plotting the ratio $k_{\mathrm{TE}}^{\mathrm{abs}}$/<*ρ*> (average elongation rate) against the absolute translation efficiency $k_{\mathrm{TE}}^{\mathrm{abs}}$ (initiation rate) (Fig 6B). Surprisingly, the Pearson correlation between the two quantities was very high, both for labeled proteins (*r* = 0.96, *N* = 67, *P* = 10^−39^) and for label‐free proteins (*r* = 0.80, *N* = 407, *P* = 10^−91^). Because these two variables are dependent, part of the observed correlation can be due to the positive covariance between $k_{\mathrm{TE}}^{\mathrm{abs}}$ and $k_{\mathrm{TE}}^{\mathrm{abs}}$/<*ρ*>.

In order to estimate the magnitude of this effect, we considered the experimental values of $k_{\mathrm{TE}}^{\mathrm{abs}}$ and simulated the ribosome density data <*ρ*> under the assumption that the elongation rates were constant and equal to the median for all genes (null model), $k_{\mathrm{TE}}^{\mathrm{abs}}$/<*ρ*> = 4.11 codon·s^−1^. In this situation, the expected correlation between $k_{\mathrm{TE}}^{\mathrm{abs}}$/<*ρ*> and $k_{\mathrm{TE}}^{\mathrm{abs}}$ would be zero in the absence of noise (Appendix Fig S12A). When adding simulated noise to $k_{\mathrm{TE}}^{\mathrm{abs}}$, the Pearson correlation between the two quantities increased up to reaching a maximum at *r* ≈ 0.6, both for unlabeled proteins and for labeled proteins (Appendix Fig S12B–D). Moreover, we would expect the correlation due to positive covariance to be stronger for unlabeled proteins, which had a higher variability in the measurement of $k_{\mathrm{TE}}^{\mathrm{abs}}$, compared to labeled proteins. However, we observed the opposite. Therefore, while part of the observed correlation is probably due to the covariance between the two variables, the latter cannot fully explain the very high correlation between the translation efficiency and the ratio $k_{\mathrm{TE}}^{\mathrm{abs}}$/<*ρ*>. Overall, this result suggests the existence of some coordination between the average elongation rate and the initiation rate (see Discussion).

We investigated which features of the mRNA could influence the elongation rate at the gene level. The ratio $k_{\mathrm{TE}}^{\mathrm{abs}}$/<*ρ*> did not correlate with any of the features known to influence elongation rates, such as the codon adaptation index (CAI) (Appendix Fig S13A), GC content (Appendix Fig S14A–D), tAI derived from the measured tRNA abundances (Appendix Fig S13C), folding energy along the CDS (Appendix Fig S13B), and presence of internal SD‐like motifs (Appendix Fig S13E and F). The RNA degradation rate showed no correlation with the ratio $k_{\mathrm{TE}}^{\mathrm{abs}}$/<*ρ*> (Appendix Fig S13D) (*r* = −0.08, *N* = 462, *P* = 0.08). Contrariwise to its effect on local elongation rate, the fraction of negatively charged residues showed a weak positive correlation with the ratio $k_{\mathrm{TE}}^{\mathrm{abs}}$/<*ρ*> (Appendix Fig S15B) (*r* = 0.17, *N* = 474, *P* = 2.3 × 10^−4^). We also found a weak positive correlation with the fraction of polar residues (Appendix Fig S15C) (*r* = 0.14, *N* = 474, *P* = 1.8 × 10^−3^) and a weak negative correlation with the fraction of hydrophobic residues (Appendix Fig S15D) (*r* = −0.22, *N* = 474, *P* = 1.3 × 10^−6^), while no correlation was found for positively charged (Appendix Fig S15A) and hydrophobic residues (Appendix Fig S15E).

### Translational coupling

*Mpn* contains a substantial fraction of overlapping genes in the same operon (26%), i.e. which are transcribed into polycistronic transcripts from a common promoter. In general, translation can be initiated independently on the genes of a polycistronic mRNA, each gene having its own translation initiation region and therefore its own translation efficiency (Li *et al*, 2014). In some cases, however, the translation of the downstream gene can depend on the translation of the upstream gene, a phenomenon called translational coupling (Levin‐Karp *et al*, 2013). Two main mechanisms responsible for translation coupling in bacteria have been proposed (Huber *et al*, 2019): (i) *de novo* initiation whose efficiency depends on the translation of the upstream gene, often via secondary structure, and (ii) translation termination‐reinitiation, in which a ribosome that terminates at an upstream gene initiates translation at a nearby downstream gene without dissociating from the mRNA (or at least not the small 30S subunit).

**Figure 7:**
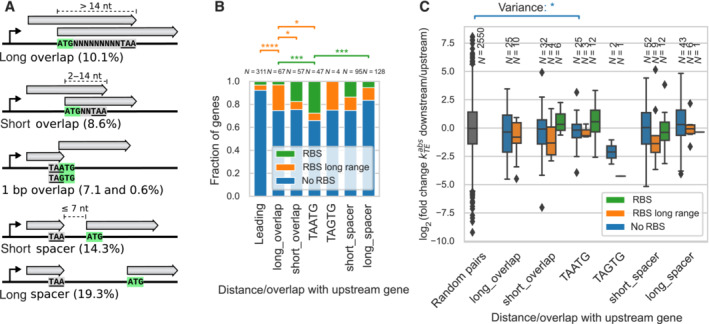
Translational coupling The 398 pairs of consecutive genes in the same operon were classified by their distance between the stop codon and the start codon and their overlap: distance ≤ −15 bp where the 5′ of the downstream gene overlaps with the 3′ of the upstream gene (long_overlap), −14 ≤ distance ≤ −2 (short_overlap), distance −1 where the **ATG** start codon overlaps with the TAA stop codon (TA**A** **TG**), distance −1 where the **GTG** start codon overlaps with the TAG stop codon (TA**G** **TG**), 0 ≤ distance ≤ 7 bp (short spacer), distance > 7 bp (long spacer). Percentages indicate the frequency of gene pair overlap categories in the genome.For each overlap category, the fraction of downstream genes that harbor a SD motif (RBS), or a distant SD motif up to 60 nt upstream of the start codon (RBS long range), are compared to the fraction that does not harbor any motif (no RBS). Genes with the TA**A** **TG** overlap showed a higher fraction of SD motifs compared to the genes with full overlap (Fisher exact test two‐sided, *P* = 0.007) and to the genes with long spacer (*P* = 3 × 10^−4^). Leading genes at the first position in the operon are also shown for comparison (leading).Distribution of the log_2_ fold changes in absolute translation efficiency $k_{\mathrm{TE}}^{\mathrm{abs}}$ between downstream and upstream genes, for the 243 gene pairs within the same operon. The variance of the log_2_ fold changes was smaller in the case of gene pairs with TA**A** **TG** overlap compared to random gene pairs (Levene test, *P* = 0.04). For box‐and‐whiskers, central band denotes the median, bottom and top edges of the box indicate the first quartile and third quartile, respectively. The whiskers extend to the most extreme data points not considered outliers. The data between the first quartile −1.5 × interquartile range and third quartile +1.5 × interquartile range are considered not outliers. Data information: *P*‐value legend: *P < 0.05, ***P < 10^−3^, ****P < 10^−4^.

We examined whether the translation efficiencies of pairs of genes were related. First, we classified pairs of genes by the presence and type of overlap (Fig 7A), as well as the presence of a RBS for the downstream gene. In *Mpn*, because the TGA codon encodes for Trp, the 4 bp overlap **A** **TG**A (**start codon**, stop codon) frequently observed in other bacterial species is absent.

For each category of overlap, we examined the presence of a SD motif close to the start codon of the downstream gene. Overall, the frequency of SD for non‐leading genes on polycistronic mRNAs was lower than for most other bacterial species (Huber *et al*, 2019). Remarkably, the frequency of SD motifs at leading genes (3.2%) was lower than for genes at downstream positions (11.3%, Fisher exact test two‐sided *P* = 5.3 × 10^−5^). Downstream genes with the TA**A** **TG** (stop codon, **start codon**) overlap showed a higher fraction of SD motif (28%) (Fig 7B), followed by genes with a short overlap (17.5%), and genes with a short spacer (13.7%). The other categories contained fewer SD motifs compared to the 1 bp overlap: genes with long spacers (5.5%, *P* = 1.6 × 10^−4^), and genes with long overlap (3.0%, *P* = 1.6 × 10^−4^). These results suggest that the SD motif only plays a role in the translation initiation of genes at downstream positions in the operon, and in particular for genes with a short distance between the stop and start codons. The particularly high fraction of SD motifs for genes with the 1 bp overlap also suggests that SD motifs might enhance coupling by the termination‐reinitiation mechanism.

Moreover, for some genes lacking a canonical SD motif, we also detected the presence of SD motifs at a longer range upstream of the start codon, up to 60 nt. The downstream genes with long overlap seemed to have a more long‐range SD motif (22.4%) (Fig 7B), followed by genes with short spacer (11.5%) and genes with long spacer (10.9%). The other categories had significantly less long‐range SD motifs: genes with short overlap (7.0%, *P* = 0.02), genes with the TA**A** **TG** overlap (6.4%, *P* = 0.03), and leading genes (4.5%, *P* = 1.3 × 10^−5^). Thus, the usage of long‐range SD motifs, positioned farther away from the start codon compared to canonical SD motifs, seemed to be particularly frequent for genes with a longer distance between the start codon and the stop codon of the upstream gene.

Next, we analyzed how the absolute translation efficiency varied between gene pairs. In the presence of tight translational coupling, we expect the translation initiation rate of the downstream gene to be approximately equal to the initiation rate of the upstream gene. Therefore, we analyzed the log_2_ fold change (log_2_FC) between the translation efficiencies of the downstream gene with respect to the upstream gene, for 243 gene pairs (Fig 7C). We found that for gene pairs with the TA**A** **TG** overlap and no SD motif, the variance of the distribution of log_2_FC was significantly smaller compared to the random gene pairs in the genome (Levene test, *P* = 0.04). The statistical power of this analysis was limited by the small number of gene pairs with $k_{\mathrm{TE}}^{\mathrm{abs}}$ quantifications. Although some differences were not statistically significant, we also observed that the presence of a SD motif for gene pairs with the TA**A** **TG** overlap and gene pairs with a short overlap increased the log_2_FC. This suggests that the SD motif may increase the translational coupling only when the distance between the stop and start codons is very short or overlapping, probably favoring the termination‐reinitiation mechanism. Contrariwise, the presence of a SD motif far upstream of the start codon seemed to decrease translation efficiency for gene pairs separated by a short spacer.

## Discussion

In this study, we applied the ribosome profiling method for the first time to *Mpn*. The changes in ribosome conformation during the stages of elongation have been suggested to be one of the causes of the heterogeneity of footprint lengths in bacteria (Mohammad *et al*, 2019). Moreover, the typical length of RPFs may depend on the ribosome structure and its associated factors, specific to each bacterial species. In most *E. coli* libraries, RPF lengths are centered around 24 nt (Mohammad *et al*, 2016, 2019). In eukaryotes, elongating ribosomes yield 28 nt RPFs (Ingolia *et al*, 2009), whereas in archaea their typical length is 27 nt (Gelsinger *et al*, 2020). Despite differences, the typical length of footprints in the *Mpn* samples was very similar to the ones previously described in *E. coli*. The calibration showed that the ribosome P‐site was located at a distance of 19 nt from the 3′ end of footprints, compared to 15–16 nt in *E. coli* (Marks *et al*, 2016; Nakahigashi *et al*, 2016). This might be explained by the differences in ribosome structure between the two species. *Mpn* ribosomal proteins L22, L23, and L29 possess C‐terminal extensions that are not present in *E. coli*. Homology modeling of the *Mpn* ribosome structure guided by cross‐linking mass spectrometry and cryo‐electron tomography suggests that those extensions are located close to the ribosomal exit tunnel (O'Reilly *et al*, 2020). These extensions could protect a few additional nucleotides of the mRNA fragment from the nuclease digestion compared to the *E. coli* ribosome.

In the metagene profile, apart from the expected peak of ribosome occupancy at the start codon, a secondary peak was also observed at position +14 nt, both for samples treated with tetracycline and with chloramphenicol. The origin of this peak remains unclear. Excluded from the analysis, leaderless‐like transcripts cannot be responsible for this peak. A potential error in the P‐site shift can also be discarded: if the peak at +14 nt would correspond to the fragments protected by the initiating ribosome, most of those fragments would have their 5′ end downstream the start codon. Importantly, because we discarded the first 15 codons of all CDSs in our analyses, the presence of this secondary peak had no impact on the estimation of translation efficiency.

The use of chloramphenicol (Cm) as a translation inhibitor has been suggested to arrest ribosome elongation in a sequence context‐specific manner and to alter the codon pausing landscape (Nakahigashi *et al*, 2014; Marks *et al*, 2016; Mohammad *et al*, 2016). In particular, Cm‐dependent pauses were observed when Ala, Gly, and Ser codons are positioned in the E site (Mohammad *et al*, 2019). In our metafootprint analysis, we observed a particularly high relative ribosome density at position −1 (E‐site) for some Gly codons (in particular GGA and GGG). However, the other Gly codons, Ser and Ala codons showed a relative density similar to the gene average. The same trend was also observed in the *E. coli* sample. These results, together with the fact that the pattern of the relative ribosome densities for A‐site codons in the *E. coli* sample was different from the pattern in *Mpn* samples, indicate that at least part of the observed ribosome density pattern was not due to chloramphenicol‐related biases.

One possible limitation of our study is that the small genome size of *Mpn* may reduce the statistical power to detect determinants of translation efficiency.

At the gene level, while the number of genes in *Mpn* is smaller than in other organisms (∼700 compared to ∼4,600 in *E. coli* and ∼7,600 in yeast), the proportion of genes for which we quantified the absolute translation efficiency was higher compared to other studies in yeast, 67% compared to 14% (Lahtvee *et al*, 2017; Riba *et al*, 2019). The total number of genes used for the analysis of determinants in our study was only 2.3 times smaller (475 genes) compared to the other studies (∼1,110 genes), such that the difference in statistical power with similar studies in yeast should be moderate.

At the codon level, the total size of the coding genome in *Mpn* (∼0.6 M nt) is 6.5 times smaller than in *E. coli* (4.1 M nt), and 14 times smaller than in yeast (∼9 M nt) (Nagalakshmi *et al*, 2008), which could reduce the statistical power for the analysis of the determinants of local elongation rate. To this regard, sequencing depth of the ribosome footprints library is one of the major factors controlling noise. In our study, the average number of footprint reads after filtering per codon in the transcriptome was 7.6 (rep1) and 2.7 (rep2), which was similar to other studies in yeast (∼0.1–90 RPFs per codon; Diament & Tuller, 2016) and in *E. coli* (∼5–10 RPFs per codon; Mohammad *et al*, 2019).

*Mpn*, like several mycoplasma species, possesses an unconventional initiator tRNA (fMet‐tRNA), where the highly conserved sequence of GC/GC/GC base pairs in the anticodon stem is replaced by AU/GC/GU (Ayyub *et al*, 2018). Selection of the initiator tRNA at the P‐site is crucial for the correct recognition of start codons and subsequent formation of the initiation complex (Shetty *et al*, 2017), and the structure of the anticodon stem loop is essential for the initiator tRNA function. Studies in *E. coli* showed that strains with an AU/GC/GU stem mutant initiator tRNA display substantially impaired growth (Samhita *et al*, 2012). In addition, *Mpn* displays polymorphisms in the initiation factor IF3 that have been associated with a reduced fidelity of translation initiation at canonical start codons. *Mpn* has evolved compensatory genetic variations in the rRNA, ribosomal proteins uS9 and uS13, and initiation factor IF3 regions that interact with the fMet‐tRNA at the P‐site (Ayyub *et al*, 2018). We found that the initiator fMet‐tRNA was the most abundant tRNA in *Mpn*, building up to 12.1% of all tRNA species. In *E. coli*, the relative abundance of the two fMet‐tRNAs represents ∼3% of the total tRNA content (Dong *et al*, 1996), but this proportion can increase with the growth rate. Thus, *Mpn* has a much higher relative level of fMet‐tRNA. We speculate that this high abundance could alleviate the lower efficiency and precision of the recruitment of the fMet‐tRNA at the initiation site and reduce spurious translation initiation at non‐canonical start codons.

The excess of arginyl‐tRNA‐synthetase, which was 5 times more abundant than the other aaRSs, remains intriguing. We speculate that this could be related to the loss of the tRNA‐A34 deaminase (tadA) in *Mpn*, a tRNA modification enzyme responsible for the formation of wobble inosine in other bacterial species (Grosjean *et al*, 2014). Due to the loss of this enzyme, *Mpn* does not possess the highly efficient Arg‐tRNA‐ICG carrying a wobble inosine (Novoa *et al*, 2012), which could require higher concentrations of Arg tRNAs, or, as happens in *Mpn*, higher abundance of the arginyl‐tRNA‐synthetase to increase the charging of tRNA^Arg^s.

The frequency of SD sequence in *Mpn* (∼8%) is much lower than in other bacterial species such as *E. coli* (57%; Ma *et al*, 2002). Indeed, the proportion of genes that contain a SD motif varies widely among bacterial species (Nakagawa *et al*, 2010), suggesting that SD interaction is not essential for translation initiation in many prokaryotes. In *Mpn*, we found that the translation efficiency of genes with and without a SD motif was similar. This was the case both for leading genes and for non‐leading genes on polycistronic mRNAs (Appendix Fig S16). This observation, together with the prior results of a library of randomized 5′UTR (Yus *et al*, 2017), strongly suggests that the SD motif has no influence on translation efficiency in *Mpn*.

The SD sequence utilization across bacterial species has been linked to the growth rate (Hockenberry *et al*, 2018), such that fast‐growing bacterial species present a higher proportion of genes with a SD motif. Organisms capable of rapid growth have high protein production demands. The higher SD sequence utilization in these species might thus be related to more efficient translation initiation. *Mpn*, with a doubling time of 8 h, likely did not experience such a pressure which might explain its lower frequency of SD, which increases in faster dividing mycoplasma species (Montero‐Blay *et al*, 2019). Intriguingly, in *Mpn*, genes encoding for ribosomal proteins showed a much higher utilization of the SD sequence compared to the other genes (32% compared to 6%), while their translation efficiencies were similar (Appendix Fig S3D). We speculate that the presence of the SD motif, in the case of ribosomal genes, might play a role in translation coupling within polycistronic mRNAs, rather than increasing translation initiation rate.

We also detected the presence of SD motifs at a longer range upstream the start codon (up to 60 nt). These motifs were more frequent for genes with a longer distance between the start codon and the stop codon of the upstream gene. We hypothesize that these motifs may favor *de novo* translation initiation on the downstream gene, by recruiting ribosomes to the mRNA, or by slowing down elongating ribosomes on the upstream gene, which may influence translation initiation on the downstream gene, for example by destabilizing secondary structure blocking the accessibility of the downstream start codon.

Differences in the mode of translation initiation between species may be related to the different ribosomal protein compositions. For example, most mollicutes lack the ribosomal protein S1 (RPS1) (Grosjean *et al*, 2014). In *E. coli*, RPS1 interacts with the 5′UTR of a mRNA and plays an important role in increasing translation efficiency, regardless of the presence of the SD sequence (Komarova *et al*, 2005). RPS1 binds to an AU‐rich sequence upstream of the start codon, and promotes translation initiation by unfolding the mRNA structure (Qu *et al*, 2012; Romilly *et al*, 2019) and protecting the mRNA from degradation, probably by competing against RNase E binding (Lee *et al*, 2021). The lack of RPS1 in *Mpn* might be one of the reasons why the AU‐content upstream of the start codon was not related to translation efficiency.

The other mechanism of translation initiation is for leaderless transcripts that lack a 5′UTR and involves pre‐formed 70S ribosomes (Balakin *et al*, 1992; Moll *et al*, 2004). First discovered in *λ*‐phage *E. coli*, the frequency of leaderless transcripts varies widely across bacterial species, and is particularly high in Actinobacteria (∼40%) and some mycoplasmas (∼18%) (Zheng *et al*, 2011; Shell *et al*, 2015). In *E. coli*, initiation by 70S ribosomes requires an AUG start codon close to the 5′ end of the mRNA (Krishnan *et al*, 2010). The frequency of leaderless‐like transcripts (length of 5′UTR ≤ 8) in *Mpn* (16%), i.e. with a 5′UTR too short to harbor a RBS, is similar to other mycoplasmas. We found that the translation efficiency of leaderless‐like genes was similar to leadered genes (Appendix Fig S4B). Moreover, we found no clear association between the translation efficiency and the length of the 5′UTR (Appendix Fig S3E). Similar results were described in *Mycobacterium tuberculosis* (Nguyen *et al*, 2020) and in *Caulobacter crescentus* (Schrader *et al*, 2014), two bacterial species with a high frequency of leaderless transcripts. Our results suggest that translation initiation on leaderless mRNAs is a common mechanism of translation in *Mpn* during normal growth, in contrast to *E. coli* where this mode of translation is active during the response to stress (Vesper *et al*, 2011).

By computing the ratio between the absolute translation efficiency and the ribosome density, we investigated how the gene‐level average elongation rate varied across genes. We observed 4 genes with extremely high elongation rates (> 18 codons·s^−1^). We found that the gene with the highest elongation rate, MPN562, showed one of the largest deviations from the absolute protein calibration curve. This suggests that the extreme values of elongation rates for these 4 genes were due to biases in the protein quantification approach based on a single labeled peptide (Li *et al*, 2012; Oeckl *et al*, 2018). Thus, by discarding outliers, we found a 40‐fold variation in elongation rate from 0.69 codon·s^−1^ to 18.6 codon·s^−1^ (percentiles 5 and 95, labeled proteins). A similar study in yeast showed that the average elongation rates varied ∼20‐fold among CDSs (Riba *et al*, 2019) using the pSILAC method.

We found a strong positive correlation between the translation efficiency, equivalent to the translation initiation rate in our modeling approach, and the gene‐level elongation rate. This observation did not derive from any of our modeling assumptions, but rather from the relationship between the measured ribosome density and the absolute translation efficiency derived from omics data. This result suggests that the elongation rate adapts to the translation initiation rate such as to maintain the ribosome density approximately constant across genes. The mechanism enabling this possible adaptation remains unknown, as no strong correlation was found between the gene‐level elongation rate and any of the mRNA features known to impact the elongation. One possible artifact that could contribute to this correlation would be that part of the reads in the ribosome profiling library originated from mRNA fragments not associated with a ribosome, which would mask the ribosome density signal and artificially reduce its dynamic range. A similar correlation was observed in yeast (Riba *et al*, 2019), where highly expressed CDSs were found to have high elongation rates in general. Interestingly, a recent study in the filamentous fungus *Neurospora crassa* (Lyu *et al*, 2021) showed that the phosphorylation of initiation factor eIF2α specifically inhibits translation initiation of mRNAs with long CDS and non‐optimal codon usage, which suggests a mRNA‐preferential feedback mechanism from translation elongation to control translation initiation. In *Mpn*, such a feedback mechanism, whose existence remains speculative, would require a strong local effect to adjust the initiation rate in a mRNA‐specific manner.

Measures of the adaptation of synonymous codon usage, such as the codon adaptation index (CAI) and the tRNA adaptation index (tAI) have been shown to correlate with the elongation rate, in yeast (Riba *et al*, 2019; Szavits‐Nossan & Ciandrini, 2020) and also in *E. coli* (Mohammad *et al*, 2019). However, this correlation is weak in general (*r* < 0.6), which suggests that the local elongation rate is only partially determined by codon adaptation to the tRNA pool. The range of tRNA abundances measured in *Mpn*, 139‐fold, was much larger than the range of average codon elongation rates, 9‐fold. This raises the question of how tRNA abundances influence the codon decoding rates. We did not observe any clear correlation between the tAI derived from the tRNA abundances and the average codon elongation rates derived from the ribosome profiling data. The efficiencies of the different codon‐tRNA interactions can vary among organisms and are difficult to measure. In our calculation of the tAI the base pairing weights were taken from previous studies in yeast and could be inadequate for *Mpn*, which would lead to distortion in the adaptation score of near‐cognate codons. Moreover, the competition between the tRNAs, and not only their abundances, have been suggested to play an important role in the decoding rate of codons, as theoretical studies suggest (Fluitt *et al*, 2007; Chu & von der Haar, 2012; Vieira *et al*, 2016). Synonymous codon order has also been suggested to influence the elongation speed. The putative recycling of tRNAs in the vicinity of the ribosome may lead to faster local elongation rate for subsequent occurrences of codons that use the same tRNA (Cannarozzi *et al*, 2010). This effect, however, remains difficult to isolate from other effects in the statistical analysis of endogenous genes.

In the case of yeast, the presence of positively charged amino acids was shown to induce slower elongation, while negatively charged amino acids were shown to increase elongation rate, both at the gene level (Riba *et al*, 2019) and at the local level (Charneski & Hurst, 2013; Dao Duc & Song, 2018). In our study, we found that negatively charged residues in the window [−15, 14] nt had a weak negative impact on the local elongation rate, while the fraction of negatively charged residues in the whole protein sequence had a weak positive impact on the gene‐level elongation rate. The interplay between charged residues and elongation rate is believed to be mediated by electrostatic interactions between the nascent peptide and the ribosome exit tunnel. However, the effects of these interactions are still poorly understood. The net force exerted on the nascent peptide could depend on the distribution of charges along the protein sequence and on the precise characteristics of the ribosome structure (Joiret *et al*, 2022). Moreover, the structure and electrostatic charge distribution inside the ribosome exit tunnel vary across organisms (Sabi & Tuller, 2015). Although the exact ribosome structure of *Mpn* is not known, the importance of these interactions could be much smaller in *Mpn*.

Although we found clear evidence that internal SD‐like motifs slow down translation elongation locally, we found no differences in the gene‐level elongation rate in the presence or absence of multiple SD‐like motifs in CDSs (Appendix Fig S13E and F). These observations suggest that internal SD‐like motifs, although they may induce local elongation pauses, have little impact on the translation efficiency in *Mpn*.

In this study, we aimed to achieve a quantitative understanding of translation efficiency in a genome‐reduced model bacterium. We integrated the absolute quantifications of protein abundances, mRNA abundances, and protein half‐lives with ribosome density to determine translation initiation and elongation rates of a majority of genes in *Mpn*. Although the initiation rate varied over 160‐fold among genes, most of the known factors had little impact on translation efficiency in *Mpn*. Translation initiation occurred efficiently on a variety of transcripts, leadered or leaderless, with or without a SD motif. Our results suggest that the initiation rate could be weakly negatively influenced by the secondary structure close to the start codon, by a larger fraction of hydrophobic residues at the N‐terminal, while negatively charged residues at the N‐terminal could have the opposite effect. At the local level, the determinants of the rate of translation elongation remained mostly unidentified. We showed that the presence of internal SD‐like motifs induced slower local elongation rates, but had no influence on the gene‐level elongation rate. The identity of the codon at the A‐site had a small but consistent impact on the local elongation rate. In addition, we measured the cellular abundances of all tRNA species. We found that tRNA abundance varied over 100‐fold, but that these variations could not fully explain the A‐site codon elongation rates.

The large differences in translation efficiency among genes and the fact that the elongation rate seems to adapt to the initiation rate point toward the existence of unknown global mechanisms of translation control.

## Materials and Methods

### Reagents and Tools table

**Table:** 

Reagent/resource	Reference or source	Identifier or catalog number
**Experimental models**		
*Mycoplasma pneumoniae* M129	Richard Herrmann lab	
*Escherichia coli* DH5α	NEB	C2987H
**Chemicals, enzymes and other reagents**		
Chloramphenicol	Sigma	C0378
Tetracycline	Sigma	T7660
Digested bovine serum albumin	NEB	P8108S
Sucrose	Sigma	84097
Triton X‐100	Sigma	X100
HEPES	Sigma	H4034
Dnase		
SuperScript II Reverse Transcriptase	Invitrogen	18064‐014
DNA Polymerase I		
RNase H		
MNase		
NP40		
Trizol	Invitrogen	15596026
AgenCourt AMPure XP beads	Beckman Coulter	A63882
Lysozyme		
LysC	Wako	129‐02541
Trypsin	Promega	V5113
^13^C_6_, ^15^N_2_‐Lysine	Thermo Fisher Scientific	88209
^13^C_6_, ^15^N_4_‐Arginine	Thermo Fisher Scientific	89990
Antarctic phosphatase	NEB	M0289
PNK	NEB	M0201
**Software**		
Proteome Discoverer (v2.0)	Thermo Fisher	
Mascot search engine (v2.5)	Matrix Science	
Skewer v0.2.2	Jiang *et al* (2014)	
Samtools (v1.9) (using htslib 1.9)	Li *et al* (2009)	
Bedtools (v2.27.1)	Quinlan and Hall (2010) and Quinlan (2014)	
**Other**		
NEBNext® Small RNA Library Prep Set for Illumina kit	NEB	E7330
EDTA‐free protease inhibitor cocktail	Roche	11873580001
miRNeasy Mini Kit	Qiagen	217004
TruSeq Stranded mRNA Sample Prep Kit	Illumina	20020595, 20020594
NGS Fragment Kit	Agilent	DNF‐473
KAPA Library Quantification Kit	KapaBiosystems, Roche	KK4835, 7960204001
C18 Acclaim PepMap precolumn	Thermo Fisher Scientific	164564
Agilent DNA 1000 Kit	Agilent	5067‐1504
Agilent High Sensitivity DNA Kit	Agilent	5067‐4626
Novex™ TBE‐Urea Gels, 15%	Thermo Fisher Scientific	EC6885BOX
Cell Disruption Vessel	Parr Instrument Company	4369
LTQ‐Orbitrap Velos Pro mass spectrometer	Thermo Fisher Scientific	
BioAnalyzer	Agilent	
HiSeq 2500 sequencing platform (HiSeq v4 chemistry)	Illumina	
Ultracentrifuge OptimaTM L‐100 XP	Beckman Coulter	

### Methods and Protocols

#### Cell culture and treatments

*Mycoplasma pneumoniae* cell cultures were grown in Haylick rich medium as previously described (Yus *et al*, 2009) for 72 h, split into biological duplicates and grown for 6 h (exponential phase, standard conditions). In order to block ribosomes at the initiation stage, cells were treated with 40 μg/ml of tetracycline during 1 min before lysis. Except for tetracycline‐treated samples, cells were treated during 1 min with chloramphenicol at 100 μg/ml final concentration in order to stop translation. After treatment cell media was removed and cells were harvested by scraping and centrifuged at 8,000 *g* during 5 min in a 2 ml tube. Supernatant was discarded and cell pellet was resuspended in 1.5 ml of lysis buffer containing 50 mM Hepes pH7, 100 mM NaCl, 10 mM MgCl_2_, 100 μg/ml chloramphenicol, 1 mM PMSF, 5 mM CaCl2, 1 mM DDT, EDTA‐free protease inhibitor cocktails (ROCHE) and DNAse, once pellet was resuspended lysis pressure was applied using a prechilled 4639 Cell Disruption Vessel (two passes). The cell lysate was centrifuged in a cooled desktop centrifuge at 16,000 *g* for 13 min and the supernatant was frozen into liquid nitrogen for further procedures.

#### Total RNA‐seq library preparation

RNA was isolated using the miRNeasy kit (Qiagen), including the in‐column DNase I treatment, and the quality of RNA assessed using a BioAnalyzer (Agilent). The RNA‐seq libraries were prepared at the CRG Genomics Unit using the TruSeq stranded mRNA Library Prep kit (96 samples ref. 20020595 or 48 samples ref. 20020594) according to the manufacturer's protocol, without the initial polyA selection, to convert total RNA into a library of template molecules of known strand origin and suitable for subsequent cluster generation and DNA sequencing. Briefly, 100 ng of total RNA were fragmented under elevated temperature and primed with random hexamers for cDNA synthesis. The cleaved RNA fragments were copied into first strand cDNA using reverse transcriptase (SuperScript II, ref. 18064‐014, Invitrogen) and random primers. After that, second strand cDNA was synthesized, removing the RNA template and synthesizing a replacement strand, incorporating dUTP in place of dTTP to generate ds cDNA using DNA Polymerase I and RNase H. To these cDNA fragments, a single “A” base was added to the 3′ ends to prevent self‐ligation during the adapter addition. A corresponding single T nucleotide on the 3′ end of the adapter provides a complementary overhang for ligating the adapter to the fragments. Subsequent ligation of the multiple indexing adapter to the ends of the ds cDNA was done. Finally, PCR selectively enriched those DNA fragments that had adapter molecules on both ends. The PCR was performed with a PCR Primer Cocktail that anneals to the ends of the adapters.

Final libraries were analyzed using Bioanalyzer DNA 1000 or Fragment Analyzer Standard Sensitivity (ref: 5067‐1504 or ref: DNF‐473, Agilent) to estimate the quantity and validate the size distribution, and were then quantified by qPCR using the KAPA Library Quantification Kit KK4835 (REF. 07960204001, Roche) prior to the amplification with Illumina's cBot. Sequencing was performed using a HiSeq 2500 (Illumina) with HiSeq v4 chemistry and 2 × 50 bp paired‐end reads.

#### Total RNA‐seq reads processing

Processing of sequencing reads from RNA extracts was performed as follows. Adapter sequences (Illumina TruSeq adapter AGATCGGAAGAGCACACGTCT) were trimmed from paired‐end reads by using the SeqPurge tool (version 0.1‐478‐g3c8651b) (Sturm *et al*, 2016), keeping trimmed reads with a minimum length of 12, and trimming low‐quality bases at the end of the read using a sliding window of size 5 (‐qwin) and a minimum quality score of 15 (‐qcut). Reads were aligned to the reference genome of *M. pneumoniae* M129 (NCBI assembly accession NC_000912.1) using bowtie2 v. 2.2.9 (Langmead & Salzberg, 2012), with parameters values: end‐to‐end mode, 1 mismatches (‐N), very sensitive mode (‐L 20 ‐D 20 ‐R 3 ‐i “S,1,0.50”), maximum fragment length 800 nt (‐X), only best alignment reported (‐k 0). Alignment files were converted from SAM format to sorted indexed BAM format using samtools v. 1.9 (using htslib 1.9) (Li *et al*, 2009) and sort (GNU coreutils) 8.26. Reads were further filtered by keeping only primary and mapped reads, and converted to sorted BEDPE format using samtools and bedtools v2.27.1 (Quinlan & Hall, 2010; Quinlan, 2014). Fragment counts per annotation region were computed using bedtools, with strand specific overlaps with minimum overlap fraction of 0.5 of read length. Finally, strand‐specific per‐base coverage was computed using bedtools.

#### Optimization of ribosome profiling protocol in *Mpn*

*Mpn* present some characteristics that differ from other model bacterial species where ribosome profiling has been established, like the absence of a cell wall, different lipid composition and low number of ribosomes (200–300 per cell (Seybert *et al*, 2006; Maier *et al*, 2011), compared with ∼55,000 in *E. coli* (Bakshi *et al*, 2012)). Thus, several aspects of the original method (Ingolia *et al*, 2009) had to be adapted. As a control, we also performed ribosome profiling in *E. coli*.

The choice of the methods used to arrest translation and harvest cells has been shown to be critical for obtaining reliable ribosome positions that faithfully reflect the *in vivo* translational landscape. Antibiotics, such as cycloheximide in the case of yeast and chloramphenicol in the case of *E. coli*, may inhibit ribosome elongation in a context‐dependent manner (Nakahigashi *et al*, 2014; Marks *et al*, 2016; Mohammad *et al*, 2016), introducing biases in the ribosome density landscape. Fast cell collection methods such as rapid filtration followed by fast freezing in liquid nitrogen have been used to stop translation without the use of any antibiotic (Mohammad *et al*, 2019). However, this method is not always applicable to all bacterial species. In *Mpn*, cell aggregation in liquid culture prevents the use of rapid filtration and creates stress conditions for the cells such as nutrient deprivation. Therefore, we chose chloramphenicol treatment in cell culture in order to arrest translation.

#### Polysome profiling by sucrose gradient

We tested nuclease digestion both in *E. coli* (as control) and *Mpn* and we analyzed the efficiency of digestion through a sucrose gradient. Using this technique and comparing digested and undigested samples we could assess the polysome profile. In Appendix Fig S17B (left), a sucrose gradient is shown from *E. coli* samples comparing digested and undigested supernatant lysate. Upon MNase treatment the third peak (monosomes) presents an increase, whereas the polysome fraction decreases.

Applying the same treatment in *Mpn* samples produced the gradient profile shown in Appendix Fig S17B (right panel). In this case, three peaks were not observed, indeed only two peaks appeared, probably the 30 and 50S subunits. Additionally, no major difference between digested and undigested samples could be appreciated. This result in *Mpn* polysome profiling could indicate the disassembly of ribosomes during the experimental manipulation.

The 70S ribosome complex is maintained mainly through ionic interactions, and high NaCl concentration can perturb this kind of interactions. In our laboratory, we have noticed that *M. pneumoniae* requires less salt concentration to break ionic interactions of nucleic acid binding proteins in comparison with other organisms (unpublished results). For that reason, we reduced the NaCl concentration in the sucrose gradient buffer. A comparison between buffers with high, standard NaCl concentration (400 mM) and low NaCl concentration (200 mM) is shown in Appendix Fig S17C. When the gradient was performed with low NaCl (200 mM) only one peak appeared at a denser fraction of the gradient indicating the presence of the 70S complex. Taking into account these results, it seems that monosomes can be isolated under these conditions, and polysomes cannot be detected by this method. Therefore, 200 mM NaCl was further used for the “preparative” sucrose cushions. The fact that only one peak was observed could indicate that all the ribosomes are found as 70S units and thus very low amounts of 30S and 50S are found.

#### Sucrose cushion optimization

In order to obtain a sufficient amount of ribosomes for the library preparation, we optimized the conditions of the sucrose cushion, decreasing the sucrose concentration from 25% used in *E. coli* (Becker *et al*, 2013) to 15%. With these optimized conditions, the proportion of RNA recovered after purification of the ribosomes in the *Mpn* sample was similar to the proportion recovered in the *E. coli* sample (see Appendix Fig S18).

Sucrose cushion is a centrifugation‐based technique that allows us to isolate ribosomes and polysomes due to their high molecular weight compared to other cell components. In addition, sucrose cushion requires less specialized equipment and is more scalable than sucrose gradients. We applied this technique to isolate ribosomes in *M. pneumoniae*, using 25% sucrose cushion as described for other organisms as *E. coli* (Becker *et al*, 2013). In order to quantify the amount of ribosomes recovered, we measured the total RNA loaded on the cushion and also the RNA found in the cushion pellet (termed P2) after centrifugation as an estimate of the ribosome recovery. We compared samples digested with MNase and non‐digested, expecting to obtain more RNA amount when the samples were not digested. We plotted a comparison bar plot (Appendix Fig S18) of RNA recovery from P2 both in *E. coli* and in *M. pneumoniae*. In both cases, we observed more RNA recovery when samples were not digested, although, significantly less percentage of RNA amounts were obtained in *M. pneumoniae* when compared with *E. coli* samples.

In *E. coli*, 25% sucrose cushion (with the centrifugation conditions mentioned in the methods section) has been shown to pellet 70S complexes. We used the same conditions in order to isolate 70S complexes from *Mpn* but we observed a lower recovery compared to *E. coli* (Appendix Fig S18) indicating an important loss of ribosomes during this process. Thus, we hypothesized that the % of sucrose could influence the recovery of ribosomes. To find the optimal % of sucrose in which the 70S recovery was maximal for *Mpn*, we performed a sucrose gradient comparing the sedimentation coefficient of *E. coli* and *Mpn*. We observed a higher sedimentation coefficient and therefore, higher molecular weight for *E. coli* ribosome subunits in comparison with those coming from *M. pneumoniae*. Based on this observation, we estimated that the optimal % of sucrose to recover *Mpn* ribosomes had to be reduced to 15%. We repeated the sucrose cushion with this new condition and we observed a higher recovery of RNA close to the one observed for *E. coli* (Appendix Fig S18).

#### Sucrose gradients, and fractions collection

Cell lysates, either digested or not with MNase, were loaded onto a 10–50%, or 5–40%, sucrose gradient in buffer containing 50 mM Tris pH 7, 20 mM MgCl_2_, 100 μg/ml chloramphenicol, 1 mM PMSF and 40 mM NaPO_4_ and either 200 mM, or 400 mM NaCl. Gradients were centrifuged in an ultracentrifuge OptimaTM L‐100 XP (Beckman Coulter) using a SW40 rotor at 217,000 *g* (35,000 rpm) for 2.5 h at 4°C. Gradient absorption was measured at 254 nm, and fractions containing ribosomes or polysomes were collected and flash‐frozen in liquid nitrogen, samples were kept at −80°C until further use.

#### Ribosome and ribosome footprint isolation

In order to get ribosome footprints, a controlled digestion with MNase was performed as described in (Becker *et al*, 2013). Digestion reaction was stopped with 6 mM of EGTA. With the purpose of isolating ribosomes, a sucrose cushion was performed. We loaded the supernatant lysate onto 45 ml of 25% (for *E. coli* cells) or 15% (for *Mpn* cells) sucrose cushion buffer containing 50 mM Tris pH 7, 200 mM NaCl, 20 mM MgCl_2_, 100 μg/ml chloramphenicol, 1 mM PMSF and 40 mM NaPO_4_ pH 7 and ultracentrifuged the samples at 235,000 *g* during 4 h and 30 min at 4°C in a TI‐45 rotor Beckman. The cushion buffer was removed carefully with a vacuum system and the pellet containing ribosomes was resuspended with 200 μl of wash buffer containing 50 mM Tris pH 7, 250 mM NaCl, 10 mM MgCl_2_, 1 mM PMSF, 10 μg/ml chloramphenicol, 0.4% (vol/vol) Triton and 0.1% (vol/vol) NP40. Finally, 900 μl of TRIzol were added in order to perform RNA extraction (manufacture instructions). Once the RNA was purified, we isolated the ribosomes footprints following the steps described in (Becker *et al*, 2013) in supplementary methods, page 6, sections “Size selection of the footprint fragments” and “Dephosphorylation”.

#### Ribosome profiling library preparation

Libraries were prepared using the NEBNext® Small RNA Library Prep Set for Illumina® kit (ref. E7330) according to the manufacturer's protocol. Briefly, 50 ng of RNA were subjected to adaptor ligation at 3′ and 5′ and first strand cDNA synthesis. After that, PCR selectively enriched those DNA fragments that had adapter molecules on both ends. Library amplification was performed by PCR using NEBNext® Multiplex Oligos for Illumina (Index Primers Set 1, ref. E7335). All purification steps were performed using AgenCourt AMPure XP beads (ref. A63882, Beckman Coulter). Final libraries were analyzed using Agilent Bioanalyzer (ref. 5067‐4626) to estimate the quantity and check size distribution. Libraries were quantified by qPCR using the KAPA Library Quantification Kit (ref. KK4835, KapaBiosystems) prior to amplification with Illumina's cBot. Sequencing was performed using a HiSeq 2500 (Illumina) with HiSeq v4 chemistry and 1 × 50 bp single‐end or 2 × 50 bp paired‐end reads.

#### Ribosome profiling reads processing

Processing of sequencing reads from ribosome footprints was performed as follows. Unless specified, the same pipeline was used for single‐end (SE) and paired‐end (PE) reads. As most footprint fragments were shorter than the read length, the adapter sequences must be removed from the reads. For PE reads, adapter sequences (forward adapter AGATCGGAAGAGCACACGTCT, reverse adapter GATCGTCGGACTGTAGAACTCTGAACGTGTAGATCTCGGTGGTCGCCGTA) were trimmed by using the SeqPurge tool (version 0.1‐478‐g3c8651b) (Sturm *et al*, 2016), keeping trimmed reads with a minimum length of 12, and trimming low‐quality bases at the end of the read using a sliding window of size 5 (‐qwin) and a minimum quality score of 15 (‐qcut). For SE reads, adapter sequences were trimmed by using skewer v0.2.2 (Jiang *et al*, 2014), keeping trimmed reads with a minimum length of 12 (‐l), and trimming low‐quality bases at the end of the reads with a minimum quality score of 10 (‐q). Reads were aligned to the reference genome of *M. pneumoniae* M129 (NCBI assembly accession NC_000912.1) using bowtie2 v. 2.2.9 (Langmead & Salzberg, 2012), with parameters values: end‐to‐end mode, 1 mismatches (‐N), length of the seed substring 12 (‐L), very sensitive mode (‐D 20 ‐R 3 ‐i “S,1,0.50”), maximum fragment length 400 nt (‐X), only best alignment reported (‐k 0). Alignment files were converted from SAM format to sorted indexed BAM format using samtools v. 1.9 (using htslib 1.9) (Li *et al*, 2009) and sort (GNU coreutils) 8.26. Reads were further filtered by keeping only primary and mapped reads, filtering out reads mapping to rRNA genes, and converted to sorted BED or BEDPE format using samtools and bedtools v2.29.2 (Quinlan & Hall, 2010; Quinlan, 2014).

#### Footprints P‐site shift calibration

We first analyzed the metagene profile of footprints from the sample treated with tetracycline. To better control for differences in P‐site shift variations associated with the footprint length, we stratified footprints by length and analyzed them separately (Lauria *et al*, 2018). Metagene profiles were computed by aligning CDSs to the first nucleotide of the start codon, and computing the cumulative coverage of either the 5′, 3′ or midpoint of the footprints at the nucleotide resolution. In order to reduce the impact of noise from CDSs with lower levels of mRNA and/or ribosomes, footprint counts were not normalized. Due to the compact genome of *Mpn*, overlapping of genes is frequent (22% of CDSs overlap on the same strand by at least one base pair) and footprints from ribosomes translating the upstream CDS close to the start codon can introduce artifacts in the calibration. In order to avoid the interference from overlapping CDSs, we selected genes at the first position in their operon (Yus *et al*, 2019) and which had a 5′UTR longer than 8 nt (leadered genes) without any overlapping gene.

The metagene profiles were examined manually to determine the position of peaks close to the start codon. For most footprint lengths, the metagene profiles showed a peak for the 3′ end of footprints at a distance of 19 nt from the first nucleotide of the start codon, or at a distance of 21 nt for footprints of length 35 nt or longer (Dataset EV1). Metagene profiles for the chloramphenicol‐treated samples in exponential phase showed a similar albeit smaller peak at the same position from the start codon, supporting the calibration. Unexpectedly, for footprints longer than 30 nt, a second peak could be observed at 33 nt for the 3′ mapping, although only for some combinations of footprint lengths and samples. The ribosome P‐site shift from the 3′ end of footprints was chosen (Dataset EV1) guided by the observed peaks, while trying to harmonize the offset across the different footprint lengths to avoid excessive variability.

As a control, the same ribosome profiling protocol and metagene analysis was applied to the *E. coli* sample. In this case, however, no clear peak in the metagene profiles could be detected, probably due to the use of chloramphenicol instead of tetracycline. We used the known translation stalling motif IVDHRP* at the C‐terminal of the *tnaC* leader gene to calibrate the position of ribosomes. The stalling motif is expected to produce a peak of stalled ribosomes with their P‐site positioned at the last proline codon (Seidelt *et al*, 2009). A peak in the coverage of footprints 3′ ends was observed at a distance of 17 nt from the first nucleotide of the proline codon (Appendix Fig S19), similar to the 15 nt found in previous studies (Hwang & Buskirk, 2017). Moreover, this 2 nt increment in the distance from the P‐site to the 3′ end was consistent with the typical length of footprints being ∼3 nt longer compared to other studies.

#### Periodicity in ribosome profiling signal

One of the hallmarks of the ribosome profiling signal has been the three nucleotides periodicity, as observed in yeast, where more than 75% of the reads align to the first nucleotide of codons (Mangkalaphiban *et al*, 2021), reflecting the elongation dynamics of the ribosome. In *E. coli*, however, the signal periodicity is much weaker, with approximately 40% of reads aligning to the first frame (Hwang & Buskirk, 2017). This weak periodicity has been attributed to the sequence specificity of the MNase, which cleaves preferentially before A and T residues (Dingwall *et al*, 1981). Because A and T are found more frequently at the second nucleotide of codons in the *E. coli* genome, most footprints have their 3′ end aligned to the first sub‐codon position (Hwang & Buskirk, 2017). We observed the same nucleotide enrichment in *Mpn*, where the second sub‐codon position showed a higher fraction of A and T compared to the first and third (Fisher exact test two‐sided, *P* ≅ 0 in both cases) sub‐codon positions (Appendix Fig S20). After applying a fixed offset of 19 nt from the 3′ end of footprints of length 20–34 nt, the P‐site of the ribosome occurred more frequently at the third sub‐codon position (median occurrence for the different footprint lengths: 26.4% in frame 1, 31.7% in frame 2 and 40.1% in frame 3) (Appendix Fig S21). These results suggested that the periodicity in the ribosome signal in *Mpn* was mainly driven by the aforementioned biases.

#### *E. coli* cell culture and treatments

*Escherichia coli* DH5ɑ cells were grown overnight in 3 ml of LB medium at 37°C, diluted in 500 ml of LB and grown to an OD_600_ of 0.45 (exponential phase). Cells were treated during 1 min with chloramphenicol at 100 μg/ml final concentration in order to stop translation. After treatment cells were centrifuged at 3,620 *g* and 4°C during 10 min, supernatant was discarded and cell pellet was resuspended in 1.5 ml of lysis buffer supplemented with 1 mg/ml of lysozyme, once the pellet was resuspended, lysis pressure was applied using a prechilled 4639 Cell Disruption Vessel. The cell lysate was centrifuged in a cooled desktop centrifuge at 18,000 *g* for 10 min and the supernatant was frozen into liquid nitrogen for further procedures. The same protocol for ribosome profiling and total RNA‐seq was followed as described above as for *Mpn* cells.

#### Ribosome occupancy

Footprint sizes from 18 to 40 nt (included) were selected and the P‐site shifts applied separately to each footprint size. After applying the P‐site shift, a footprint produced one ribosome count at a single nucleotide position corresponding to the first nucleotide of its P‐site. Ribosome occupancy at codon‐level was computed by summing the ribosome counts at the three sub‐codon positions. The resulting codon‐level ribosome occupancy profile was used for all downstream analyses.

#### Ribosome density

The ribosome and mRNA per base coverages were normalized by the library size factors computed by DEseq2. Ribosome density at the gene level was calculated as the ratio between the average normalized ribosome coverage in the CDS, excluding the first 15 and the last 15 codons, to the average normalized RNA coverage in the CDS. Ribosome density at the codon level was similarly computed as the ratio between normalized ribosome occupancy of codon to the average normalized RNA coverage in the CDS. Thus, local variations in RNA levels were not taken into account to compute ribosome density at codon level.

#### Metafootprint profiles

Ribosome density was normalized by the average density within each CDS, in order to correct for differences in translation initiation rates. The resulting *relative* ribosome density should mainly reflect the local variations in elongation rates. For each codon position along a CDS, local codon context was recorded in a window of 30 codons upstream and 30 codons downstream the ribosome P‐site (position zero). Then, for each codon position and identity, the relative ribosome density at P‐site was averaged over all similar sites in the transcriptome. The stop codons TAA and TAG (in *Mpn* TGA codon encodes for Trp), which could introduce artifacts in the analysis, were excluded. The first 15 and last 15 codons of each CDS were excluded from the analysis, to avoid the atypical accumulation of ribosome footprints observed at the 5′ and 3′ ends of genes. For positions far away from the ribosome decoding center, codon identity had a negligible impact on translation elongation rate, and the measured relative ribosome density averaged over many sites was close to unity. The Jensen‐Shannon divergence was used to compute the divergence of the distribution of average ribosome densities at a specific position, compared to a uniform distribution (density of one for all codon identities). A large divergence indicates a low entropy and a strong influence of codon identity on ribosome density.

#### Strength of the ribosome binding site

Strength of the RBS was predicted by computing the hybridization free energy between the mRNA and the anti‐Shine‐Dalgarno (aSD) motif CCUCCU using the cofold function of the ViennaRNA v2.4.14 (Lorenz *et al*, 2011) with default parameters. In order to correctly position the ribosome for translation initiation, the RBS needs to be positioned in a certain range of distances from the start codon. In bacteria, the most frequent SD motif spacer length is 5–10 nt (Omotajo *et al*, 2015). However, in *M. pneumoniae*, as described in *Escherichia coli*, we often find poly‐A stretches at position +4 of the SD motif that increase the length of the spacer. For example, the highly expressed gene MPN171, encoding for ribosomal protein S3, contains a AAAA stretch, increasing the spacer length to 14 nt. Thus, we increase the allowed spacer length up to 15 nt. The free energy of hybridization was computed for every hexamer at each position allowed in the spacer range, and minimum energy of all positions was assigned as the final affinity for the aSD motif. The canonical Shine‐Dalgarno motif was used because this core sequence is nearly universally conserved in prokaryotes (Omotajo *et al*, 2015) and is also found at the 3′ tail of the 16 s rRNA in *M. pneumoniae*. Genes with a minimum free energy of hybridization lower than −4.8 kcal/mol were considered as possessing a valid RBS. This threshold of energy corresponded to sequences containing at least the AGG partial core motif in general (Ma *et al*, 2002). Presence of SD motif was also searched for at longer ranges in a window of 60 nt upstream the start codon, following the same method.

The presence of internal SD‐like motifs within the CDSs were identified following a similar method. The hybridization free energy between the aSD motif and the mRNA sequence was computed for a sliding window of size 6 along the whole genome. Applying the same threshold as above, windows containing a SD‐like motif were identified. In the presence of a strong SD‐like motif, several consecutives windows with partial overlap with the motif can yield energy values below the threshold. In order to remove duplicated windows corresponding to the same motif position, we applied a mean shift one‐dimensional clustering algorithm (Comaniciu & Meer, 2002) (from python package sklearn v0.23.2; Pedregosa *et al*, 2011), using the length of the motif for the bandwidth parameter, such that two consecutives motifs separated by 0 bp would be recognized as independent motifs. For each cluster, the window with the minimum free energy was chosen. For each CDS, the number of SD‐like motifs within the CDS was counted, as well as the sum of the free energies.

#### Gene annotation

In *Mpn*, some features such as CDSs overlap with other features. In order to simplify the analysis, a list of non‐overlapping features was built in the following way. First, features were ordered by essentiality, and then by size, such that essential and long features were at the top of the list. Features from the ordered list were iteratively added to the non‐overlapping list by allowing a maximum mutual overlap fraction of 10% of the features lengths, such that essential and long features were prioritized. The loop of iterations was repeated, usually twice, until no more features could be added to the non‐overlapping list. The resulting list contained 698 CDSs, with 8 CDSs excluded: MPN029, MPN074, MPN092, MPN150, MPN169, MPN305, MPN587, MPN594. We further excluded 35 pseudogenes, resulting in a final list of 663 CDSs.

Concerning ncRNAs, a list of ncRNAs was selected that did not overlap with any annotated CDS, resulting in a list of 151 ncRNAs.

#### Transcript annotation

The transcriptional landscape in *Mpn* has been shown to be both complex and condition dependent (Junier *et al*, 2016). In particular, the presence of alternative transcription start sites (TSS) and pervasive transcriptional read‐through can give rise to multiple initiation and termination points for transcription, leading to a collection of mRNAs of different lengths, within and across adjacent operons. For the purpose of this study, we simplified the annotation of transcripts by assuming a linear non‐overlapping organization of mRNAs. For each operon, one unique canonical TSS (Yus *et al*, 2012; Junier *et al*, 2016) and one transcription termination site (TTS) were selected, such that a gene can only be in one mRNA, and a TSS can only be associated to one mRNA.

#### Ribosomes copy number

The copy number of assembled ribosome complexes in the cell was estimated at 300, based on previous experimental measurements by cryo‐electron tomography: 140 copies (Kühner *et al*, 2009), 300 copies (Seybert *et al*, 2006; Maier *et al*, 2011), and 300–500 copies (O'Reilly *et al*, 2020).

#### mRNA abundances

RNA‐seq dataset from a previous study of wild‐type *Mpn* at 6 h of growth were used to estimate mRNA abundances (Dataset EV5) (Junier *et al*, 2016). The conversion from RNA‐seq read counts to RNA copy number was based on the estimation of the number of ribosome complexes per cell. Read counts were normalized as transcript per million (TPM). We assumed that 90% of the rRNA was bound to the ribosome and that the read count of the 23S rRNA was a good estimate of the relative number of molecules of rRNA per cell. Assuming a linear relationship between TPM and copy number,

$$\text{mRNA}\_\text{copy}=\text{mRNA}\_\mathrm{TPM}\times \left(\frac{\left(\text{Ribosome}\_\text{copy}\times 0.9\right)}{\text{rRNA}\_23S\_\mathrm{TPM}}\right).$$

The average conversion coefficient between TPM and copy number was 1.06 × 10^−4^.

#### Absolute quantification of protein abundances (AQUA): protein sample preparation

Thirty gram of each sample was reduced with dithiothreitol (2.3 M, 30 min, 56°C), alkylated in the dark with iodoacetamide (5 M mM, 30 min, 25°C) and digested with 3 g LysC (Wako, cat # 129‐02541) overnight at 37°C and then with 3 g of trypsin (Promega, cat # V5113) for 8 h at 37°C following Wiśniewski *et al* (2009) fasp procedure.

#### Absolute quantification of protein abundances (AQUA): chromatographic and mass spectrometric analysis

Seventy‐seven proteins were selected for quantification using selected reaction monitoring or SRM (Dataset EV2). The selected proteins covered more than a 1,000‐fold range of protein concentrations. SRM assays for the selected proteins were developed following the general high‐throughput strategy previously reported (Picotti *et al*, 2009). Unique tryptic peptides ranging from 6 to 20 amino acids in length and previously observed in discovery MS experiments (Martens *et al*, 2005; Desiere *et al*, 2006) were prioritized during the peptide selection process for each protein (Dataset EV2). All peptides containing amino acids prone to undergo unspecific reactions (e.g. Met, Trp, Asn, and Gln) were generally avoided and only selected when no other options were available (Lange *et al*, 2008). The selected peptides were chemically synthesized using C‐terminal 13C6, 15N2‐Lysine or 13C6, 15N4‐Arginine (Thermo Fisher Scientific, Germany) and used for SRM assay development. Fragment ion spectra were collected for each peptide using a shotgun data‐dependent acquisition method in a LTQ‐Orbitrap Velos Pro mass spectrometer (Thermo Fisher Scientific, San Jose, USA) coupled to a nano‐flow HPLC (EasyLC Proxeon, Odense, Denmark).

The obtained MS2 data were analyzed with Proteome Discoverer software suite (v1.4, Thermo Fisher Scientific, Germany) and the Mascot search engine (v2.5, Matrix Science; Perkins *et al*, 1999). Search parameters were set to 7 ppm for the precursor mass tolerance, 0.5 Da for the fragment mass tolerance, and fully tryptic peptides and no missed cleavages were selected. An identification false discovery rate (FDR) of 1% based on decoy assignments was used (Elias & Gygi, 2007). Oxidation of methionine and protein acetylation at the N‐terminal were defined as variable modification, whereas carbamidomethylation on cysteines, and 13C6,15N2‐Lysine and 13C6,15N4‐Arginine were set as fixed modifications.

SRM measurements were performed on a 5500 Q‐Trap mass spectrometer (AB Sciex, Framingham, MA, USA) coupled to a nanoLC Ultra‐1DPlus (AB Sciex). Briefly, peptide mixtures were loaded onto a C18 Acclaim PepMap precolumn (Thermo Fisher Scientific, cat # 164564) and were separated by reversed‐phase chromatography using a 25‐cm column with an inner diameter of 75 μm, and packed with 1.9 μm C18 particles (Nikkyo Technos Co, Japan). The chromatographic gradient started at 92.8% buffer A (0.1% formic acid in water) and 7.2% buffer B (0.1% formic acid in acetonitrile) with a flow rate of 250 nl/min for 5 min and gradually increased to 60% buffer A and 40% buffer B in 60 min. After each analysis, both the pre‐column and the column were washed for 10 min with 2% buffer A and 98% buffer B. Digested beta‐galactosidase was analyzed between the SRM measurements of biological samples to avoid sample carryover and to assure stability of the instrument.

Measurements were done in scheduled SRM mode, using a retention time window of 6 min. Around 250 transitions were packed per method with a maximum total cycle time of 1.5 s, to ensure dwell times over 10 ms per transition. For each peptide, at least 3 transitions were monitored (Dataset EV3). SRM data was processed using the Skyline software v3.1.0.7382 (MacLean *et al*, 2010) and data peaks were evaluated based on retention time, transition intensity rank as compared with MS2 spectral library, co‐elution between the endogenous and the labeled reference peptide were also considered.

To determine the linear range of the peptides, different amounts of each peptide were injected per triplicate and a linear regression was generated for each peptide.

Three time points of the *Mycoplasma pneumoniae* growth curve (48, 72 and 96 h) and two amounts (500 and 2,000 ng) plus the heavy peptides (Dataset EV2) were injected per triplicates in order to calculate the endogenous amount of each peptide (single reference point quantitation) (Campbell *et al*, 2011) (Dataset EV4).

Two thousand nanogram of three time point of the *M. pneumoniae* growing curve (48, 72 and 96 h) were analyzed using a LTQ‐Orbitrap Velos Pro mass spectrometer (Thermo Fisher Scientific, San Jose, CA, USA) coupled to an EasyLC (Thermo Fisher Scientific (Proxeon), Odense, Denmark). Peptides were loaded onto the 2‐cm Nano Trap column with an inner diameter of 100 μm packed with C18 particles of 5 μm particle size (Thermo Fisher Scientific) and were separated by reversed‐phase chromatography using a 25‐cm column with an inner diameter of 75 μm, packed with 1.9 μm C18 particles (Nikkyo Technos Co., Ltd. Japan). Chromatographic gradients started at 93% buffer A and 7% buffer B with a flow rate of 250 nl/min for 5 min and gradually increased 65% buffer A and 35% buffer B in 120 min. After each analysis, the column was washed for 15 min with 10% buffer A and 90% buffer B. Buffer A: 0.1% formic acid in water. Buffer B: 0.1% formic acid in acetonitrile.

The mass spectrometer was operated in DDA mode, and full MS scans with 1 micro scans at resolution of 60,000 were used over a mass range of *m/z* 350–2,000 with detection in the Orbitrap. Auto gain control (AGC) was set to 10^6^, dynamic exclusion (60 s) and charge state filtering disqualifying singly charged peptides was activated. In each cycle of DDA analysis, following each survey scan the top twenty most intense ions with multiple charged ions above a threshold ion count of 5,000 were selected for fragmentation at normalized collision energy of 35%. Fragment ion spectra produced via collision‐induced dissociation (CID) were acquired in the Ion Trap, AGC was set to 5 × 10^4^, isolation window of 2.0 *m/z*, activation time of 0.1 ms and maximum injection time of 100 ms was used.

Proteome Discoverer software suite (v2.0, Thermo Fisher Scientific) and the Mascot search engine (v2.5, Matrix Science) (Perkins *et al*, 1999) were used for peptide identification. Samples were searched against a *M. pneumoniae* database with a list of common contaminants and all the corresponding decoy entries (87,059 entries). Trypsin was chosen as an enzyme and a maximum of three miscleavages were allowed. Carbamidomethylation (C) was set as a fixed modification, whereas oxidation (M) and acetylation (N‐terminal) were used as variable modifications. Searches were performed using a peptide tolerance of 7 ppm, a product ion tolerance of 0.5 Da. Resulting data files were filtered for FDR < 5%. Protein top 3 areas have been calculated with unique peptides per protein ungrouped.

#### Calibration of protein absolute abundances versus TOP3

Protein top3 intensities and absolute amount of peptides were not normalized, in order not to introduce any bias into the calibration. A robust linear regression was performed on the absolute amount of each peptide of the SRM assay (P_abs_) and the protein top 3 intensity of the Orbitrap (P_TOP3_), following the equation

$$\log_{2}\left(P_{\mathrm{TOP}3}\right)=\alpha +\beta \times \log_{2}\left(P_{\mathrm{abs}}\right),$$

using all peptide values for all replicates and the three growth time points, assuming that the proteome composition does not change significantly after 48 h. The protein top3 intensity was considered as the dependent variable because robust linear regression methods perform better with large variations in the *y*‐axis rather than the *x*‐axis. The presence of outliers led to an under‐estimation of the absolute abundance for lowly abundant proteins (Appendix Fig S22). In order to reduce the effect of outliers, the RANSAC (RANdom SAmple Consensus) iterative algorithm was used (scikit‐learn package v0.23.2) (Fischler & Bolles, 1981). This method fits a model from random subsets of inliers from the complete data set. A maximum of 10000 iterations were performed, resulting in 163 values out of a total of 537 identified as outliers. The resulting robust linear regression better fitted the data in the low abundance range and produced a slope closer to linearity (beta coefficient closer to unity) compared to a simple linear regression with all data points.

α=23.6,β=0.939.

The linear model axes were rotated such as to predict the absolute protein abundance in fmol from the normalized TOP3 protein intensity for all samples.

#### Normalization of protein top3 intensities for external samples

Protein top3 intensities were normalized between samples by using a robust linear regression of the MA‐transformed log_2_ of protein intensities. This normalization method was shown to perform well for estimation of absolute protein abundances from label‐free proteomics (Välikangas *et al*, 2018). Before normalization, the data was transformed into the log_2_ scale, in order to reduce the influence of the variability of protein with high abundances and to obtain a more normal‐like distribution. An artificial reference sample was created by computing the median of top3 intensities for each protein across all 9 samples (Appendix Fig S22). First, a robust linear regression (Huber's T estimator, default tuning constant *t* = 1.345, python statsmodel v0.11.1; Seabold & Perktold, 2010) was performed on the log_2_ protein intensity (abundance A) versus fold change (M) plot, where A and M read

$$M=\log_{2}\left(P_{\text{sample}}\right)-\log_{2}\left(P_{\mathrm{ref}.}\right),$$

$$A=\frac{\left(\log_{2}\left(P_{\text{sample}}\right)+\log_{2}\left(P_{\mathrm{ref}.}\right)\right)}{2}.$$

In this first step, values were corrected by a constant M*, the predicted fold change at average abundance,

$$\log_{2}\left(P_{\text{sample}}^{\prime }\right)=\log_{2}\left(P_{\text{sample}}\right)-M^{*},$$

such that the scale of abundances in the two samples were comparable. In a second step, a second robust linear regression was applied to the MA plot, using as abundances the scaled abundances of the sample alone,

$$A=\log_{2}\left(P_{\text{sample}}^{\prime }\right),$$

$$M^{**}\left(A\right)=\alpha_{\mathrm{MA}}+\beta_{\mathrm{MA}}\times A.$$

The fitted linear regression model of the M** correction was computed based on the scaled abundances of protein in the sample and then subtracted to produce normalized abundances $P_{\text{sample}}^{\prime \prime }$,

$$\log_{2}\left(P_{\text{sample}}^{\prime \prime }\right)=\log_{2}\left(P_{\text{sample}}^{\prime }\right)-M^{**}\left(A\right).$$

This procedure allowed normalization for proteins that were only detected in the external sample and not in the reference.

#### Protein copy numbers

Protein copy numbers were estimated by two methods.

First, as described in (Maier *et al*, 2011), the total protein content (2 μg) and DNA content (2 × 10^8^ cells) were measured along the growth curve, resulting in a protein mass per cell of 10 fg at 48 h of growth. We computed a weighted average protein molecular weight according to individual molecular weights of all proteins and their relative fraction in the proteome. The relative fraction was computed as the fraction of the TOP3 protein intensity in sample 48 h replicate 3 relative to the sum of all intensities, where undetected proteins were assigned a relative fraction of 1 × 10^−7^. The resulting average protein molecular weight was 45,402 Da. Thus, by dividing the protein mass of one cell by the average protein molecular weight, we obtained an approximate total protein copy number of 1.33 × 10^5^ per cell.

Second, the absolute protein abundances determined by the AQUA calibration were used to derive the copy number per cell of each protein individually. Significant differences in the total amount of labeled peptides were observed between samples, probably due to variability in the total protein content injected in the spectrometer. The absolute amount of labeled proteins used in the AQUA calibration were corrected for such differences using the same average fold change correction as in the normalization of the top3 intensities. For proteins with labeled peptides, the corrected absolute abundances derived from the heavy‐to‐light ratios were used instead of the values deduced from the fitted calibration curve. For each protein, the copy number was computed based on its estimated absolute abundance in fmol and the total number of cells in the sample (2 × 10^8^),

$$P_{\text{copy},i}=\frac{P_{\mathrm{abs},i}\times {\mathrm{Mw}}_{i}\times N_{A}\times 10^{-15}}{N_{\text{cells}}},$$

where P_abs,i_ is the absolute abundance of protein i in fmol, Mw_i_ its molecular weight in Da, N_A_ the Avogadro number, N_cells_ the number of cells in the sample, and P_copy,i_ the copy number of protein i. The total number of proteins per cell obtained was ranging between 1.153 × 10^5^ and 1.492 × 10^5^ depending on the sample, with an average over all samples of 1.292 × 10^5^.

#### Average protein abundances

The absolute quantification of the three samples at 48 h of growth were chosen as reference for the absolute protein copy numbers. In addition, four additional proteomic datasets of *Mpn* cells at 48 h of growth were used (Miravet‐Verde *et al*, 2019): two samples using SDS‐PAGE gel‐based protein extraction method (160908_S_TFLS_21_02_2ug and 160908_S_TFLS_21_03_2ug), and two samples using urea as gel‐free protein extraction method (161116_S_TFLS_10_01_2ug and 161116_S_TFLS_10_02_2ug). We observed that the total signal (sum of normalized protein top3 intensities) of predicted membrane proteins (Burgos *et al*, 2020) was higher for samples using SDS gel‐based extraction method, while the signal for proteins smaller than 180 residues was higher for samples using gel‐free urea‐based extraction. Moreover, seven additional proteins could be detected in the gel‐free samples compared to the reference samples, and four additional proteins in the gel‐based samples. Protein intensities for these four external samples were normalized with respect to the reference median sample, and absolute abundances and copy numbers were computed based on the calibration curve. The average total protein copy number was slightly higher in the gel‐base samples (1.542 × 10^5^) and in the gel‐free samples (1.472 × 10^5^) compared to the reference samples (1.215 × 10^5^).

We evaluated the precision of the estimation of proteome‐wide absolute protein abundances by analyzing the distances between log_2_ copy numbers estimated from the external samples and log_2_ absolute copy numbers derived from the median absolute abundances of the labeled peptides (Appendix Figs S22 and S23A–D). We found that the distances were distributed around zero, with an average standard deviation of 1.49 log_2_ and an average absolute distance of 1.12 log_2_. However, for some labeled peptides, the estimated copy number differed up to plus or minus 4 log_2_. Thus, the average fold change error in the estimation of protein copy numbers from mass spectrometry was approximately ±1.5 log_2_. Importantly, these deviations did not improve when averaging the estimated copy numbers over all the external samples, showing that they were mostly due to intrinsic differences in the efficiency of peptide ionization and flight characteristics in the mass spectrometer (Steen & Pandey, 2002).

The final protein copy numbers (Dataset EV9) were computed as the weighted average across the three replicates at 48 h of the AQUA quantification and the additional four samples, where more weight was given to the urea samples for proteins whose length was smaller than 400 residues, with a maximum weight of 7. The copy numbers derived from the absolute abundances of labeled peptides were chosen instead of the estimated copy numbers when available (66 proteins). In addition, the copy numbers for 7 proteins with labeled peptides that were detected in the other time points (72 and 96 h) but not in the 48 h time point were chosen. In total, 528 proteins were quantified and the final total amount of protein copy number per cell was 1.207 × 10^5^.

#### Modeling approach: absolute translation efficiency derived from omics data

We consider a simple model of protein synthesis and degradation, assuming that mRNA levels (M) are constant, that the protein synthesis from the mRNA can be considered as a single step reaction with rate $k_{\mathrm{TE}}^{\mathrm{abs}}$ (translation efficiency), and that the protein degradation rate (*λ*_*P*_) is constant. At steady‐state, the kinetic equation for protein abundance (*P*) reads

$$\frac{\mathrm{dP}}{\mathrm{dt}}=k_{\mathrm{TE}}^{\mathrm{abs}}M-\lambda_{P}P,$$

such that

$$k_{\mathrm{TE}}^{\mathrm{abs}}=\frac{\lambda_{P}P}{M}.$$

In the framework of the initiation‐limited ribosome flow model, the translation efficiency $k_{\mathrm{TE}}^{\mathrm{abs}}$ is equal to the initiation rate *k*_*ini*_. We defined the local elongation rate *k*_*elong,i*_ and the local ribosome density *ρ*_*i*_ at codon *i*. We assumed no ribosome drop‐off during elongation, such that the flow of ribosomes is conserved along the transcript. In this context, the flow of ribosomes at every codon position is simply equal to the translation rate, i.e. $k_{\mathrm{TE}}^{\mathrm{abs}}$ = *k*_*ini*_ = *k*_*elong,i*_ *ρ*_*i*_. Then, we integrated the absolute translation efficiency $k_{\mathrm{TE}}^{\mathrm{abs}}$ in our modeling approach, together with ribosome profiling data, to derive translation initiation and translation elongation rates, both at the gene level and at the codon level.

#### Modeling approach: scaling factors

Two scaling factors need to be defined in order to relate experimental ribosome density to the absolute physical units, one for ribosome density and one for translation rates.

Ribosome profiling data only provides relative values for the density of ribosomes on mRNAs. We introduce a scaling factor, *r*_1_, such that

$$\rho_{i}=r_{1}\nu_{i},$$

where *ρ*_*i*_ is the number of ribosomes per mRNA bound to codon position *i*, and *ν*_*i*_ is the normalized experimental ribosome density at codon position *i*. Following a procedure similar to previous studies (Siwiak & Zielenkiewicz, 2010; Gritsenko *et al*, 2015), we calculated the value of *r*_*1*_ from the number of actively translating ribosomes and the number of mRNA molecules per cell. The fraction of ribosome actively translating was assumed to be similar to the one in *E. coli* (Dai *et al*, 2016), *f*_*active*_ = 0.8. The size of the expressed coding transcriptome, i.e. the number of codons of all mRNA molecules present in the cell at a given time, was computed as

$$N_{c}^{\text{trans}}=\sum_{g}l_{g}M_{g}=13996\;\text{codons},$$

where *l*_*g*_ and *M*_*g*_ are respectively the length of the coding sequence in codons and the absolute number of transcripts of gene *g*, as measured experimentally. Thus, the average ribosome density in the cell can be expressed as,

$${\left\langle \rho \right\rangle }_{\text{trans}}=\frac{N_{\text{ribo}}^{\mathrm{tot}}f_{\text{active}}}{N_{c}^{\text{trans}}}=1.71\cdot 10^{-2}\;\text{ribosome}\;{\text{codon}}^{-1},$$

or one ribosome every 58 codons on average.

Note that we differentiated the average over the expressed transcriptome ${\left\langle \right\rangle }_{\text{trans}}$, where position‐specific observables are counted as many times as transcript copies, to the average over the coding genome ${\left\langle \right\rangle }_{\text{genome}}$, where position‐specific observables are counted only once per gene.

We normalized further the ribosome density *ν*_*i*_ by its average over the expressed transcriptome and used the average absolute ribosome density as a scaling factor, such that the ribosome density in absolute units reads,

$$\rho_{i}=\frac{r_{1}}{{\left\langle \nu_{i}\right\rangle }_{\text{trans}}\nu_{i}},$$

where

$${\left\langle \nu_{i}\right\rangle }_{\text{trans}}=\frac{\sum_{i}M_{i,g}\nu_{i}}{\sum_{i}M_{i,g}},$$

with *M*_*i,g*_ is the number of transcript molecules at codon *i* and gene *g*.

The second scaling factor that we introduced is the absolute translation efficiency. We measured protein abundance in copies per cell, mRNA in copies per cell, and protein degradation rate in second^−1^, and used these quantities to derive the average codon elongation rates.

#### Modeling approach: codon elongation rates

In this analysis, we assumed that the elongation rates are only dependent on the identity of the codon at the A‐site and are constant across genes. For each individual gene, $k_{\mathrm{TE}}^{\mathrm{abs}}$ was used to determine absolute elongation rates at each codon position from the absolute ribosome density, *k*_*elong,i*_ = *k* ^*abs*^_*TE*_/*ρ*_*i*_. Ribosome profiling data is very sparse, with 50–70% of codon positions with zero ribosome count. The local elongation rate at positions with zero ribosome counts is not well defined. One possibility would be to choose a maximum elongation rate for those positions (Dao Duc & Song, 2018). However, given the high proportion of such positions, the final average elongation rate would depend strongly on the arbitrary maximum elongation rate. We averaged the *time* spent by a ribosome at each codon along a coding sequence, defined as the inverse of the elongation rate,

$$\mu_{i}=\frac{1}{k_{\text{elong},i}}=\frac{\rho_{i}}{k_{\mathrm{TE}}^{\mathrm{abs}}},$$

where *μ*_i_ is the ribosome dwell time at codon *i*. Computing the average of the dwell times is equivalent to taking the harmonic mean of the elongation rates. We averaged the dwell times for all the sites of codon identity *c* for all genes over the expressed transcriptome, i.e. taking into account the level of expression of each transcript,

$${\left\langle \mu_{c}\right\rangle }_{\text{trans}}=\frac{\sum_{g}M_{g}\sum_{i|c_{i}=c}^{N_{c,g}}\frac{\rho_{i}}{k_{\mathrm{TE}}^{\mathrm{abs}}}}{\sum_{g}M_{g}N_{c,g}},$$

where *N*_*c,g*_ denotes the number of codons with identity *c* in gene *g*. In order to reduce the impact of biases in the ribosome density close to the initiation and termination sites, the first and last 15 codons of each gene were excluded from the analysis. The average elongation rate is then

$${\left\langle k_{\text{elong},i}\right\rangle }_{\text{trans}}=\frac{1}{{\left\langle \mu_{c}\right\rangle }_{\text{trans}}}.$$

Elongation rates for the stop codons TAA and TAG were excluded for all further analyses.

#### Codon usage

Codon usage was computed for all genes, and codon adaptation index (CAI) was computed based on the set of highly abundant proteins, defined by the top 20% of protein abundances, using a custom python script.

#### Secondary structure prediction

Secondary structure at the initiation region of genes was computed using ViennaRNA v2.4.14 (Lorenz *et al*, 2011) with default parameters, in a region of 40 nt upstream to 40 nt downstream the first nucleotide of the start codon.

#### Variations in local elongation rate

We computed the local variation of the elongation rate at position *i*, $k_{\text{elong},i}^{\mathrm{rel}}$ = (1/*ρ*_*i*_)/<*k*_*elong,i*_>_*gene*_, derived from the ribosome profiling data, *k*_*elong,i*_ = *1*/*ρ*_*i*_, and normalized by the average of each gene. Because the local variation was normalized within each gene, it did not depend on the absolute values of the translation efficiency and could be computed for all the genes with ribosome profiling data (99% of all genes). Codon positions with zero ribosome counts were filtered out (56%), since they could not inform on the elongation rate. In addition, the first 30 and last 30 codons of each CDS were filtered out, in order to reduce the influence of mechanisms specific to translation initiation and termination. Positions for which a stop codon was found in the window [−30, +30] codons were filtered out. The final selected 87,419 codon positions represented 38% of the coding transcriptome.

The strength of internal SD‐like motifs was computed by calculating the hybridization energy to the aSD sequence (see section about internal SD‐like motifs), in a 6 nt window at each base pair of the genome. Then, the free energy was aggregated at the codon‐level for all CDSs by choosing the minimum free energy within the 3 subcodon positions.

The full list of numerical features was: (i) SD‐like motif free energy in 6 nt window with left boundary positioned at −24, −18, −12, −6, 0, +6, +12, +18, +24 nt from the P‐site codon, taking the minimum of free energy on the 3 subcodons positions, (ii) fraction of positively charged residues (Lys, Arg, His), negatively charged residues (Glu, Asp), polar residues (Asn, Gln, Ser, Thr), hydrophobic residues (Ala, Ile, Leu, Met, Val, Phe, Trp, Tyr), average hydrophobic score of amino acids (Kyte‐Doolittle hydrophobicity scores), in a 30 nt window with left boundary positioned at −30, −15, and +1 nt from the P‐site codon, (iii) presence of di‐amino acid repeats (Lys‐Lys, Ser‐Ser, etc) and tri‐amino acid repeats (Lys‐Lys‐Lys, Ser‐Ser‐Ser, etc) in a 3 codons window with left boundary at position −3, −1 and +1, (iv) mRNA folding energy in a window of 40 nt with left boundary at position −80, −70, −60, −50, −40, −30, −20, 10, 0, +10, +20, +30 and +40 nt from the codon P‐site, and (v) CDS length in nt.

The features with the strongest positive Pearson correlation (Appendix Fig S8) with the local variation $k_{\text{elong},i}^{\mathrm{rel}}$ were the free energy of hybridization of SD‐like motifs (*r* = 0.11) at various positions from −6 to 18 nt, meaning that stronger SD‐like motifs (more negative free energy) was associated to slower local elongation rate, the fraction of polar residues (*r* = 0.07) in the window at position [−15, 14] nt, the relative position along the CDS (*r* = 0.05), and the mRNA folding free energy (*r* = 0.04) in window [10, 49] nt, meaning that stronger mRNA secondary structure downstream the ribosome was associated to slower local elongation rate. The strongest negative correlations were found for the fraction of negatively charged residues (*r* = −0.07) and the fraction of special residues (*r* = −0.03) in the window [−15, 14] nt. In comparison, we also computed the correlation of the local variation $k_{\text{elong},i}^{\mathrm{rel}}$ with the average codon elongation rates derived from the average over all genes. The resulting correlation, *r* = 0.10, indicated that most of the variation in local elongation rate cannot be explained by the decoding rate of the A‐site codon alone. The same analysis using Spearman correlation coefficient produced equivalent results. All described correlation coefficients were significant with *P* < 10^−27^, due to the large number of codon sites analyzed (87419).

We included as additional categorical features the codon identity at positions −30, −3, −2, −1 (A‐site), 0 (P‐site), +1, +2, +3, +4, +5, +6, and +30. The codons at positions −3 to +6, including the A‐site codon, were the positions with the highest divergence (influence) in the metafootprint analysis. In order to improve the performance of the regressor, codons were ordered by the average elongation rate at the A‐site. Although introducing some bias into the training of the predictor, this pre‐ordering was necessary to optimize the training of the base decision trees. Indeed, the performance score when using a shuffled list of codons decreased to 5.6%, due to the small differences between codons. In addition, we randomized the identity of the A‐site codon and included it as a negative control feature, which should have no influence on the local elongation rate.

Pearson and Spearman correlations were calculated with the scipy v1.7.3 python package (Virtanen *et al*, 2020), which also computes an approximate *P*‐value for the significance of the correlation.

The local variations of elongation rate $k_{\text{elong},i}^{\mathrm{rel}}$ data was split into a training set (80%) and a test set (20%). Categorical data such as the codon identity at the different positions were transformed into ordinal integers values (from 0 to 63). A random forest regressor (Breiman, 2001) was trained on the training data, using the scikit learn python package v1.0.2 (Pedregosa *et al*, 2011). The performance of the regressor was evaluated by two means: (i) the out‐of‐bag (OOB) score, i.e. the coefficient of determination *R* ^2^ determined from the estimated error from the out‐of‐bag samples during the bootstrap process, and (ii) the test score, i.e. the coefficient of determination *R* ^2^ determined from the error between the predicted values and the target values of the test set. The optimal number of estimators (trees) was determined by computing the scores for an increasing number of trees, showing that ∼100 trees were enough to reach the maximum performance. Using this number of estimators, the maximum depth was determined to saturate at 12. The minimum number of samples per leaf was set to 10. Increasing the minimum number of samples per leaf above 100 greatly impacted the score, showing that a small number of specific positions in the genome were very informative for determining the local elongation rate. The maximum number of features provided to each base estimator was set to all the possible features. The maximum number of samples drawn during the bootstrap procedure was set to 70%.

The relative importance of the features was estimated by three different methods. First, the built‐in method based on the variance reduction at each node averaged over all trees was used. Second, the permutation method of the scikit learn package, using 10 random permutations was used (Appendix Fig S24). In this method, the importance is defined as the difference in the regressor score between its prediction with permuted feature values and its prediction without permutation. Third, the SHAP TreeExplainer method (python package v0.38.0) (Lundberg *et al*, 2020) was applied on all the samples of the test set. This method computes local explanation values for each codon position of the test set (SHAP values). These values reflect the magnitude and direction of the impact on the model output for each feature individually.

#### Hydro‐tRNA‐seq library preparation

Eight to ten micrograms of Total RNA were loaded on a 15% denaturing urea polyacrylamide gel (EC6885BOX, Thermo Scientific) and RNA fragments of 60–100 nt were selected. They were then subjected to limited alkaline hydrolysis, followed by dephosphorylation using Antarctic phosphatase (M0289, New England Biolabs) and rephosphorylation using PNK (M0201, New England Biolabs). Then, 3 prime and 5 prime adapters were ligated to the RNA fragments, and these were subsequently reverse transcribed and amplified by PCR, where different indexed primers were used to allow multiplexed sequencing.

Final libraries were analyzed using Agilent High Sensitivity chip to estimate the quantity and check size distribution and were then quantified by qPCR using the KAPA Library Quantification Kit (ref. KK4835, KapaBiosystems) prior to amplification with Illumina's cBot.

#### Hydro‐tRNA‐seq data processing

Processing of the reads was identical as for the RNA‐seq paired‐end reads processing described above, except for the following parameters: minimum read length after trimming: 10, bowtie2 seed length: 14, maximum length insert size: 200. Fragment counts in tRNA genes were computed using bedtools, with strand specific overlaps with minimum overlap fraction of 0.98 of read length. Thus, reads that could originate from immature tRNAs, with a significant overhang at either the 5′ or at the 3′ of the tRNA coordinates, were excluded.

We reasoned that intact tRNA molecules that were not fragmented by the hydrolysis could maintain their secondary structure and introduce bias in the quantification. However, excluding fragments that corresponded to full‐length tRNAs, and selecting fragment sizes smaller than 40 nt produced very similar relative abundances (Appendix Fig S25), suggesting that the full‐length fragment did not introduce any bias in the quantification.

#### tRNA gene re‐annotation

We predicted tRNA genes and structures using Aragorn v1.2.38 (Laslett & Canback, 2004) with the option “‐t ‐gcprot” using Mycoplasma genetic code, assuming a circular topology of the genome, searching in both strands.

We discarded the predicted tRNA with TTG anticodon at coordinates 134011_134081 as a false positive: its predicted structure seemed less stable (many missing base pairings) and it lacked the CCA tail, usually present in all tRNA genes in *Mpn*.

We retrieved the annotated genome of *Mpn* in GenBank format from the National Center for Biotechnology Information (NCBI) database (http://www.ncbi.nlm.nih.gov), accession U00089.2, and retrieved all tRNA gene annotations. We compared the predicted tRNA genes to the annotation in the NCBI database and detected some discrepancies.

MPNt26 was not predicted by Aragorn. The coverage profile of the hydro‐tRNA‐seq showed a very low per‐base coverage of 8–10 reads. Moreover, the RNA coverage profile showed no increase in RNA level between the 5′ of the transcript (coordinate 558612) and the 5′ of the hypothetical tRNA (coordinate 558,634). In other words, there was no enrichment of reads specific to the tRNA region. The reads mapping to this region, together with the assigned transcription start site (TSS) indicated the presence of a ncRNA, ncMPN448, with approximate coordinates 558612_558737.

MPNt34 was predicted by Aragorn and annotated in NCBI at coordinates 643913_643988, but hydro‐tRNA‐seq coverage showed higher coverage for positions 643912_643988, i.e. with the 5′ end one bp upstream.

The function of the initiator and elongator Met‐tRNAs was confirmed based on the predictions in (Ardell & Hou, 2016).

#### tRNA adaptation index

We used the general wobble base pairing weights proposed by (Seward & Kelly, 2018) which were used to analyze bacterial genomes across all phyla. It has been suggested that the efficiencies of the different codon–tRNA interactions could vary among different organisms and that the base pairing weights can be modified for each species by optimizing the correlation between tAI and codon usage bias (Sabi & Tuller, 2014). However, we failed to optimize the weights for *Mpn* using the stAIcalc tool (Sabi *et al*, 2017).

*Mpn* uses one single tRNA harboring a U34 to read all four codons of the 4‐degenerated codon boxes Ala, Pro and Val. In this case, the U34 is most probably unmodified, in order to allow wobble base pairing with all four codons. In these codon boxes, we used wobble interaction weights that allow U34‐N base pairing. In contrast, for all the split family 2‐codon boxes, the wobble U34 of tRNAs have to discriminate for the NN‐purine codons and not misread the NN‐pyrimidine codons (Grosjean *et al*, 2010). In bacteria, the U34 is often modified to both improve the interaction with the synonymous NNG codon and to restrict wobble interaction with NN‐purine codons and prevent misreading. This is exemplified in the Cys‐Trp split 2‐codon families. In *Mpn*, as in most mollicutes, the UGA codon has been reassigned as Trp by an extra Trp‐tRNA harboring a U*CA anticodon, consistent with the loss of termination factor RF2 in those species (Citti *et al*, 1992; Grosjean *et al*, 2014). This additional Trp‐tRNA has to read the frequently used UGA codon without confusing it with the Cys codons UGU and UGC of the same decoding box (de Crécy‐Lagard *et al*, 2007; Grosjean *et al*, 2010). This is the reason why the U34 in Trp‐tRNA‐U*CA is found hypermodified (cmnm^5^Um) in *Mycoplasma capricolum* (Andachi *et al*, 1989), a related species of the Mollicute class, and most probably also in *Mpn* as it possesses all the necessary modification enzyme genes. This type of hypermodified U34 has been shown to restrict decoding to only purine‐ending codons (Takai & Yokoyama, 2003), which would avoid the misreading of near‐cognate pyrimidine‐ending codons UGU and UGC. For simplicity, we assumed that all tRNAs of split 2‐codon boxes harboring a U34 were modified such that the U34‐U and U34‐C wobble base pairings were forbidden.

As other bacteria, *Mpn* possesses the TilS enzyme that catalyzes the modification of the wobble C34 of the mature Ile‐tRNA‐CAU to lysidine L34, which allows the correct recognition of Ile‐AUA codon without confusing it with the Met‐AUG codon.

The weights were

$$s_{I:U}=0,s_{I:C}=0.28,s_{I:A}=0.99,$$

$$s_{U:G}=0.68,s_{U:A}=0,$$

$$s_{C:G}=0,$$

$$s_{L:A}=0.89.$$

In addition, as mentioned in the main text, we allowed four‐way wobbling for U34 for tRNAs of the 4‐codon boxes Val, Pro, and Ala, with weights,

$$s_{U:U}=0.7,s_{U:C}=0.95.$$

#### Aminoacyl‐tRNA synthetases

Levels of aminoacyl‐tRNA synthetases (aaRSs) were determined by our absolute quantification of protein abundances approach. *Mpn*, like other mollicutes, only possesses 19 aaRSs and lacks the gene coding for glutaminyl‐tRNA synthetase (GlnS) found in other bacteria. Instead, it encodes for a non‐discriminating type of glutamyl‐tRNA synthetase (GltX) that charges both tRNA^Glu^ and tRNA^Gln^ with Glu (Grosjean *et al*, 2014) and a heterotrimeric enzyme (Gln‐tRNA amidotransferase complex) that amidates Glu‐tRNA^Gln^ to Gln‐tRNA^Gln^ (O'Donoghue *et al*, 2011). For Gln, we considered the mean between the abundances of the amidotransferase and the Glu aaRS, and compared it to the sum of the a.a. and tRNA abundances for both Glu and Gln. For the heterodimeric phenylalanyl‐tRNA synthetase, we computed the mean copy number of the two subunits.

#### Gene‐averaged elongation rate, simulated noise

We simulated two types of noise in $k_{\mathrm{TE}}^{\mathrm{abs}}$, (i) gaussian noise with a fixed standard deviation, and (ii) gaussian noise with a variable standard deviation. Simulated negative values were clipped to the minimum experimental value of $k_{\mathrm{TE}}^{\mathrm{abs}}$ (6 × 10^−4^ s^−1^). When simulating added gaussian noise with a fixed standard deviation equal to 10 times the median $k_{\mathrm{TE}}^{\mathrm{abs}}$, the Pearson correlation between $k_{\mathrm{TE}}^{\mathrm{abs}}$ and $k_{\mathrm{TE}}^{\mathrm{abs}}$/<*ρ*> remained moderate (Appendix Fig S12B), *r* ≈ 0.4 for unlabeled proteins and *r* ≈ 0.4 for labeled proteins. When simulating noise with a standard deviation equal to 2 times the value of $k_{\mathrm{TE}}^{\mathrm{abs}}$, i.e. with CV = 2, the Pearson correlation between the two quantities was higher (Appendix Fig S12D), *r* ≈ 0.6 for unlabeled proteins and *r* ≈ 0.6 for labeled proteins. Increasing even more the intensity of noise led to decreasing correlation coefficients.

#### Bioinformatics analysis tools

Intersection of gene sets was performed using the upsetplot python package v0.6.1 (Lex *et al*, 2014).

## Author contributions

**Marc Weber:** Conceptualization; data curation; software; formal analysis; investigation; visualization; methodology; writing – original draft; writing – review and editing. **Adrià Sogues:** Conceptualization; validation; investigation; methodology; writing – review and editing. **Eva Yus:** Conceptualization; investigation; methodology; writing – review and editing. **Raul Burgos:** Conceptualization; data curation; validation; investigation; methodology; writing – review and editing. **Carolina Gallo:** Data curation; investigation; methodology; writing – review and editing. **Sira Martínez:** Data curation; validation; investigation; methodology; writing – review and editing. **Maria Lluch‐Senar:** Conceptualization; resources; supervision; investigation; methodology; project administration; writing – review and editing. **Luis Serrano:** Conceptualization; resources; formal analysis; supervision; funding acquisition; investigation; methodology; writing – original draft; project administration; writing – review and editing.

## Disclosure and competing interests statement

The authors declare that they have no conflict of interest. LS is member of the Editorial Advisory Board of Molecular Systems Biology. This has no bearing on the editorial consideration of this article for publication.

## Supporting information



Appendix

Dataset EV1

Dataset EV2

Dataset EV3

Dataset EV4

Dataset EV5

Dataset EV6

Dataset EV7

Dataset EV8

Dataset EV9

## Data Availability

The datasets produced in this study are available in the following databases: (i) tRNAs quantification by hydro‐tRNA‐seq: ArrayExpress E‐MTAB‐11930 (https://www.ebi.ac.uk/biostudies/arrayexpress/studies/E‐MTAB‐11930); (ii) Ribosome profiling RNA‐seq of *Mpn* and *E. coli*: ArrayExpress E‐MTAB‐11935 (https://www.ebi.ac.uk/biostudies/arrayexpress/studies/E‐MTAB‐11935); (iii) Absolute protein quantification by mass spectrometry: PRIDE PXD035159 (https://www.ebi.ac.uk/pride/archive/projects/PXD035159).
