# Supplementary material for: Comprehensive quantitative modeling of translation efficiency in a genome‐reduced bacterium
Source: Mol Syst Biol. 2023 Aug 29;19(10):e11301. doi: 10.15252/msb.202211301 (PMC10568206; doi:10.15252/msb.202211301)
Supplement: Supplementary file 1 — Appendix [file MSB-19-e11301-s002.pdf]

# Appendix

## Table of contents


Table of contents 1

Appendix figures 3

- Appendix Figure S1. Length distribution of RPFs when relaxing mapping quality threshold. 3
- Appendix Figure S2. Reproducibility of ribosome counts and ribosome density and their dependence on mRNA levels. 4
- Appendix Figure S3. Correlation between absolute translation efficiency and mRNA features. 5
- Appendix Figure S4. Correlation between the absolute translation efficiency and other features of the mRNA. 6
- Appendix Figure S5. Correlation between the absolute translation efficiency and the nucleotide identity in the 5'UTR sequence at specific positions upstream of the start codon. 7
- Appendix Figure S6. Correlation between the absolute translation efficiency and the amino acid identity at the N-terminal. 8
- Appendix Figure S7. Correlation between the absolute translation efficiency and the amino acid composition in the first 35 residues at the N-terminal. 9
- Appendix Figure S8. Correlation coefficients between local variations in elongation rate and local mRNA sequence features. 10
- Appendix Figure S9. Coarse-grained metagene profile of ribosome density normalized within each CDS. 11
- Appendix Figure S10. Local impact of the mRNA folding energy on the local variation in elongation rate. 12
- Appendix Figure S11. Relation between the tRNA adaptation index (tAI) derived from the measured tRNA abundances and other observables. 13
- Appendix Figure S12. Correlation between absolute translation efficiency kTEabs and ratio kTEabs/<ρ> with added simulated noise. 13
- Appendix Figure S13. Correlation between translation efficiency to ribosome density ratio and other observables. 14
- Appendix Figure S14. Correlation between the translation efficiency to ribosome density ratio and the GC content of the CDS. 15
- Appendix Figure S15. Correlation between translation efficiency to ribosome density ratio and amino acid composition of protein. 16
- Appendix Figure S16. Absolute translation efficiency for genes classified by leading position in operon, start codon identity and presence of Shine-Dalgarno motif. 17
- Appendix Figure S17. Improving analytical steps in sucrose gradient. 18
- Appendix Figure S18. Percentage of RNA recovered from sucrose cushion pellet in different conditions. 18
- Appendix Figure S19. Coverage of footprints 3' end at the tnaC stalling motif in E. coli. 19

Appendix Figure S20. Sequence composition bias in coding sequences. 20
Appendix Figure S21. Proportion of ribosome occupancy in the three frames. 20
Appendix Figure S22. Calibration curve for absolute quantification of proteins (AQUA). 21
Appendix Figure S23. Deviations between protein abundances estimated from proteome-wide label-free mass spectrometry intensities and absolute protein copy number measured by labeled peptides quantification (AQUA). 22
Appendix Figure S24. Summary of the relative importance of local mRNA sequence features on the local variation in elongation rate. 23
Appendix Figure S25. Comparison of hydro-tRNA-seq relative fragment counts for small fragment sizes v.s. all sizes. 24

## Appendix figures

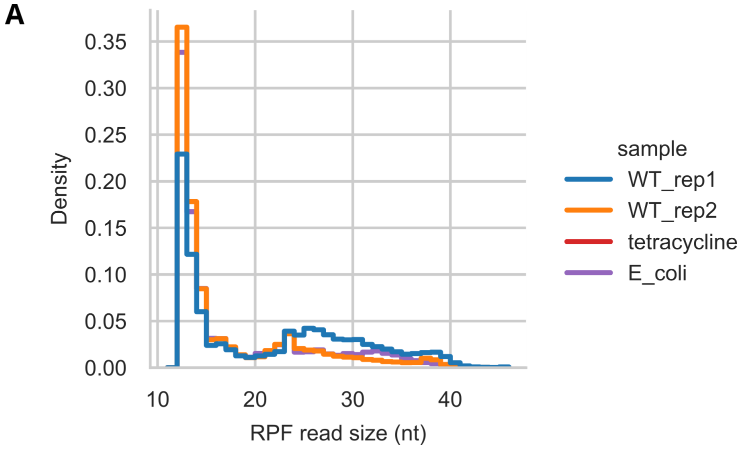


**Appendix Figure S1. Length distribution of RPFs when relaxing mapping quality threshold.**

Ribosome protected footprints length distribution for the main samples when no mapping quality threshold was applied during the alignment of the reads to the genome. In this case, reads mapping to several positions in the genome (multi-mapping reads) were not excluded, resulting in an increase of 12-14 nt long reads.

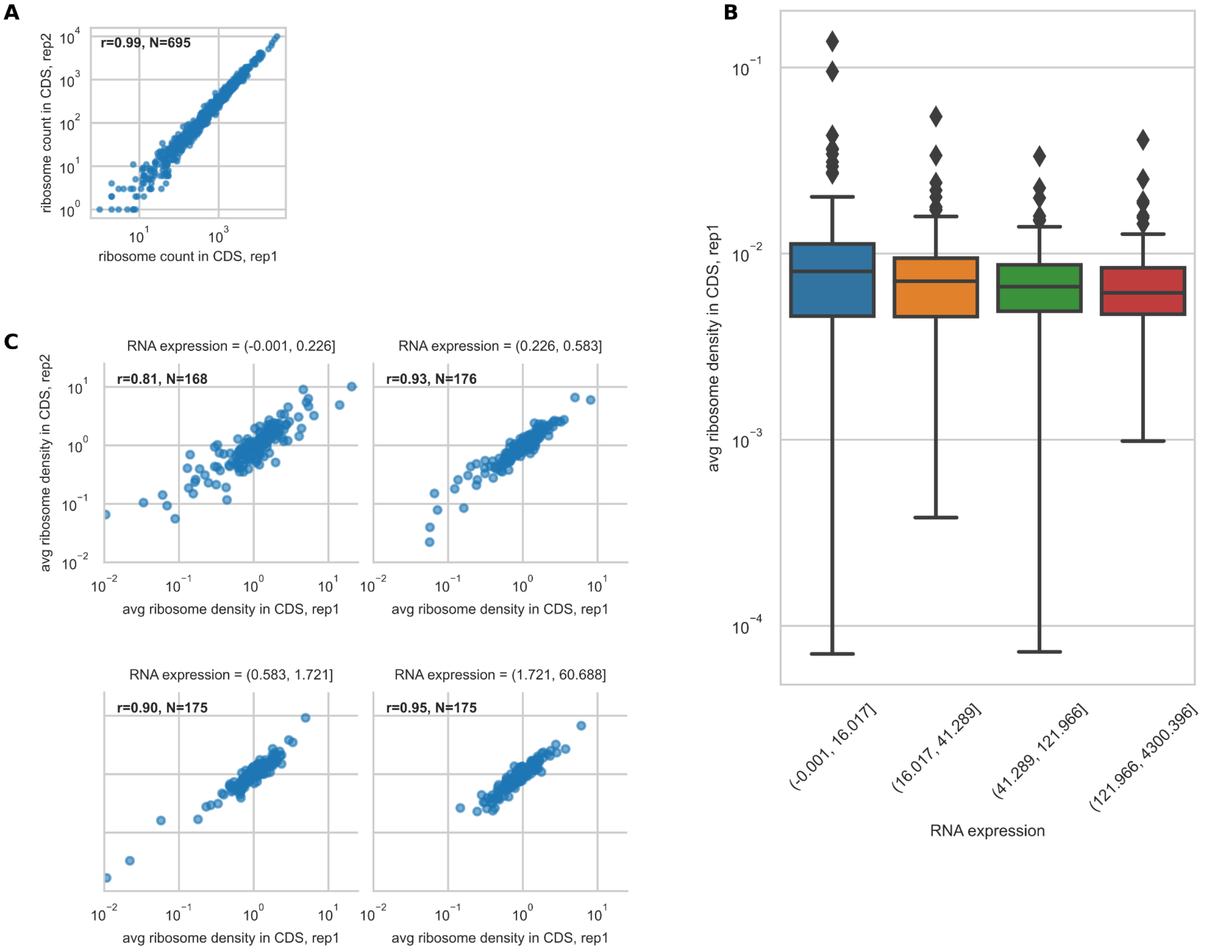


**Appendix Figure S2. Reproducibility of ribosome counts and ribosome density and their dependence on mRNA levels.**

(A) Ribosome counts per gene for the two standard growth replicates rep1 and rep2, and pearson correlation coefficient (inset).

(B) Distribution of average ribosome density in CDSs for the replicate rep1, for CDSs stratified into four groups of normalized RNA expression level, from lowest (left) to highest (right). Ribosome density was overall not related to the level of mRNA expression, as genes with low mRNA levels had a very similar distribution of ribosome density compared to genes with high mRNA levels

(C) The reproducibility of ribosome density at gene level depends on mRNA levels. Correlation of average ribosome density in CDSs for the two replicates rep1 and rep2, for CDSs stratified into four groups of normalized RNA expression level, from lowest (left, top) to highest (right, bottom). Pearson correlation coefficient in linear space and nb of CDSs are displayed for each group. Because mRNA level is a denominator in the expression of the ribosome density, genes with low mRNA levels are particularly sensitive to noise and displayed a lower correlation between replicates (r = 0.81) compared to genes with higher mRNA levels (r = 0.95).

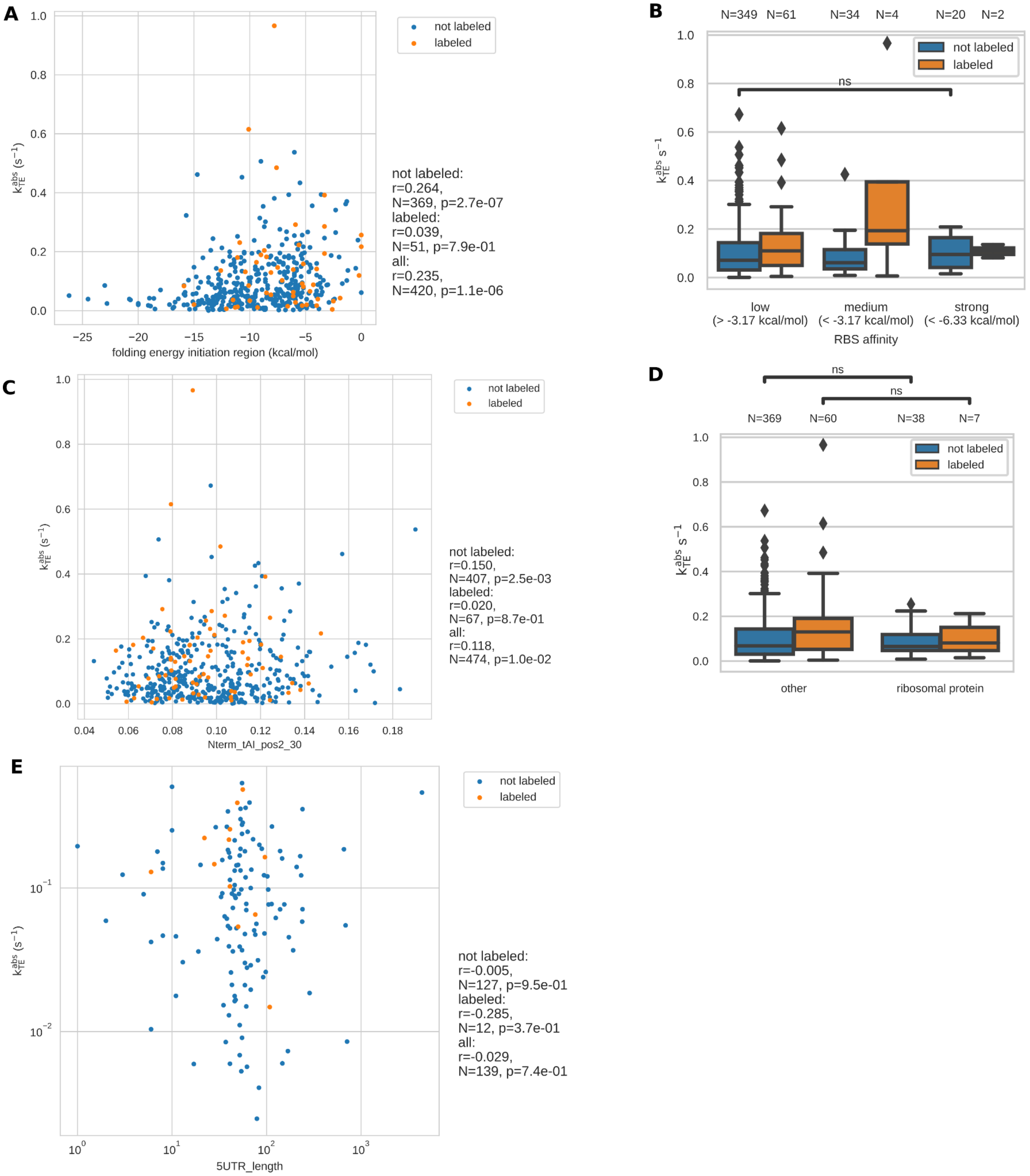


**Appendix Figure S3. Correlation between absolute translation efficiency and mRNA features.**

(A) Correlation between absolute translation efficiency and folding energy in the initiation region, computed in a window of 80 nt centered on the start codon.

(B) Absolute translation efficiency for genes classified by the presence of a ribosome binding site (RBS). The strength of the affinity of the RBS sequence to the anti-Shine-Dalgarno motif CCUCCU was determined by computing the hybridization energy and classified into 3 bins (low, medium and strong affinity). Precision of the translation efficiency was higher for proteins which were quantified by labeled peptides (labeled), compared to proteins quantified by label-free mass spectrometry (not labeled).

(C) Correlation between the absolute translation efficiency and the tRNA adaptation index (tAI) of the first 30 codons at the N-terminal.

(D) Absolute translation efficiency of genes encoding for ribosomal proteins compared to other genes.

(E) Correlation between the absolute translation efficiency and the length of the 5'UTR. Only genes which are at the first position on their operon were selected.

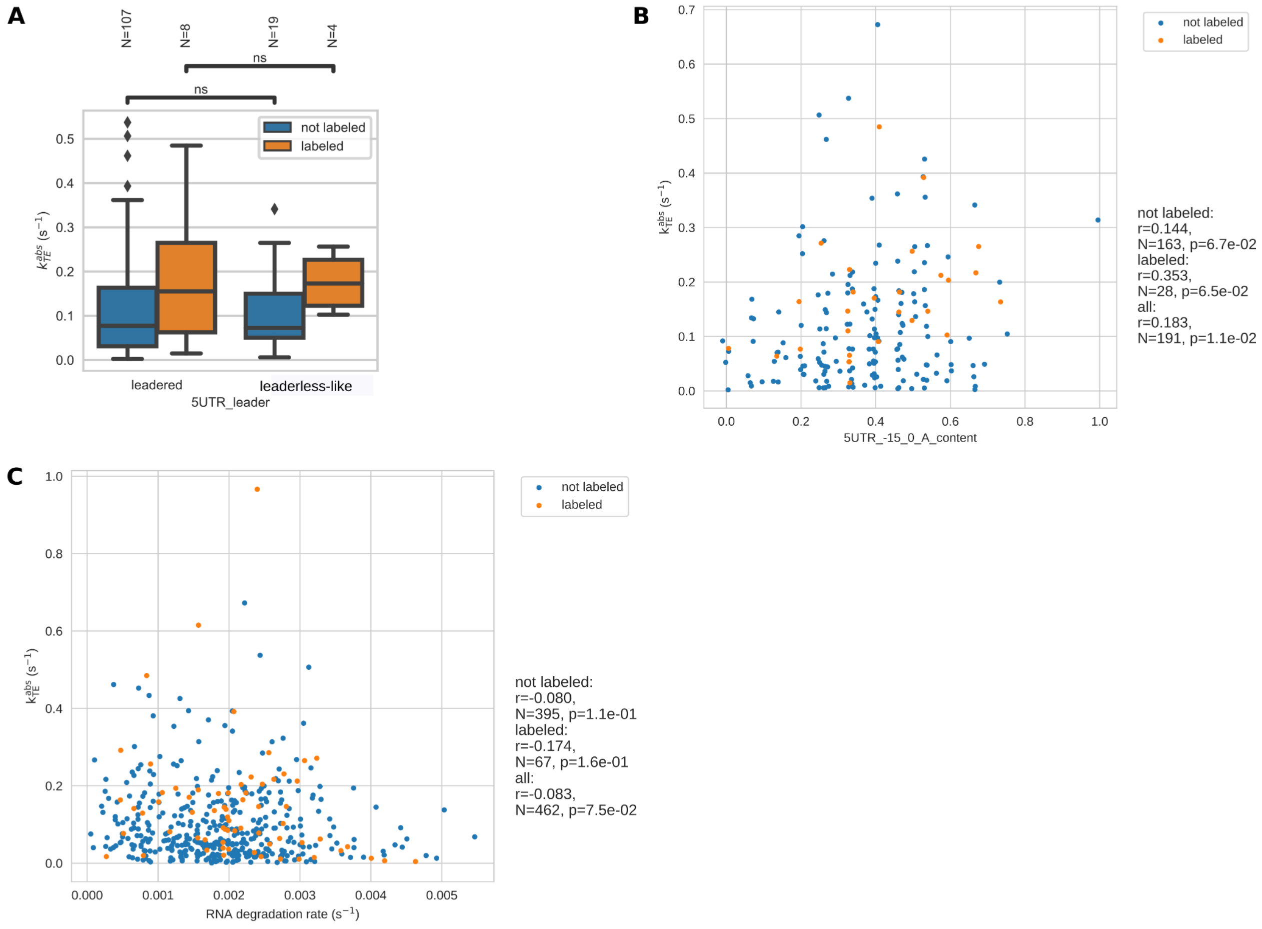


**Appendix Figure S4. Correlation between the absolute translation efficiency and other features of the mRNA.**

(A) Absolute translation efficiency of genes classified by leaderless-like/leadered.

(B) Correlation between the absolute translation efficiency and the nucleotide composition of the 5'UTR close to the start codon. Only genes which are at the first position in their operon were

selected. The A content was computed in a window of 15 nt upstream the start codon. A small jitter was applied to the x position of data points for easier visualization.

(C) Correlation between the absolute translation efficiency and the RNA degradation rate.

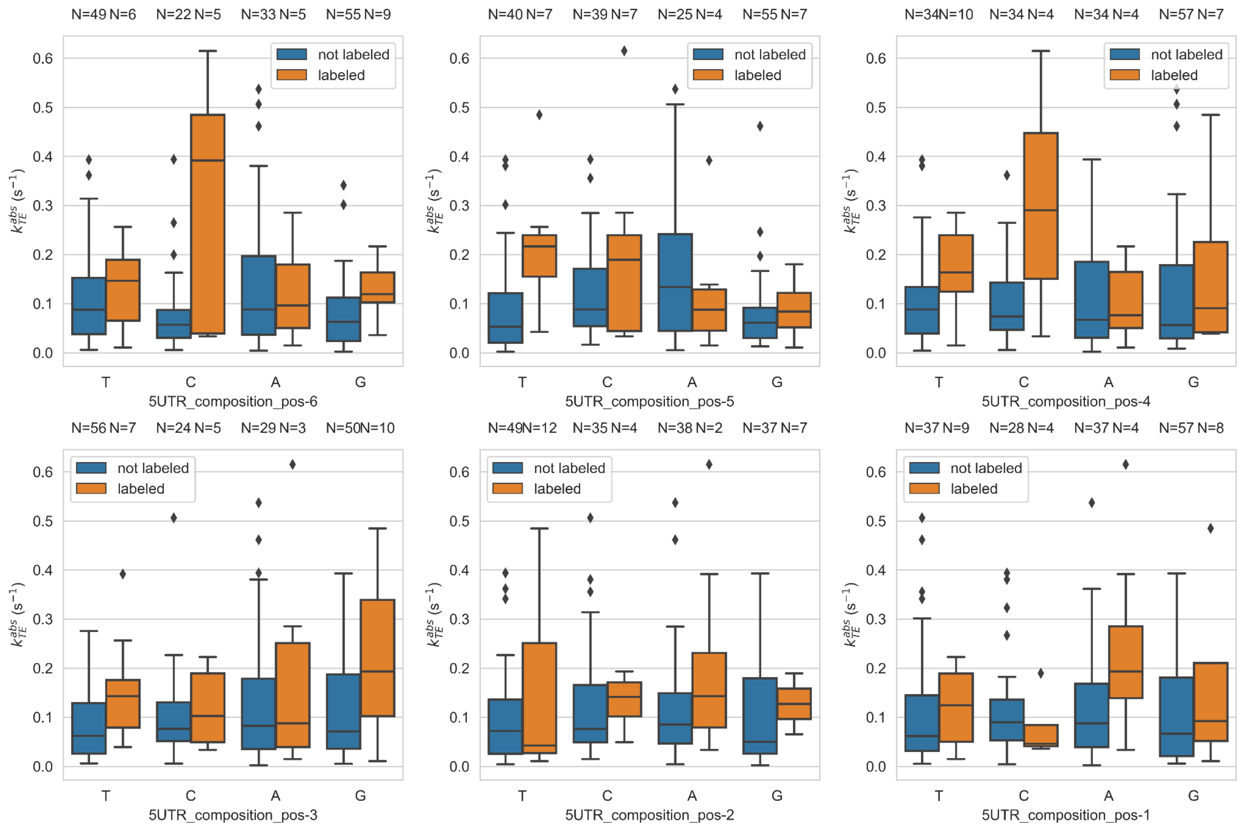


**Appendix Figure S5. Correlation between the absolute translation efficiency and the nucleotide identity in the 5'UTR sequence at specific positions upstream of the start codon.**

Only genes which are at the first position in their operon were selected.

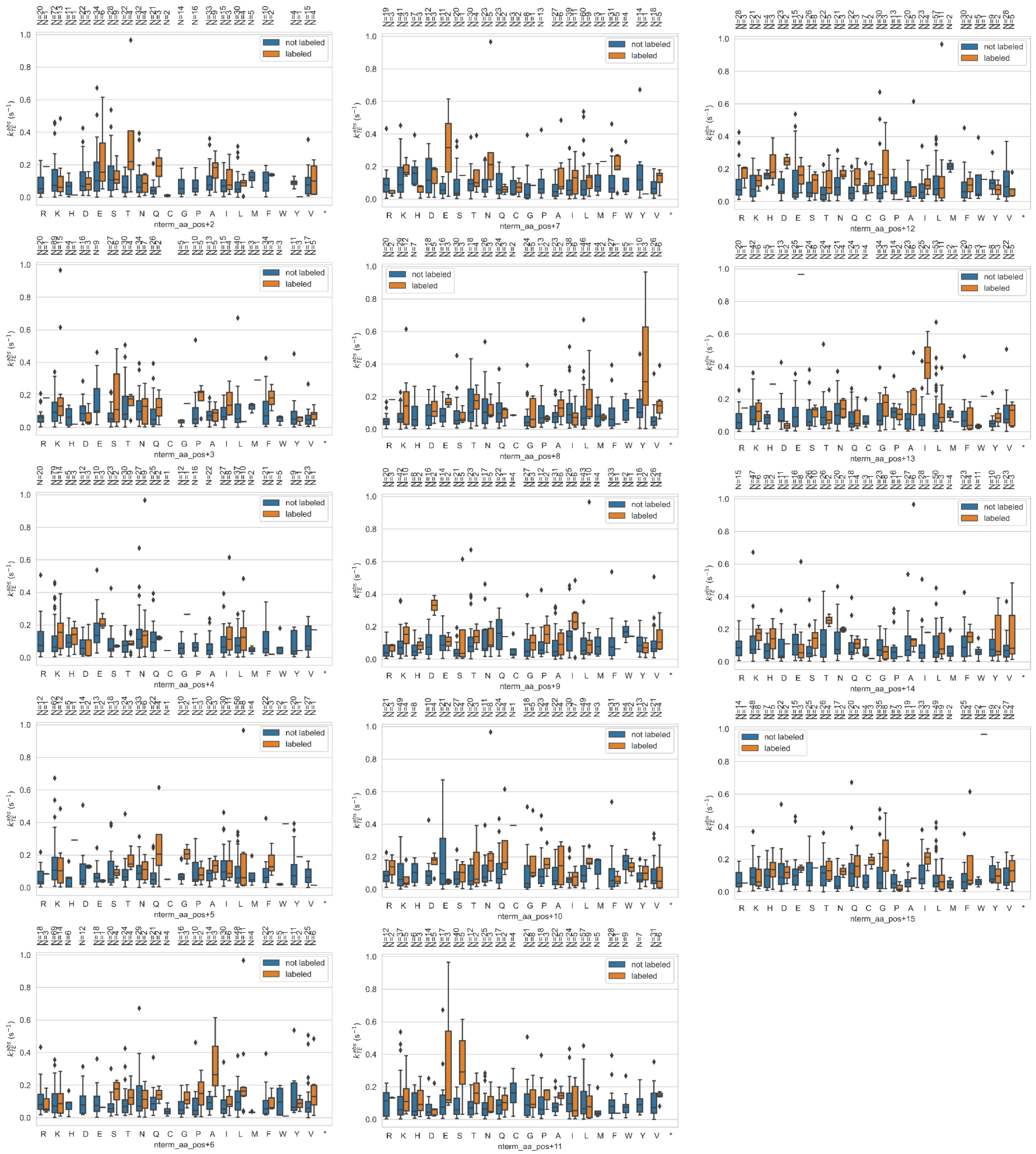


**Appendix Figure S6. Correlation between the absolute translation efficiency and the amino acid identity at the N-terminal.**

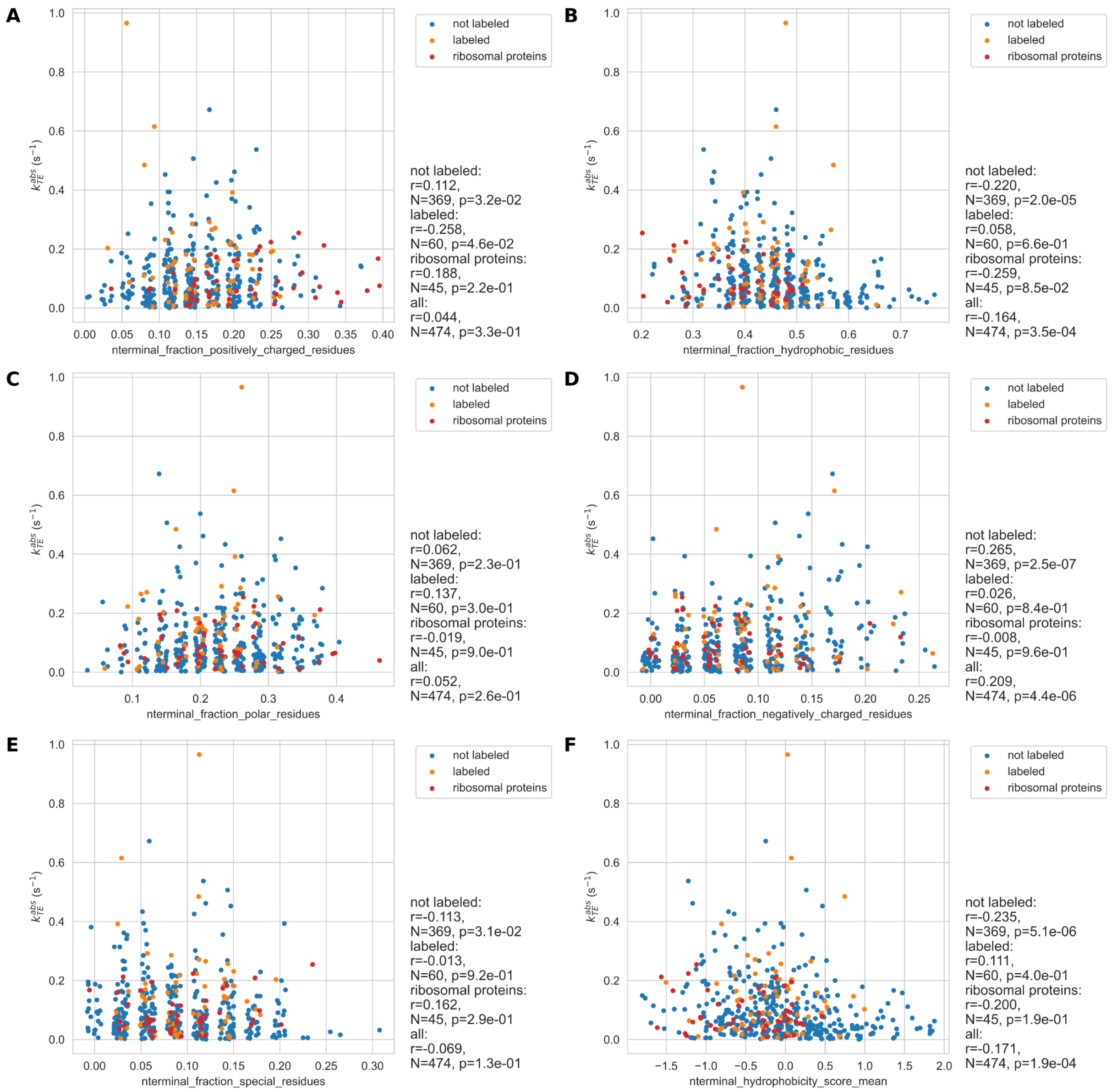


**Appendix Figure S7. Correlation between the absolute translation efficiency and the amino acid composition in the first 35 residues at the N-terminal.**

The amino acid composition at the first 35 residues at the N-terminal of the protein sequences was analyzed based on a broad classification of physico-chemical properties. The translation efficiency $k_{TE}^{abs}$ was compared to the fraction of (A) positively charged residues (Lys, Arg, His), (B) negatively charged residues (Glu, Asp), (C) polar residues (Asn, Gln, Ser, Thr), (D) hydrophobic residues (Ala, Ile, Leu, Met, Val, Phe, Trp, Tyr), (E) other residues (Gly, Pro, Cys), as well as (F) to the average hydrophobic score of amino acids in the CDS computed from the Kyte-Doolittle hydrophobicity scores. In the case of plots of the fraction of residues, a small jitter was applied to the horizontal axis in order to improve visualization.

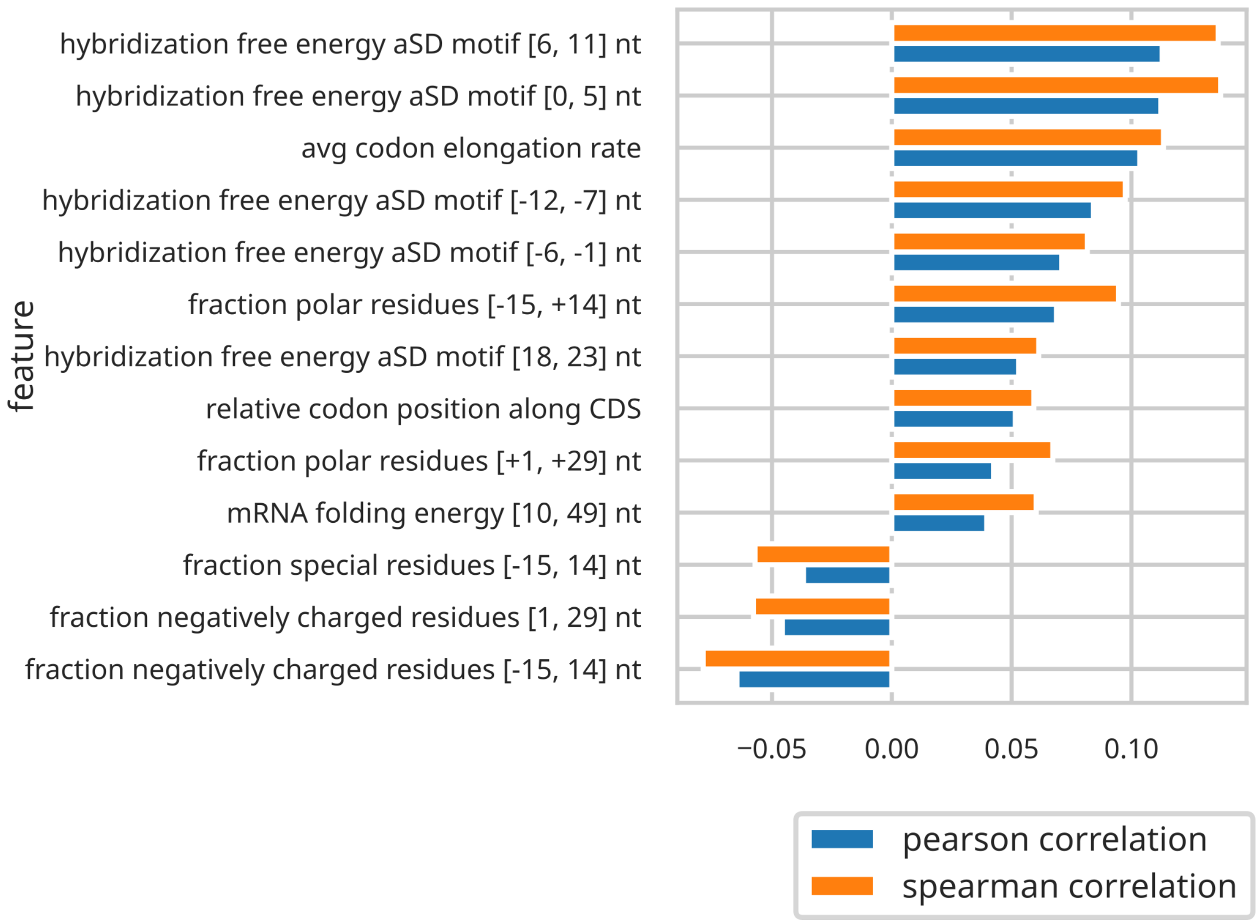


**Appendix Figure S8. Correlation coefficients between local variations in elongation rate and local mRNA sequence features.**

Pearson and Spearman correlation coefficients between local features of the mRNA sequence and the local variation of elongation rate $k_{elong,i}^{rel}$ determined from the ribosome density and normalized within each gene.

A B

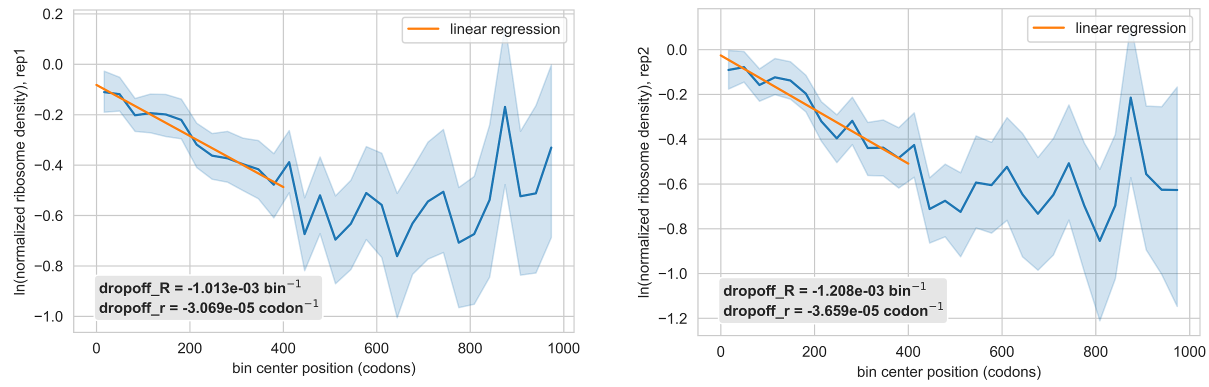


**Appendix Figure S9. Coarse-grained metagene profile of ribosome density normalized within each CDS.**

Metagene profile of ribosome density normalized within each CDS, aligned to the first nucleotide of the start codon, and averaged in bins of 33 codons (99 nt). (A) Replicate 1, (B) Replicate 2. Mean across all CDSs (line) and 95% confidence interval (shaded area).

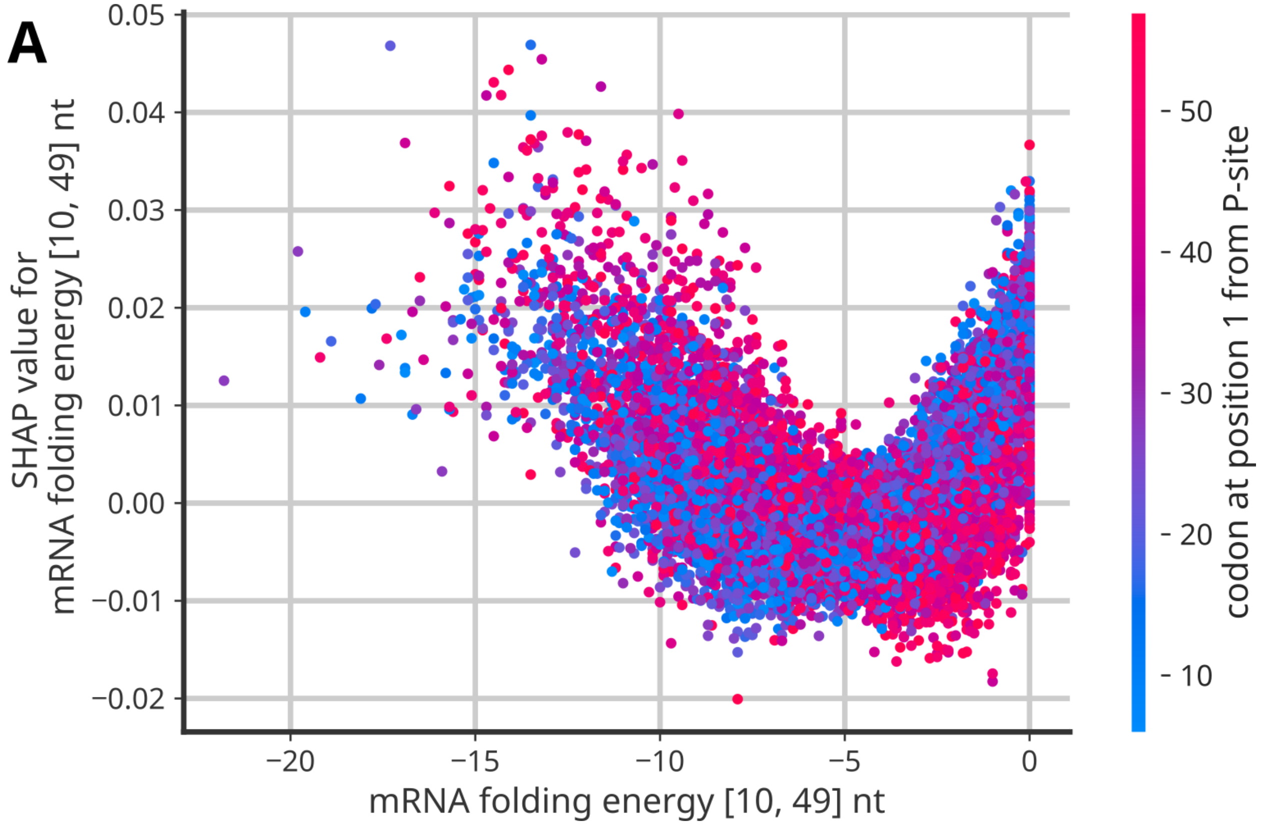


**Appendix Figure S10. Local impact of the mRNA folding energy on the local variation in elongation rate.**

The local impact SHAP values (vertical axis) were plotted against the mRNA folding energy in the window [10, 49] nt, for all the codon positions in the test set (dots). Data points were color coded depending on the encoded numerical value of the codon identity at the A-site (position +1 from the P-site), from 0 to 62 (color code), ordered by their average codon elongation rate.

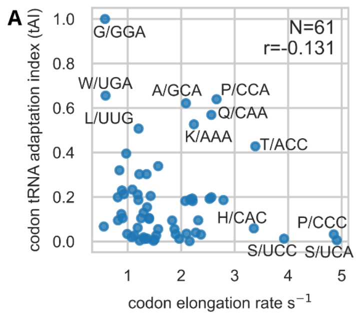

**Appendix Figure S11. Relation between the tRNA adaptation index (tAI) derived from the measured tRNA abundances and other observables.**

(A) Comparison between tAI of codons determined by measured tRNA abundances with the codon elongation rates determined by ribosome profiling. Pearson correlation coefficient (r).

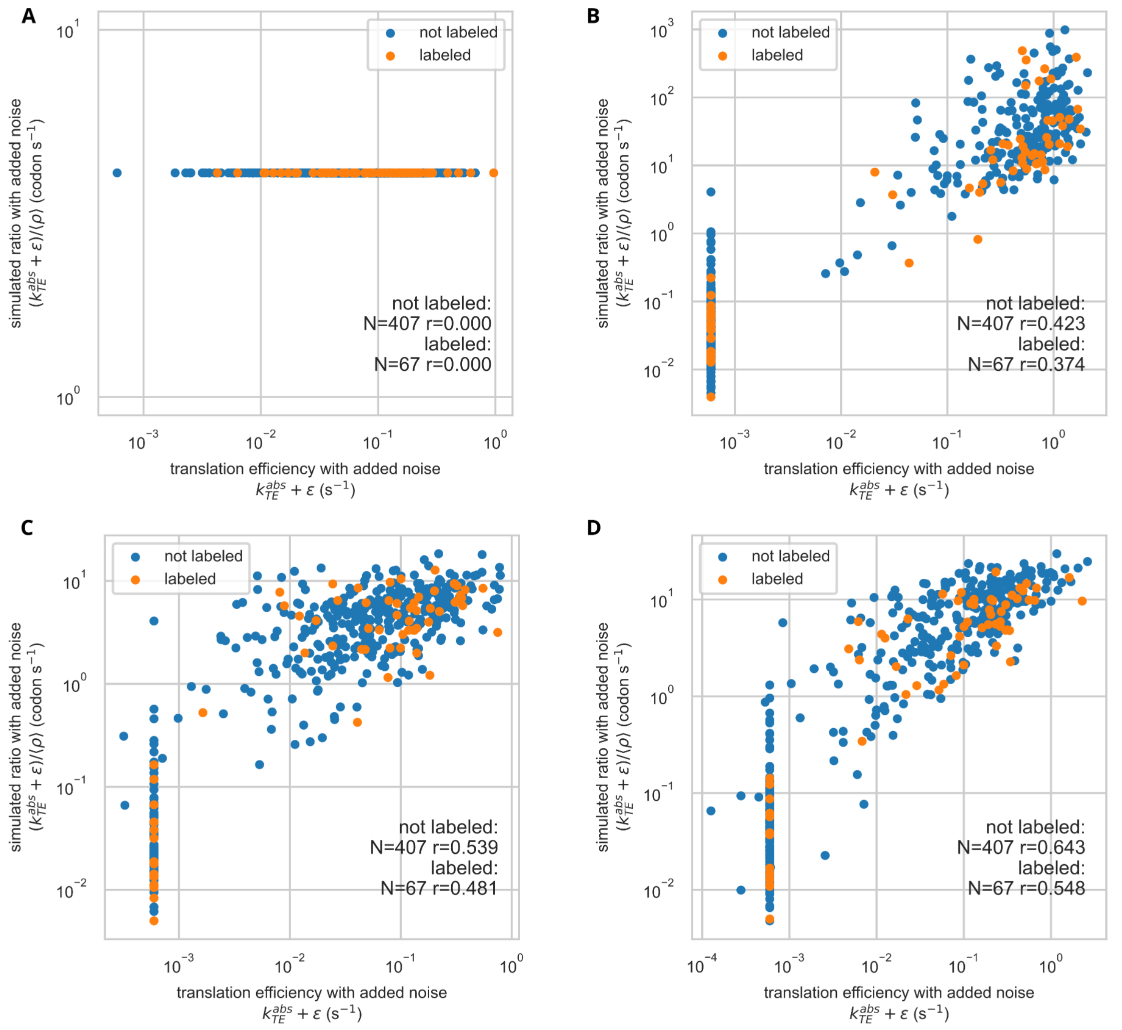


**Appendix Figure S12. Correlation between absolute translation efficiency $k_{TE}^{abs}$ and ratio $k_{TE}^{abs}/\langle\rho\rangle$ with added simulated noise.**

We assumed that the ratio $k_{TE}^{abs}/\langle\rho\rangle$, a proxy for the average elongation rate, is constant and identical for all genes, and derived the simulated ribosome densities $\langle\rho\rangle$. Simulated negative values were clipped to the minimum experimental value of $k_{TE}^{abs}$ (6e-4 s$^{-1}$). (A) Without noise, the values of the ratio lie on a horizontal line when plotted against $k_{TE}^{abs}$. (B) With a gaussian noise added to the value of $k_{TE}^{abs}$, with fixed standard deviation equal to 10 times the median value of $k_{TE}^{abs}$. (C) Gaussian noise with coefficient of variation CV=1, i.e. a variable standard deviation equal to the value of $k_{TE}^{abs}$. (D) Gaussian noise with coefficient of variation CV=2, i.e. a variable standard deviation equal to 2 times the value of $k_{TE}^{abs}$.

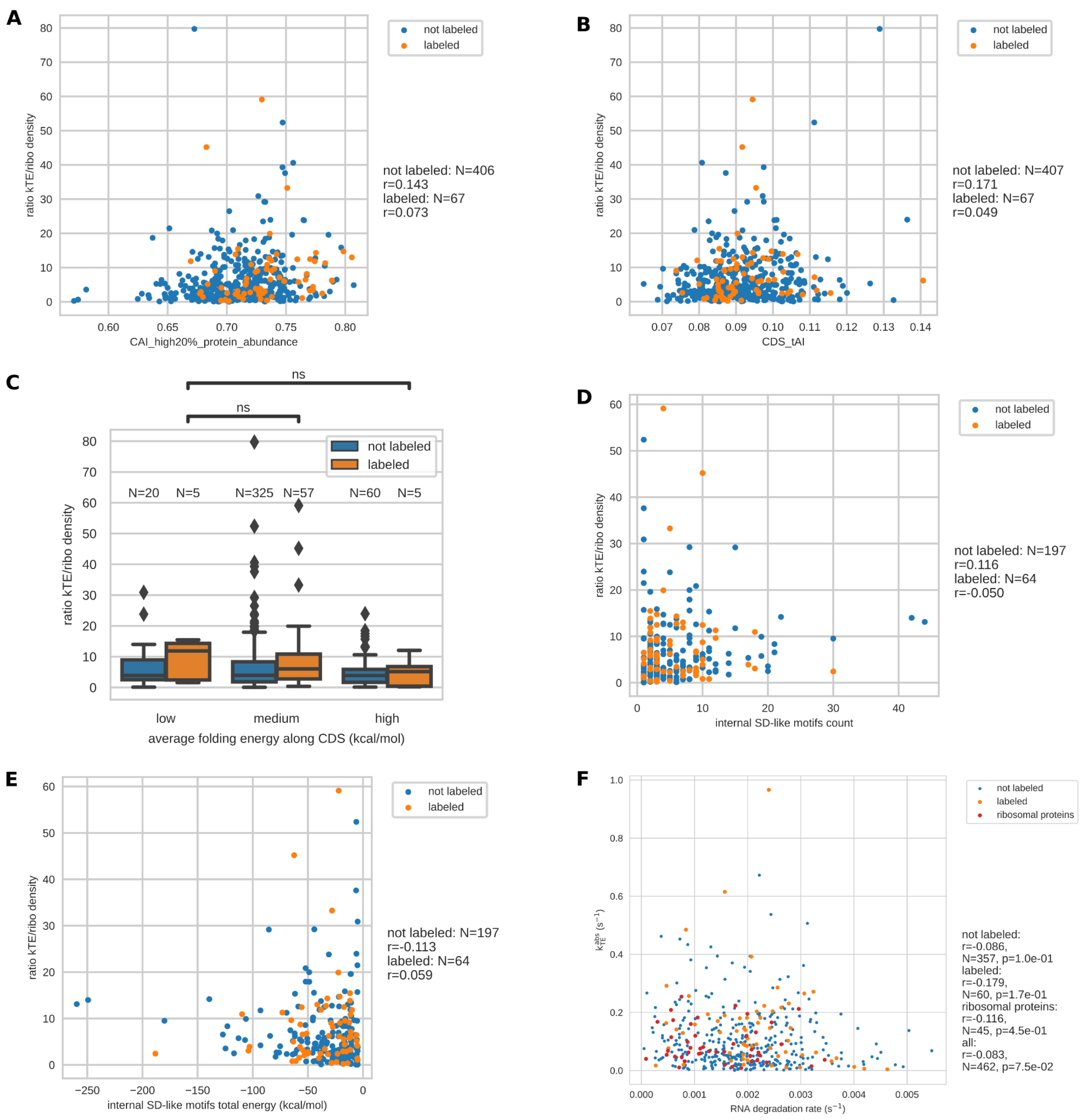


**Appendix Figure S13. Correlation between translation efficiency to ribosome density ratio and other observables.**

(A) Correlation between translation efficiency to ribosome density ratio and codon adaptation index (CAI). The CAI was computed based on the codon usage of the proteins with top 20% protein abundance.

(B) Correlation between translation efficiency to ribosome density ratio and tRNA adaptation index (tAI).

(C) Translation efficiency to ribosome density ratio and folding energy along the CDS. The folding energy was computed along the CDS in a rolling window of size 60 nt and averaged over the CDS, and then categorized into 3 bins: low (< -11.6 kcal/mol), medium (< -7.82 kcal/mol) and high (> -7.82 kcal/mol).

(D-E) Correlation between translation efficiency to ribosome density ratio and internal SD-like motifs. The possible influence of internal SD-like motifs was tested in two ways. (E) The count of internal SD-like motifs within the CDS. (E) The sum of the free energies of each SD-like motif within the CDS.

(F) Correlation between the translation efficiency to ribosome density ratio and the RNA degradation rate.

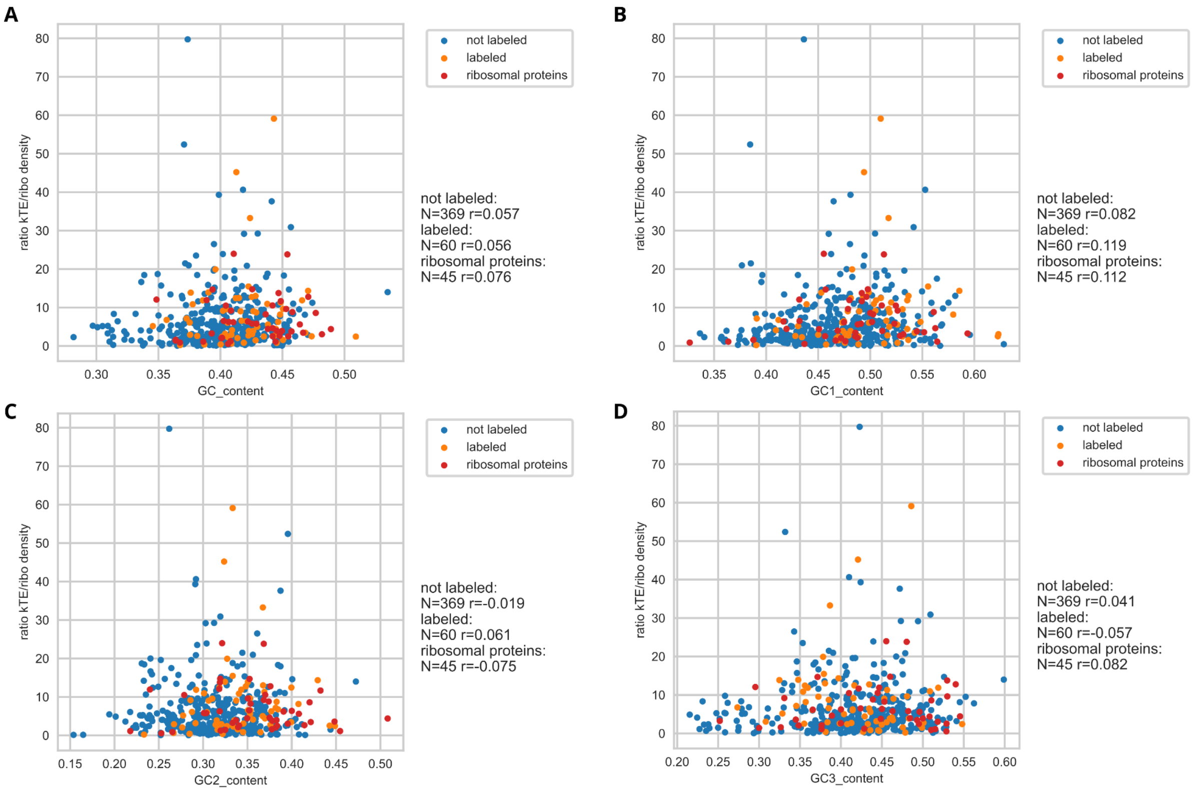


**Appendix Figure S14. Correlation between the translation efficiency to ribosome density ratio and the GC content of the CDS.**

(A) GC content of the CDS

(B) GC content at the first nucleotide position of codons (GC1)

(C) GC content at the second nucleotide position of codons (GC2)

(D) GC content at the third nucleotide position of codons (GC3).

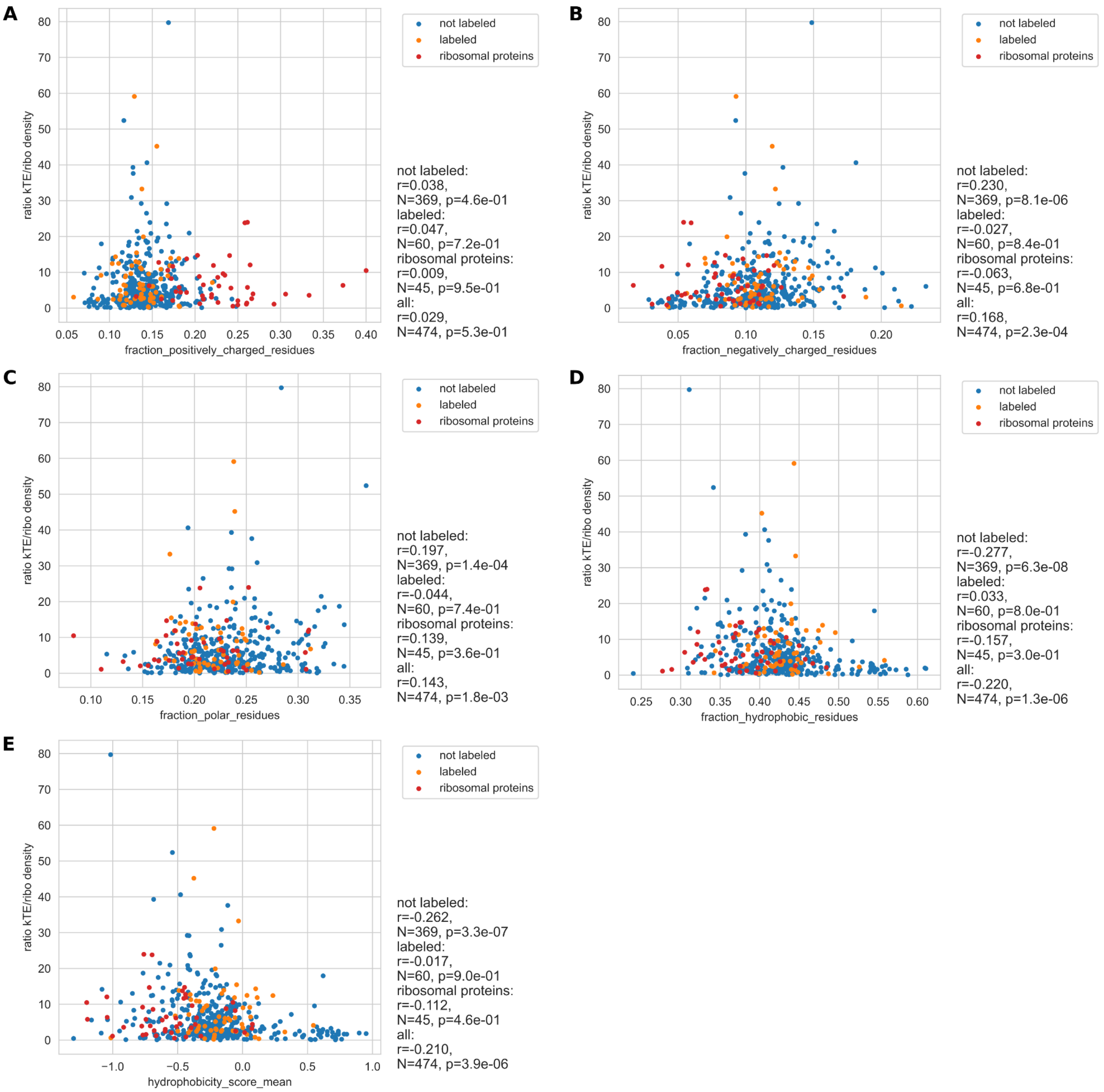


**Appendix Figure S15. Correlation between translation efficiency to ribosome density ratio and amino acid composition of protein.**

The amino acid composition of the protein sequences was analyzed based on a broad classification of physico-chemical properties. The ratio $k_{TE}^{abs}/\langle\rho\rangle$ was compared to the fraction of (A) positively charged residues (Lys, Arg, His), (B) negatively charged residues (Glu, Asp), (C) polar residues (Asn, Gln, Ser, Thr), (D) hydrophobic residues (Ala, Ile, Leu, Met, Val, Phe, Trp, Tyr), as well as (E) to the average hydrophobic score of amino acids in the CDS computed from the Kyte-Doolittle hydrophobicity scores.

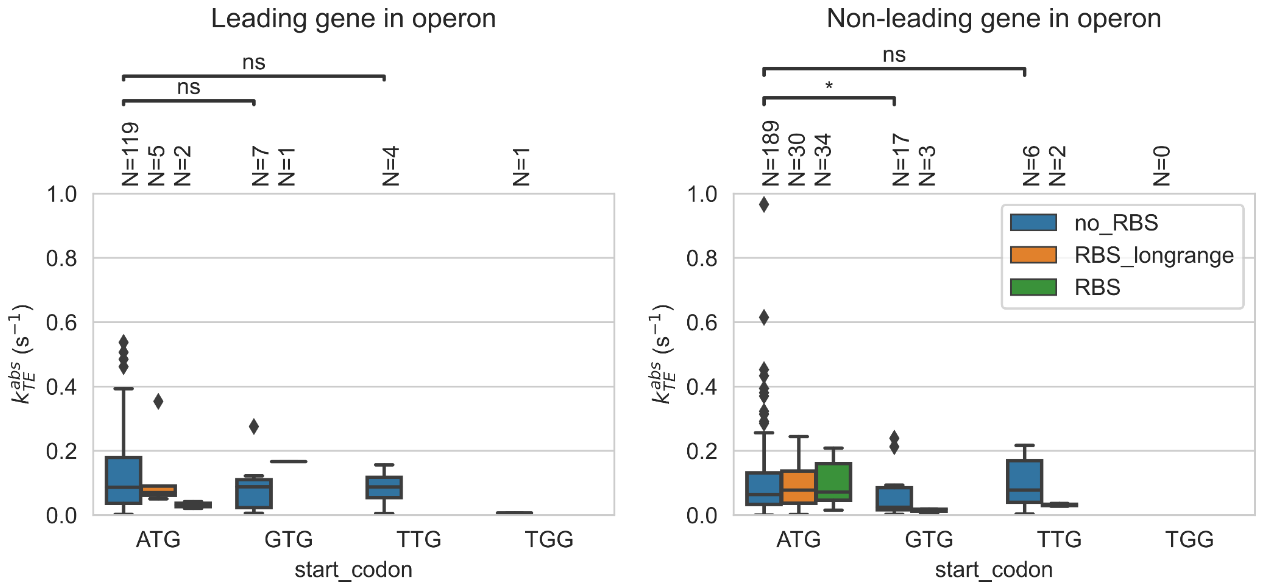


**Appendix Figure S16. Absolute translation efficiency for genes classified by leading position in operon, start codon identity and presence of Shine-Dalgarno motif.**

Non-leading genes in operon with the ATG start codon and no RBS exhibited a higher translation efficiency compared to non-leading genes with the GTG start codon and no RBS (M.W.W. two-sided p=1.3e-02). P-value legend: ns $p > 0.05$; * $p < 0.05$.

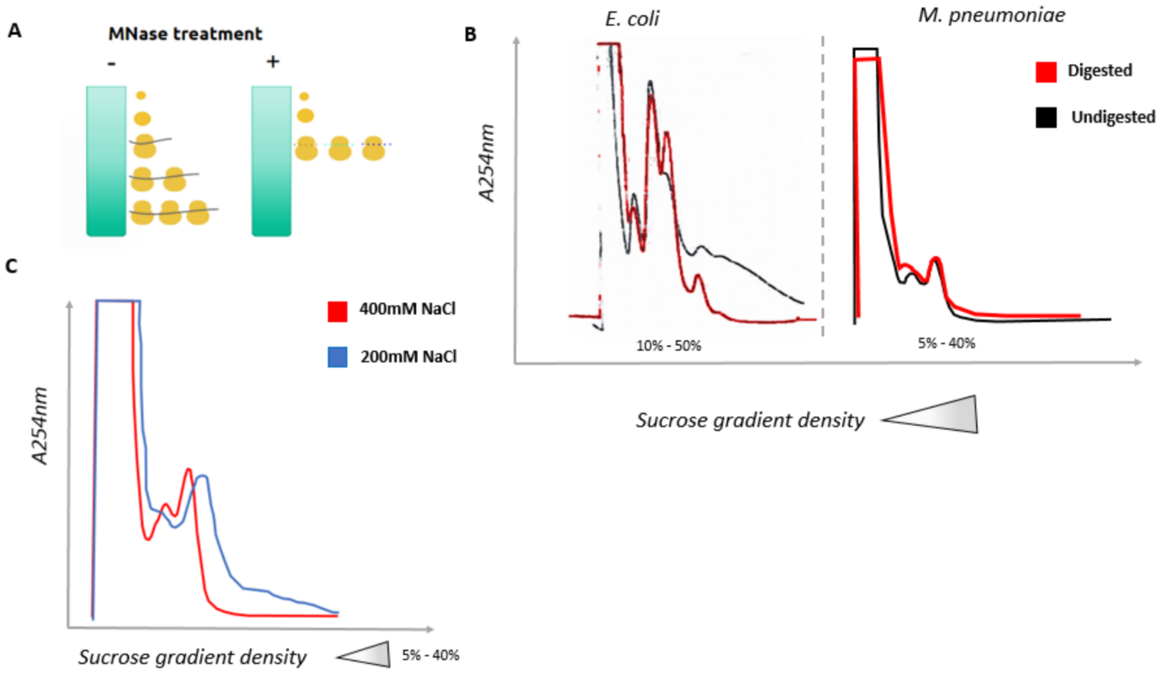


**Appendix Figure S17. Improving analytical steps in sucrose gradient.**

(A) Monosomes and polysomes travel through the sucrose gradient until they reach the point at which their density matches that of the surrounding sucrose. In the untreated lysate, polysomes with two, three or more ribosomes are kept intact. Upon MNase treatment, all polysomes are converted to monosomes. (B) Polysome profile in *E. coli* (left) and *M. pneumoniae* (right), for digested (red line) and undigested (black line) samples. (C) Polysome profile in *M. pneumoniae* using different NaCl concentrations: 400 mM NaCl (red line), 200 mM NaCl (blue line).

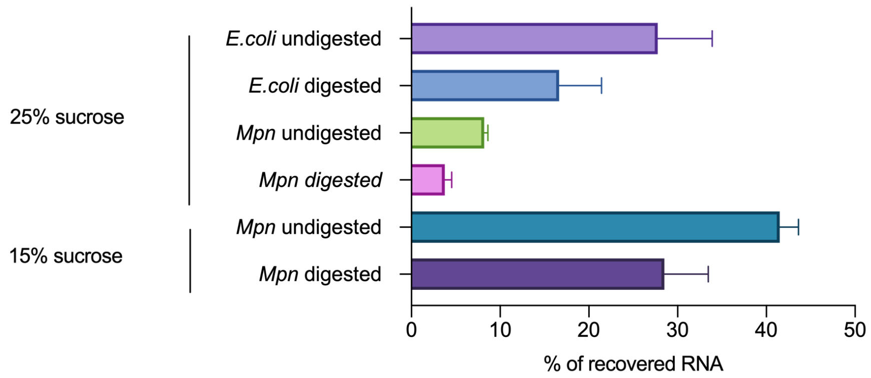


**Appendix Figure S18. Percentage of RNA recovered from sucrose cushion pellet in different conditions.**

Two concentrations of sucrose were tested (left labels) for the sucrose cushions, as well as two different conditions: digestion / no digestion, for *E. coli* and *Mpn*. Bars represent the % of recovered RNA after sucrose cushion pellet extraction.

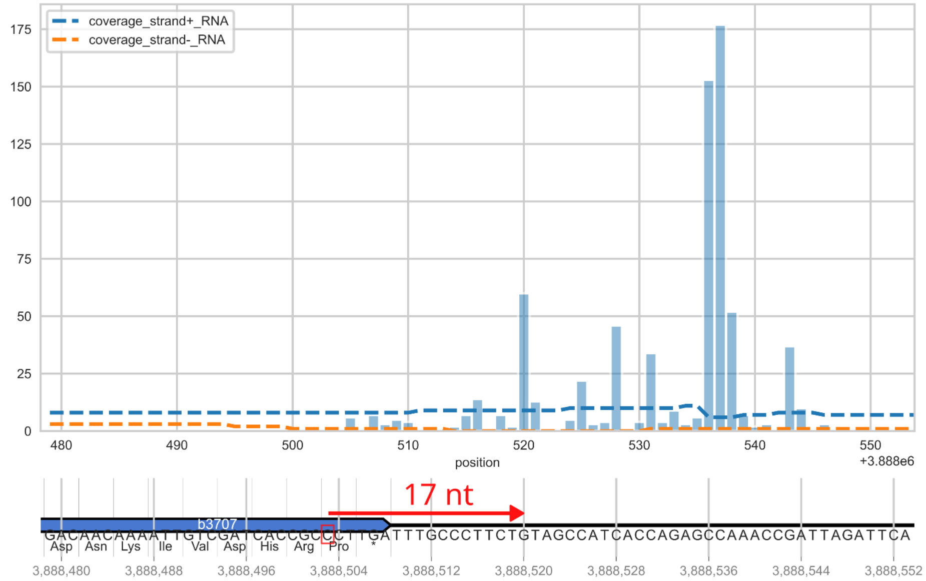


**Appendix Figure S19. Coverage of footprints 3' end at the tnaC stalling motif in *E. coli*.**

Coverage of the 3' end of ribosome footprints in the *E. coli* sample, in the region of the C-terminal IVDHRP* translation stalling motif in *tnaC*. The stalling motif is expected to produce a peak of stalled ribosomes with their P-site positioned at the last proline codon. A peak in the coverage of footprints 3' ends was observed at a distance of 17 nt from the first nucleotide of the proline codon (red arrow).

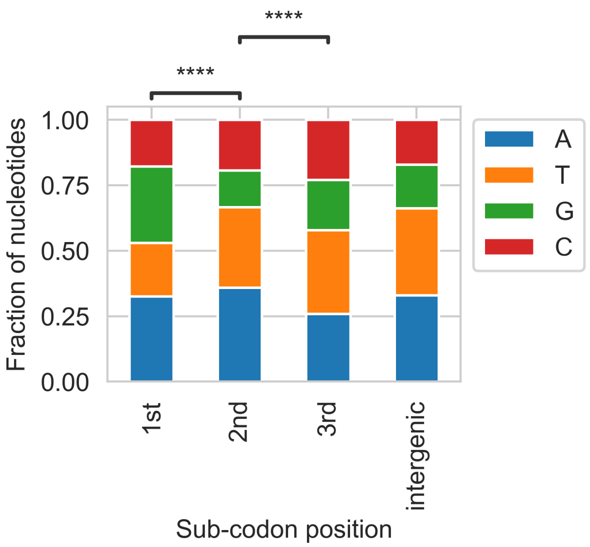


**Appendix Figure S20. Sequence composition bias in coding sequences.**

Nucleotide composition at the first, second and third nucleotide position of all codons in *Mpn*, as well as in intergenic regions. The second sub-codon position shows a higher fraction of A and T compared to the first (Fisher exact test two-sided, P≅0) and third (Fisher exact test two-sided, p≅0) positions. P-value legend: ****P < $10^{-4}$.

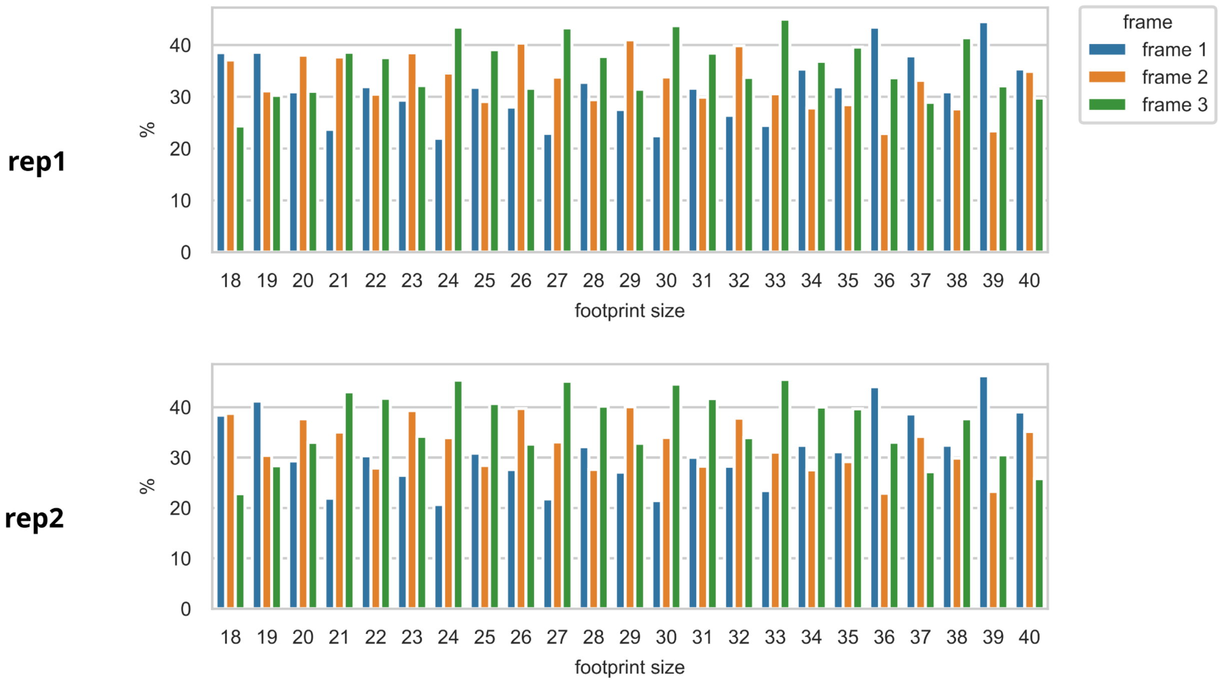


**Appendix Figure S21. Proportion of ribosome occupancy in the three frames.**

Average proportion of ribosome occupancy in the three frames for all CDSs, for each footprint size, for the standard growth exponential phase samples (rep1, rep2).

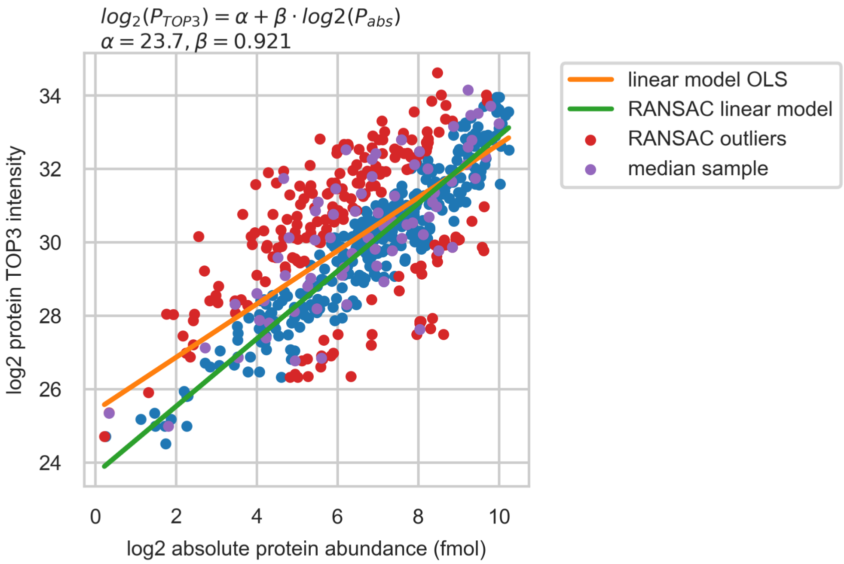


**Appendix Figure S22. Calibration curve for absolute quantification of proteins (AQUA).**

A robust linear regression using the RANSAC method was performed on the absolute amount of each peptide of the SRM assay ($P_{abs}$) and the protein top 3 intensity of the Orbitrap ($P_{TOP3}$), using all peptide values for all replicates and the three growth time points. The median sample used as a reference for normalization of external samples is also shown.

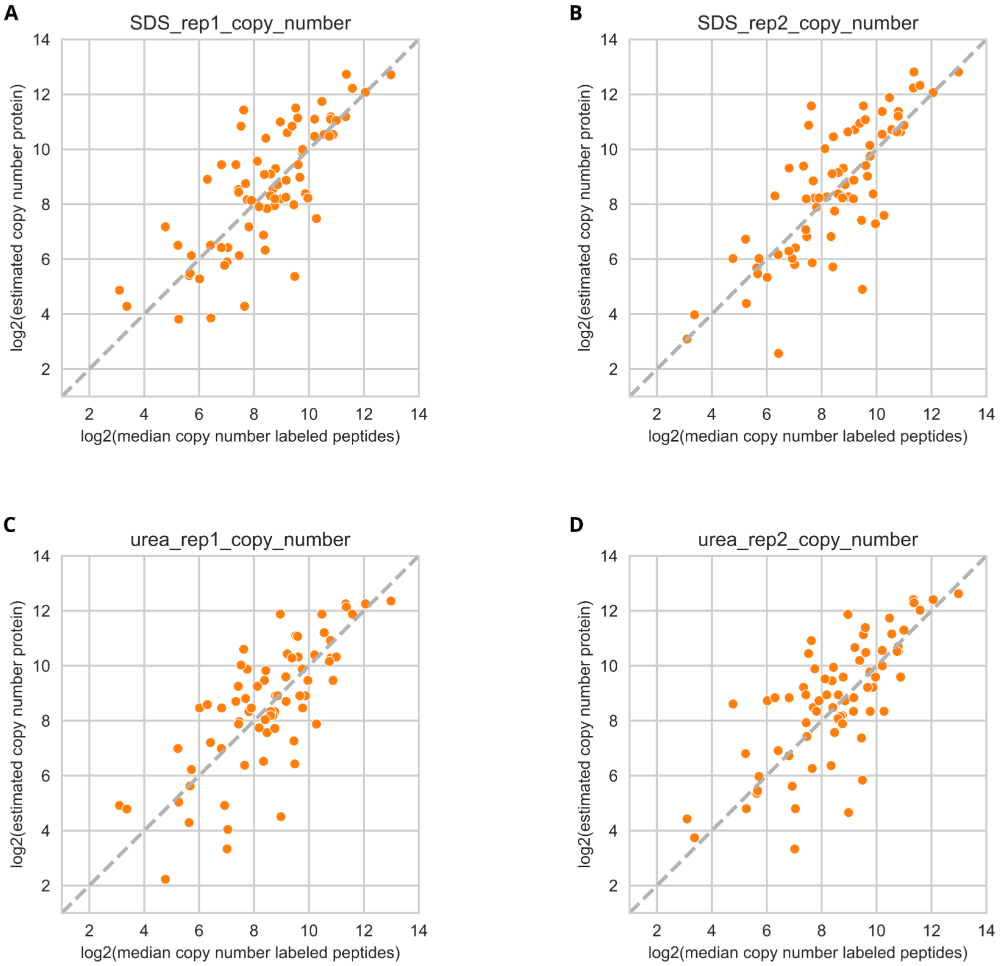


**Appendix Figure S23. Deviations between protein abundances estimated from proteome-wide label-free mass spectrometry intensities and absolute protein copy number measured by labeled peptides quantification (AQUA).**

Protein abundances in copy per cell were estimated from the proteome-wide label-free tandem mass spectrometry protein intensities, for two samples using gel-based protein extraction method (panel A, SDS_rep1, and panel B, SDS_rep2), and two samples using gel-free urea protein extraction method (panel C, urea_rep1, panel D, urea_rep2). The estimated protein abundances were plotted against the median protein copy numbers derived from the measured absolute abundances of labeled peptides in the AQUA experiment (horizontal axis, same data in all plots).

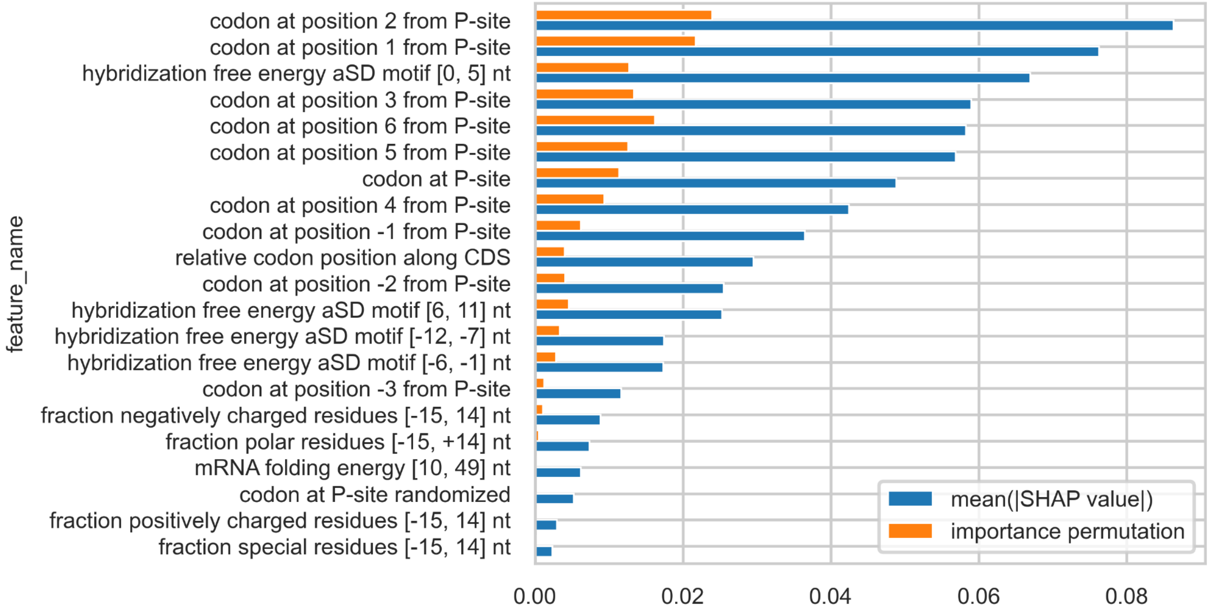


**Appendix Figure S24. Summary of the relative importance of local mRNA sequence features on the local variation in elongation rate.**

Relative importance of features in the random forest regressor were estimated by two means: i) by permuting the values of each feature independently and reporting the decrease in the regressor prediction score (importance permutation), ii) reporting the mean impact of each individual feature on the model output, as computed by the SHAP values of the TreeExplainer method (mean |SHAP| values). In both methods, only the test set was used to estimate relative feature importances.

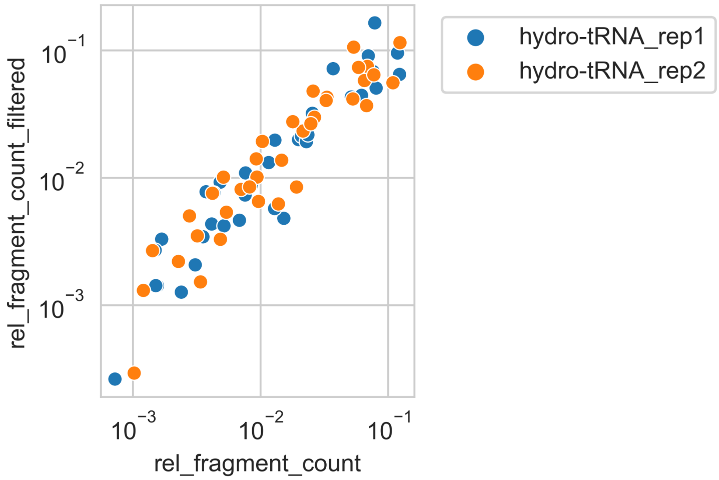


**Appendix Figure S25. Comparison of hydro-tRNA-seq relative fragment counts for small fragment sizes v.s. all sizes.**

Relative fragment counts (normalized by the total counts) for the two replicates of the hydro-tRNA-seq experiment for each tRNA gene, when considering all fragment sizes (horizontal axis) or only fragments of size <= 40 nt (vertical axis). The range of variation of fragment counts for all tRNA genes (~two orders of magnitude) is very similar between the two datasets.
