# Supplementary material for: Comprehensive quantitative modeling of translation efficiency in a genome‐reduced bacterium
Source: Mol Syst Biol. 2023 Aug 29;19(10):e11301. doi: 10.15252/msb.202211301 (PMC10568206; doi:10.15252/msb.202211301)
Supplement: Supplementary file 11 [file MSB-19-e11301-s010.pdf]

molecular systems biology

Comprehensive quantitative modeling of translation efficiency in a genome-reduced bacterium

Marc Weber, Adria Sogues, Eva Yus, Raul Burgos, Carolina Gallo, Sira Martinez, Maria Lluch-Senar, and Luis Serrano
DOI: 10.15252/msb.202211301

Corresponding author(s): Luis Serrano (luis.serrano@crg.eu)

Review Timeline:

Submission Date:	16th Aug 22
Editorial Decision:	16th Sep 22
Revision Received:	29th May 23
Editorial Decision:	4th Jul 23
Revision Received:	17th Jul 23
Accepted:	24th Jul 23

Editor: Maria Polychronidou

Transaction Report:

(Note: With the exception of the correction of typographical or spelling errors that could be a source of ambiguity, letters and reports are not edited. Depending on transfer agreements, referee reports obtained elsewhere may or may not be included in this compilation. Referee reports are anonymous unless the Referee chooses to sign their reports.)

16th Sep 2022

RE: MSB-2022-11301, Comprehensive quantitative modeling of translation efficiency in a genome-reduced bacterium

Dear Luis,

Thank you again for submitting your work to Molecular Systems Biology. We have now heard back from the three reviewers who agreed to evaluate your study. As you will see below, the reviewers appreciate that the study represents a relevant contribution to the field. However, they raise a series of concerns, which we would ask you to address in a revision.

I think that the reviewers' recommendations are rather clear and I therefore see no need to repeat any of the comments listed below. All issues raised by the referees would need to be satisfactorily addressed. Please let me know in case you would like to discuss in further detail any of the issues raised, I would be happy to schedule a call.

On a more editorial level, we would ask you to address the following points:

- Please provide a .doc version of the manuscript text (including legends for the main figures) and individual production quality figure files for the main Figures (one file per figure).

- Please provide 5 keywords.

- We have replaced Supplementary Information by the Expanded View (EV format). In this case, all additional figures can be included in a PDF called Appendix. Appendix figures should be labeled and called out as: "Appendix Figure S1, Appendix Figure S2... Appendix Table S1..." etc. Each legend should be below the corresponding Figure/Table in the Appendix. Please include a Table of Contents in the beginning of the Appendix. For detailed instructions regarding expanded view please refer to our Author Guidelines: .

- Tables S1-S9 should be provided as Datasets EV1-EV9 (one file per EV Dataset). Please include in each .xls file a description of the table in a separate tab.

- Please provide a "standfirst text" summarizing the study in one or two sentences (approximately 250 characters), three to four "bullet points" highlighting the main findings and a "synopsis image" (550px width and max 400px height, jpeg format) to highlight the paper on our homepage.

- All Materials and Methods need to be described in the main text (and NOT in the Appendix). We would encourage you to use 'Structured Methods', our new Materials and Methods format. According to this format, the Material and Methods section should include a Reagents and Tools Table (listing key reagents, experimental models, software and relevant equipment and including their sources and relevant identifiers) followed by a Methods and Protocols section in which we encourage the authors to describe their methods using a step-by-step protocol format with bullet points, to facilitate the adoption of the methodologies across labs. More information on how to adhere to this format as well as downloadable templates (.doc or .xls) for the Reagents and Tools Table can be found in our author guidelines: . An example of a Method paper with Structured Methods can be found here: .

- Please include a "Disclosure Statement & Competing Interests" in the main text.

- Please include a Data availability section describing how the data, code etc. have been made available. This section needs to be formatted according to the example below:
The datasets and computer code produced in this study are available in the following databases:
- Chip-Seq data: Gene Expression Omnibus GSE46748 (https://www.ncbi.nlm.nih.gov/geo/query/acc.cgi?acc=GSE46748)
- Modeling computer scripts: GitHub (https://github.com/SysBioChalmers/GECKO/releases/tag/v1.0)
- [data type]: [full name of the resource] [accession number/identifier] ([doi or URL or identifiers.org/DATABASE:ACCESSION])

- The References should be formatted according to the Molecular Systems Biology reference style.

- For data quantification: please specify the name of the statistical test used to generate error bars and P values, the number (n) of independent experiments (specify technical or biological replicates) underlying each data point and the test used to calculate p-values in each figure legend. The figure legends should contain a basic description of n, P and the test applied. Graphs must include a description of the bars and the error bars (s.d., s.e.m.).

- When you resubmit your manuscript, please download our CHECKLIST (https://bit.ly/EMBOPressAuthorChecklist) and include the completed form in your submission.
Please note that the Author Checklist will be published alongside the paper as part of the transparent process

(https://www.embopress.org/page/journal/17444292/authorguide#transparentprocess).

If you feel you can satisfactorily deal with these points and those listed by the referees, you may wish to submit a revised version of your manuscript. Please attach a covering letter giving details of the way in which you have handled each of the points raised by the referees. A revised manuscript will be once again subject to review and you probably understand that we can give you no guarantee at this stage that the eventual outcome will be favorable.

Kind regards,

Maria

Maria Polychronidou, PhD
Senior Editor
Molecular Systems Biology

We realize that it is difficult to revise to a specific deadline. In the interest of protecting the conceptual advance provided by the work, we recommend a revision within 3 months (15th Dec 2022). Please discuss the revision progress ahead of this time with the editor if you require more time to complete the revisions. Use the link below to submit your revision:

https://msb.msubmit.net/cgi-bin/main.plex

IMPORTANT: When you send your revision, we will require the following items:
1. the manuscript text in LaTeX, RTF or MS Word format
2. a letter with a detailed description of the changes made in response to the referees. Please specify clearly the exact places in the text (pages and paragraphs) where each change has been made in response to each specific comment given
3. three to four 'bullet points' highlighting the main findings of your study
4. a short 'blurb' text summarizing in two sentences the study (max. 250 characters)
5. a 'thumbnail image' (550px width and max 400px height, Illustrator, PowerPoint or jpeg format), which can be used as 'visual title' for the synopsis section of your paper.
6. Please include an author contributions statement after the Acknowledgements section (see https://www.embopress.org/page/journal/17444292/authorguide)
7. Please complete the CHECKLIST available at (https://bit.ly/EMBOPressAuthorChecklist).
Please note that the Author Checklist will be published alongside the paper as part of the transparent process (https://www.embopress.org/page/journal/17444292/authorguide#transparentprocess).
8. When assembling figures, please refer to our figure preparation guideline in order to ensure proper formatting and readability in print as well as on screen:
https://bit.ly/EMBOPressFigurePreparationGuideline
See also figure legend guidelines: https://www.embopress.org/page/journal/17444292/authorguide#figureformat
9. Please note that corresponding authors are required to supply an ORCID ID for their name upon submission of a revised manuscript (EMBO Press signed a joint statement to encourage ORCID adoption).
(https://www.embopress.org/page/journal/17444292/authorguide#editorialprocess)
Currently, our records indicate that the ORCID for your account is 0000-0002-5276-1392.

Please click the link below to modify this ORCID:
Link Not Available

The system will prompt you to fill in your funding and payment information. This will allow Wiley to send you a quote for the article processing charge (APC) in case of acceptance. This quote takes into account any reduction or fee waivers that you may be eligible for. Authors do not need to pay any fees before their manuscript is accepted and transferred to the publisher.

As a matter of course, please make sure that you have correctly followed the instructions for authors as given on the submission website.

*** PLEASE NOTE *** As part of the EMBO Press transparent editorial process initiative (see our Editorial at https://dx.doi.org/10.1038/msb.2010.72), Molecular Systems Biology publishes online a Review Process File with each accepted manuscripts. This file will be published in conjunction with your paper and will include the anonymous referee reports, your point-by-point response and all pertinent correspondence relating to the manuscript. If you do NOT want this File to be published, please inform the editorial office at msb@embo.org within 14 days upon receipt of the present letter.

Reviewer #1:

Study by Weber et al., on quantitative modeling of translational efficiency in Mycoplasma pneumoniae represents a good approach to quantify translational efficiency based on multiple experiments and data sets following tRNAs, mRNAs, proteins and ribosome density. The representation of data is clear, methods as well as experiments are described adequately and with enough clarity.
The study would benefit from the incorporation of the possible role of ribosome drop-off model in translational efficiency that authors disregarded in their study (recent works from Sin et al., 2016; Verma et al., 2019; Doerr et al., 2021; and previous work from Buckingham and Ehrenberg labs).This would strengthen the study and more importantly in the situation with the limited ribosome profiling reads would be additional explanation for changes in the efficiency of the protein synthesis. Data about +14nt peak in the ribosome profiling is not explained well and discussion on the effects of this peak on the efficiency is lacking(lines 751-757).

Reviewer #2:

In this paper the authors carry out ribosome profiling and mass spectrometry on Mycoplasma pneumoniae (Mpn) to estimate ribosome densities on transcripts and absolute translation initiation and elongation rates after integration with existing RNA-seq and other data. They show that on the whole previously demonstrated determinants of translation initiation rate and elongation speed do not seem to hold for this organism/data, with only small correlations to mRNA secondary structure near the initiation site and amino acid charge.

I found the study very interesting to read and commend the authors on hugely complex and important work. Moreover, the study connected its results well with existing literature and I found that it was mostly clear, although rather verbose in many areas. While the experimental approaches appear sound, or based on previously validated results, there were some areas where I was confused as to what the authors were trying to highlight or that they had processed the data in an appropriate way. This raised some worries that the confusing outcomes of the study could in fact be an artefact of the data processing. Some clarification on the steps performed and validity of assumptions would have helped.

In summary, this is a useful contribution to the field of translational control and will act as a valuable data set for future computational studies looking at how translation is regulated in diverse organisms. I also think the work will have broad appeal that is well aligned to the readership of Molecular Systems Biology. However, I do have some major concerns that need to be addressed:

1. Line 238: "The absolute numbers of mRNA molecules were estimated from the normalized read counts for six RNA-seq datasets of Mpn in exponential growth (Table S5) (Junier et al., 2016)". Typically, it is not possible to accurately estimate absolute mRNA counts by normalisation alone. Similar to the way the mass spec was performed, I would have expected some form of calibrant to be included. Could you explain why such a calibrant was not required and the level of confidence you have in your normalisation approach and why.

2. Line 255: I cannot understand why it is appropriate to assume that 80% of ribosomes would be translating in this organism. The references cited are nothing to do with this host and as mentioned throughout, it is likely that selective pressures for strong protein production (for fast replication) do not hold. Could this not explain some of the strange results you see?

3. Line 610: "...translation elongation rate adapts to the translation initiation rate in order to maintain an approximately constant ribosome density". While I agree that the analysis presented does indicate this, given that this deviates from all other examples of what is seen in other ribosome profiling data sets and that there is no clear mechanism that could underpin it, I'd want further experimental evidence that it is not in fact an artifact of the data processing carried out. This seems to be one of, if not the, major outcomes of the study, but the evidence is too dependent on assumptions and models that may not hold in this organism and which lead to strange implications (see next point).

4. Line 614: Related to the previous point, a translation rate of 59 codons/s sounds biophysically impossible to me, given measured speeds in other organisms. Again, given that translation rate is unlikely selected for in this organism, how is this possible? Given that the mass spec data is less likely to have issues as it relies on no assumptions, is this result not calling into question the densities and RNA levels that have been inferred?

5. There is no general meta-analysis of the densities across all genes to inspect the start and end of the coding regions (e.g., translational density averaged across all genes). It would be helpful to see that you recover the expected trinucleotide periodicity in the occupancy, with the correct frame being seen as peaks. I'd suggest that some more basic analysis of the ribosome profile data, similar to the original Ingolia paper to allow for a comparison.

6. When discussing possible reasons for the disconnect between the translation elongation dynamics and the common determinants it would have been good to include some discussion about the role of varying ribosome/tRNA concentrations and recycling (e.g. see: https://10.1098/rsif.2014.0589; https://doi.org/10.1016/j.cell.2010.02.036)

I also had some other smaller issues that need addressing:

- Abstract: You use the acronym "Mpn" at the end without defining.

- I found most of the figures difficult to read and understand due to tiny text, inconsistently labelled axes (e.g., units not always in parentheses), and difficult to follow layouts. Some careful reformatting and scaling would help significantly when reading the work. Also please ensure that the figures are visible when printed in greyscale where possible (e.g., Figure 6).

- The main text is far too verbose at present and needs to present the results in a more concise way. At present, it is hard work to read, and some comprehensive editing could help to improve accessibility.

Reviewer #3:

Summary:
========
The manuscript "Comprehensive quantitative modeling of translation efficiency in a genome-reduced bacterium" by Weber et al. is a true tour de force combining many different types of data (RNA-Seq, ribosome profiling, tRNA quantification, and proteomics) to provide an in depth and highly quantitative look at translation in the genome-reduced model bacterium Mycoplasma pneumoniae (Mpn). The main conclusions are that there seems to be little evidence for some of the determinants of translation efficiency identified in previous studies on other organisms to be active in Mpn and the surprising finding that elongation rates seem to be adapted to initiation rates in such a way that average ribosome density is constant across the transcriptome.

General remarks:
================
This is an extremely data rich manuscript. Its conclusions are well supported by the data presented. However, the main downside of the manuscript is its lack of conclusions. This kind of work has already been done one way or another for some of the more common model organisms. As such, the techniques themselves are not particularly innovative. So, then the question becomes is there something really new we learn in this organism that we did not know before? The most interesting point in this regard is the fact that elongation rate seems to be matched to initiation rate in order to provide a (more or less) constant ribosome density. This is surprising. But it seems very difficult to fathom how a cell could be doing this (especially if none of the "traditional" methods to control elongation seem to be at its disposal) and there is no real attempt to dig into possible mechanistic explanations (other than saying that it must have evolved that way). As such it remains an observation that will be the foundation for further work, but this is not a whole lot to carry a manuscript. The other main conclusion is the absence of evidence for determinants of translation efficiency known from other organisms. This is mostly a negative result and as such not that useful, especially since the absence of evidence is not the same as evidence for absence (see also below). In summary, while the work described here produced a treasure trove of data, the actual conclusions are sort of minimal and mostly negative. The most likely audience of this work will be the Mpn community, which might not be that interested in the specific conclusions but for which the data sets very carefully produced here could be extremely valuable.

Major points:
=============
1) The only real major point relates to the question of the lack of evidence for determinants of translation efficiency. The entire premise of the manuscript is that it looks for the first time with these kinds of extensive methods at a genome-reduced organism. While not said explicitly, the logic presumably is that a genome-reduced organism is going to be easier to understand since there are less "parts". But then, the statistical tests for most of the known (for other organisms) determinants of translation efficiency are negative (again, this is absence of evidence, not evidence of absence). Could it thus be that the fact that this organism has such a reduced set of genes is actually hurting the study because it reduces the statistical power? The authors should discuss this question in the manuscript (even though it somehow negates the entire premise of the study).
2) There is one issue concerning a minor conclusion in line 150: The authors claim that the absence of the non-explained peak at 14nt in the leaderless-like genes could be due to their smaller number. This reviewer does not understand this argument at all. Yes, if the number of genes is smaller, there is more noise that could mask a small peak. But that is not how the data looks like - the noise level for the leaderless genes is well low enough that the peak should be visible if it is there. Please clarify this argument.
3) Another smaller issue concerning conclusions is to explain in the paragraph in lines 349-362 how it was decided to when to call something "did not correlate" and when to call something a "weak ... correlation". After doing so, one probably will want to add the word "significant" in front of "correlation" in line 368.
4) Another potentially major point: It seems like a dangerous choice by the authors to reconstruct the position of the *first* nucleotide of the P-site from the ribosome profiling reads and then to quantify the codon-level occupancy by summing the ribosome counts at the three sub-codon positions of one codon. It seems that the most likely assignment error of the precise nucleotide would be an off-by-one-error. Thus, taking the convention that the ribosome position is quantified via the second P-site nucleotide and then summing over the three nucleotides of the codon (or equivalently staying with the assignment to the first

nucleotide of the P-site but then summing over the last nucleotide of the previous codon and the first two nucleotides of the current codon) would seem to be the safer choice. This reviewer realizes that this is something that happens at the very beginning of the analysis and that changing this convention would result in essentially the entire analysis of the manuscript and nearly every figure to have to be redone, but can the authors comment on this somewhat dangerous choice?

Minor points:
=============
While the manuscript is overall very well written, there are a few small points in which the presentation could be improved:
5) Would it be better to use a logarithmic y axis in figure S2B?
6) Unless this reviewer missed it, it does not look like figures S4B, S4D, or S4E are referenced in the manuscript.
7) line 473: "(B)" -> "(C)"
8) Why did the authors choose the average in lines 574 and 576 rather than the sum? It seems that the *total* amount of tRNA for an amino acid should correlate with the synthetase copy number, not their average.
9) line 1165: "mRNa" -> "mRNA"
10) the legend of Figure S19 does not seem to have much to do with what is actually shown in Figure S19
11) Please be mindful of significant digits. Do you really know the conversion coefficient in line 1258 to four digits? Do you think the experimental error on alpha and beta in lines 1359 and 1360 is one in a million? Do you really think your methods determine the total numbers of proteins in a cell to one part per million?
12) Please be careful with consistent notation. In some places the exponents appear in-line rather than superscripted (e.g., lines 222, 251, and 252). In many places e-notation (1e-4) is used rather than properly typesetting these numbers with proper powers of 10 (and the "e" is sometimes lower case and sometimes upper case), "p" switches between lower and upper case, and the temperature unit sometimes has a degree symbol and sometimes does not and sometimes has a space between the number and the symbol and sometimes does not. The authors should carefully go through the entire manuscript and supplemental material and make these notations consistent.

Point by point answer, manuscript “Comprehensive quantitative modeling of translation efficiency in a genome-reduced bacterium” MSB-2022-11301R

Notes

- All important changes in the revised manuscript have been highlighted in blue (font color).
- In this letter, all references to Figures of the manuscript or appendix refer to the figure numbering of the revised version.
- In this letter, all references to line numbers in the manuscript refer to the line numbers in the revised version.

Credentials for data access

Sequencing data is available at ArrayExpress and proteomics data at PRIDE. We share here the credentials for confidential access by peer-reviewers.

Proteomics data at PRIDE repository:
Title: Absolute protein quantification of Mycoplasma pneumoniae proteome by combining selected reaction monitoring of a reduced set of labeled peptides and label-free shotgun mass spectrometry.
Project accession: PXD035159
URL: https://www.ebi.ac.uk/pride/archive/projects/PXD035159
Username: reviewer_pxd035159@ebi.ac.uk
Password: 2hdHzyux

RNA-seq at ArrayExpress:
Title: tRNAs quantification by hydro-tRNA-seq in the genome-reduced bacterium Mycoplasma pneumoniae.
Accession: E-MTAB-11930
URL: https://www.ebi.ac.uk/biostudies/arrayexpress/studies/E-MTAB-11930
Username: Reviewer_E-MTAB-11930
Password: UUwcikwj

RNA-seq / ribo-seq at ArrayExpress:
Title: Ribosome profiling of the genome-reduced bacterium Mycoplasma.
pneumoniae
Accession: E-MTAB-11935
URL: https://www.ebi.ac.uk/biostudies/arrayexpress/studies/E-MTAB-11935
Username: Reviewer_E-MTAB-11935
Password: kago9sua

Reviewer #1 comments

Study by Weber et al., on quantitative modeling of translational efficiency in Mycoplasma pneumoniae represents a good approach to quantify translational efficiency based on multiple experiments and data sets following tRNAs, mRNAs, proteins and ribosome density. The representation of data is clear, methods as well as experiments are described adequately and with enough clarity.

The study would benefit from the incorporation of the possible role of ribosome drop-off model in translational efficiency that authors disregarded in their study (recent works from Sin et al., 2016; Verma et al., 2019; Doerr et al., 2021; and previous work from Buckingham and Ehrenberg labs).This would strengthen the study and more importantly in the situation with the limited ribosome profiling reads would be additional explanation for changes in the efficiency of the protein synthesis. Data about +14nt peak in the ribosome profiling is not explained well and discussion on the effects of this peak on the efficiency is lacking(lines 751-757).

We agree that ribosome drop-off could have an impact on the interpretation of our results. We have computed the ribosome drop-off in a similar way as in the work of (Sin, Chiarugi and Valleriani, 2016). The average ribosome density across genes in bins of 33 codons showed a decrease in the first 400 codons. By fitting the ribosome density profile to an exponential decay (Appendix Figure S9), we estimated a drop-off rate of 3.07e-05 $codon^{-1}$ for rep1 and 3.66e-05 $codon^{-1}$ for rep2, 7.2-fold lower compared to the average drop-off rate of 2.4e-04 $codon^{-1}$ observed in *E. coli* in normal growth condition (Sin et al. 2016). Thus, while ribosome drop-off probably has an impact on the distribution of ribosomes along mRNA sequences and can reduce the overall density of ribosomes, we think that its influence is limited. In particular, we would expect that ribosome drop-off would have a stronger impact on longer coding sequences. However, we did not find any correlation between CDS length and ribosome density (data not shown), suggesting that ribosome drop-off has not a large impact on longer CDSs in *M. pneumoniae*. Moreover, as mentioned in the main text (line 341), samples treated with chloramphenicol show an artifactual accumulation of ribosomes at the 5'-end of CDS (Mohammad, Green and Buskirk, 2019), because ribosomes continue initiating translation as the antibiotic inhibits elongation. Thus, the effect of ribosome drop-off cannot be fully distinguished from the potential biases induced by the use of chloramphenicol.

We have added one supplementary figure (Appendix Figure S9) and a paragraph in the Results section (lines 343-351) in order to clarify the role of ribosome drop-off in our study.

The presence of a secondary peak at +14nt derives from the observation of the metagene profile of ribosome occupancy, after applying the P-site offset to footprints, and corresponds to an accumulation of ribosomes with their P-site on the 5th codon. We have added one sentence in the results section to better explain the data (line 137). As mentioned in the main text, the origin of this secondary peak remains unexplained. The presence of this secondary peak had no effect on any of the downstream analyses, such as the estimation of the translation efficiency, neither at gene level nor at codon level, because the first 15 codons of CDSs were discarded in such analyses. We have also added one sentence in this respect in the Discussion section (lines 567-569).

Reviewer #2 comments

In this paper the authors carry out ribosome profiling and mass spectrometry on Mycoplasma pneumoniae (Mpn) to estimate ribosome densities on transcripts and absolute translation initiation and elongation rates after integration with existing RNA-seq and other data. They show that on the whole previously demonstrated determinants of translation initiation rate and elongation speed do not seem to hold for this organism/data, with only small correlations to mRNA secondary structure near the initiation site and amino acid charge.

I found the study very interesting to read and commend the authors on hugely complex and important work. Moreover, the study connected its results well with existing literature and I found that it was mostly clear, although rather verbose in many areas. While the experimental

approaches appear sound, or based on previously validated results, there were some areas where I was confused as to what the authors were trying to highlight or that they had processed the data in an appropriate way. This raised some worries that the confusing outcomes of the study could in fact be an artefact of the data processing. Some clarification on the steps performed and validity of assumptions would have helped.

In summary, this is a useful contribution to the field of translational control and will act as a valuable data set for future computational studies looking at how translation is regulated in diverse organisms. I also think the work will have broad appeal that is well aligned to the readership of Molecular Systems Biology. However, I do have some major concerns that need to be addressed:

1. Line 238: "The absolute numbers of mRNA molecules were estimated from the normalized read counts for six RNA-seq datasets of Mpn in exponential growth (Table S5) (Junier et al., 2016)". Typically, it is not possible to accurately estimate absolute mRNA counts by normalisation alone. Similar to the way the mass spec was performed, I would have expected some form of calibrant to be included. Could you explain why such a calibrant was not required and the level of confidence you have in your normalisation approach and why.

We estimated the absolute number of mRNA molecules based on one unique reference: the number of ribosomal RNA molecules per cell. We assumed that the normalized read count of the 23S rRNA was a good estimate of the relative number of molecules of rRNA per cell, relative to the total cellular RNA. Due to the complexity of the ribosome biogenesis process, including the maturation of the rRNAs and the assembly of ribosomal proteins (Davis and Williamson, 2017), the exact fraction of rRNA in complex with mature ribosomes is difficult to estimate. Biophysical simulations of the 30S ribosomal subunit assembly in *E. coli* suggested that the abundance of free rRNA and intermediates are two to three orders of magnitude smaller than the average number of assembled 30S subunits (Earnest *et al.*, 2015). We assumed that 90% of the rRNA was bound to the ribosome. Using the absolute number of ribosomes per cell determined by separate methods (cryo electron tomography, (O'Reilly *et al.*, 2020; Xue *et al.*, 2022) and mass spectrometry quantification of ribosomal proteins (Gerber *et al.*, 2003)), we derived the conversion factor from the RNA-seq normalized read counts to the absolute number of RNA molecules. Thus, our calibration method does not depend on the specific normalization method used for RNA-seq read counts, but rather on the estimation of the number of ribosomes per cell, as well as on the underlying assumptions on the relationship between the number of ribosomes and the read count of the 23S rRNA. While this calibration is based on only one RNA species, it benefits from the robust estimation of the number of ribosomes per cell, determined by two independent experimental methods: cryo-EM (direct counting) and mass spectrometry (calibrated by labeled peptides), both resulting in an estimation of around 300 ribosomes per cell. We have added one sentence in the manuscript (lines 201-203).

As mentioned in the manuscript, the concentration of mRNAs in Mpn is of the same order of magnitude as the concentration of mRNAs in *E. coli*, although approximately three times smaller. In rich medium, each *E. coli* cell contains ~8000 transcripts (Bartholomäus *et al.*, 2016; Gorochowski *et al.*, 2019) in a volume of 4.4 μm3 (Volkmer and Heinemann, 2011). Taking into account that the cellular volume of Mpn is 59 times smaller (0.075 μm3), we would expect 136 mRNA molecules in Mpn for the same cellular concentration. We found a total of 46.5 mRNA molecules, which could be explained by the slower metabolism and transcription rate of Mpn compared to fast-growing bacterial species such as *E. coli* (8 hours versus 30 min cell doubling time). A previous study estimated the absolute total number of mRNA molecules in Mpn at a similar order of magnitude although lower (around 10 molecules per cell) by using tiling arrays and 12 RNA spike-ins (Maier *et al.*, 2011). However, the authors based their calculation on an underestimation of 150 ribosomes per cell. The estimation presented in this manuscript is based on the more reliable RNA-seq technology and a more precise and updated estimation of the number of ribosomes per cell. We have modified the main text to describe this comparison better (lines 208-209).

2. Line 255: I cannot understand why it is appropriate to assume that 80% of ribosomes would be translating in this organism. The references cited are nothing to do with this host and as

mentioned throughout, it is likely that selective pressures for strong protein production (for fast replication) do not hold. Could this not explain some of the strange results you see?

First, we would like to clarify that this assumption was only used to determine the conversion factor between relative ribosome density and absolute ribosome densities (in ribosome per codon). This conversion factor, in turn, was used to determine the absolute scale of the final values of translation efficiency and elongation rates. Second, we must emphasize that the conversion factor is based on the absolute copy number of ribosomes that are actively translating, but does not depend on the total number of ribosomes in the cell.

As mentioned in the main text, the total number of ribosomes was determined by two means. First, the total number of ribosomes was estimated by the average ribosomal protein copy number determined by a combination of labeled and label-free mass spectrometry. This number includes, in theory, all states of the 70S ribosomes, 50S and 30S subunits, and free ribosomal proteins, and resulted in an estimate of around 300 copies per cell. Second, the total number of ribosomes was estimated based on the results of the latest cryo-EM studies in Mpn (O'Reilly *et al.*, 2020; Xue *et al.*, 2022), which report a total number of 50S and 70S ribosomes of approximately 300-500 per cell. Because of the limited resolution of the tomograms, complexes of a size similar or smaller than the 50S subunit are difficult to identify, and correlation analyses suggest that about 90% of 70S were identified, while only 50% of the 50S could be captured in such experiments. In the most recent cryo-EM study (Xue *et al.*, 2022), the total number of sub-tomograms (24,157) identified as free 50S subunits was 3.2 times smaller than the number of sub-tomograms identified as 70S ribosomes (77,539). Thus, the quantification of the number of biomolecules by Cryo-EM is reliable for the 70S ribosomes, and a large proportion of these 300-500 correspond to 70S ribosomes. Importantly, in the mentioned study, all of the identified 70S ribosomes were classified in one of the structures corresponding to the elongation cycle phases, bound to a mRNA molecule, indicating that all of the 70S ribosomes identified were actively elongating. We conclude from these observations that a large proportion of the 300-500 identified ribosomes correspond to 70S ribosomes in an active elongation state. While the resolution of cryo-EM studies does not allow the detection of 50S and 30S ribosomal subunits in the free state, it does give a lower bound estimate of the number of actively translating 70S ribosomes in Mpn. Importantly, this estimate is based on the latest cryo-EM study in Mycoplasma pneumoniae (Xue *et al.*, 2022), and not in a different host.

We have modified the main text in order to clarify our estimation (lines 211-213).

3. Line 610: "...translation elongation rate adapts to the translation initiation rate in order to maintain an approximately constant ribosome density". While I agree that the analysis presented does indicate this, given that this deviates from all other examples of what is seen in other ribosome profiling data sets and that there is no clear mechanism that could underpin it,

I'd want further experimental evidence that it is not in fact an artifact of the data processing carried out. This seems to be one of, if not the, major outcomes of the study, but the evidence is too dependent on assumptions and models that may not hold in this organism and which lead to strange implications (see next point).

We agree with the reviewer that this hypothesis is interesting but that it lacks experimental evidence. To further explore this and assess the interplay between translation initiation and elongation rates in Mpn, we performed an additional exploratory experiment based on the construction of gene chimeras aimed to modify the translation initiation rate of a specific gene, while keeping the same coding sequence that is supposed to determine the elongation rate. Since translation initiation depends on the 5'UTR and the 5' of the coding sequences, we swapped the 5'UTR and/or N-terminal sequences (Figure 1 in this letter, see below) of pairs of endogenous genes predicted with a very low (gene B) and very high (gene A) translation efficiencies (see Table 1 in this letter for the criteria of selection).

We reasoned that increasing the initiation rate (k_{ini}) of gene B should lead to two possible outcomes. If the elongation rate adapts to the initiation rate, the translation efficiency will increase as much as k_{ini} increases. If the elongation rate does not adapt and remains constant, the translation efficiency will be limited by k_{elong}. In this case, we expect that for a gene with an elongation rate among the smallest observed in our dataset (in the order of 1 codon s^{-1}), a moderate increase in the initiation rate may lead to ribosome collisions and limit translation efficiency. In contrast, we expect that the rate of initiation of gene A decreases when replacing the 5' sequences with elements of gene B. In this case, initiation will be the limiting step, such that any change in elongation rate, whether it adapts and becomes slower, or stays the same, should not significantly influence the protein output.

In Figure 2 of this letter (see below) we show the results of this experiment, in which the protein to mRNA ratio (as a proxy for translation efficiency) of two gene pairs was quantified by western blot and qRT-PCR from wild-type Mpn strains expressing the specific constructs shown in Figure 1.

gene type	gene name	function	protein length (a.a.)	mRNA copy number	protein copy number	protein half-life (h)	translation initiation rate (s^{-1})	ribosome density ($codon^{-1}$)	translation elongation rate (s^{-1})
A	MPN401	Transcription elongation factor greA (Transcript cleavage factor greA).	161	0.083	1372	10	0.67	0.017	40.60
B	MPN406	Acyl carrier protein homolog	85	0.135	86	49	0.02	0.119	0.14
A	MPN547	Fatty acid kinase (FAK kinase)	559	0.028	270	55	0.25	0.017	15.20
B	MPN017	Methylenetetrahydrofolate dehydrogenase (EC 1.5.1.5)	270	0.023	15	18	0.02	0.019	1.10

Table 1: Selected pairs of endogenous genes. Features taken into consideration for the selected pairs were: i) Genes of type A were selected for their high initiation and high elongation rates (ratio k^{abs}_{TE}/ribo density), and genes of type B were selected for their low/moderate initiation rate and low elongation rate. The largest difference should be in the elongation rate between gene A and gene B. ii) Not a membrane protein. iii) Not a member of a known protein complex. iv) Similar gene length. v) First gene in operon (leader gene), as in the case of non-leader genes, the translational coupling could have a stronger impact on translation elongation compared to other mechanisms and would be a confounding factor. vi) Raw count of ribosomes > 100, to limit noise in the estimation of the ribosome density and elongation rate (ratio k^{abs}_{TE}/ribo density). vii) Protein half-life >~ 10h. viii) protein copy number >~ 20, in order to detect it by western.

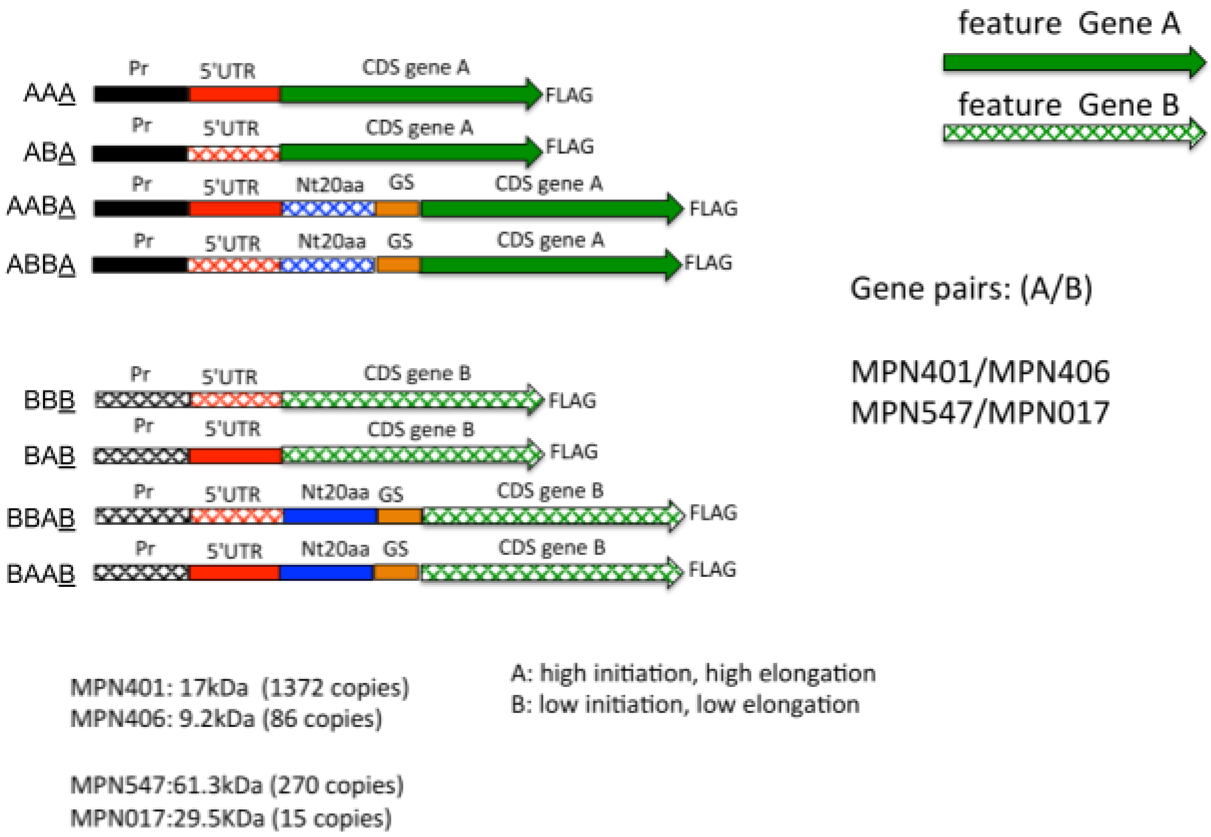

Figure 1: Design of the gene constructs for the translation elongation experiment. For each gene pair A/B, 8 constructs were designed, as described in the text. The construct names (AAA, ABA, AABA, ABBA, BBB, BAB, BBAB, BAAB) indicate the 5’ elements from gene A or gene B (promoter, 5’UTR, N-terminal fusion of 5’ of coding sequence, and wild-type CDS).

In the case of the MPN401(A)/MPN406(B) gene pair (Figure 2B), we observed as expected a clear ~3-fold increase in translation efficiency in the gene B construct when swapping the 5’UTR with the one of the gene A (construct BAB compared to BBB). A similar and opposite change was observed for gene A when swapping the 5’UTR (construct ABA compared to AAA), with a ~4.5-fold decrease in translation efficiency. However, the change in initiation rate was not as large as expected, compared to the 30-fold difference in initiation rate for endogenous genes in our dataset. These results suggest that the slow elongation rate of gene B, 0.14 s^{-1}, is not a limiting factor for the increase in translation efficiency observed for gene B. Unfortunately, protein expression was too low to be detected for the BBAB and BAAB constructs.

In the case of the MPN547(A)/MPN017(B) gene pair (Figure 2B), the swapping of the 5'UTR for gene B resulted in a 2.8-fold decrease of translation efficiency (construct BAB compared to BBB), instead of the expected increase. A similar 1.9-fold decrease was observed for the N-terminal fusion of gene B with the N-terminal of gene A (construct BBAB compared to BAAB). The translation efficiency of gene A, when swapping the 5'UTR of gene B, remained almost constant (1.2-fold increase). The same was observed for the N-terminal fusion of gene A (1.3-fold increase). Thus, in this case, the efficiency of the 5' of gene A on translation initiation rate seemed to be lower than the 5' of gene B, suggesting that other factors contribute to the final protein output, as can be the influence of the chimera sequences in mRNA and protein stability.

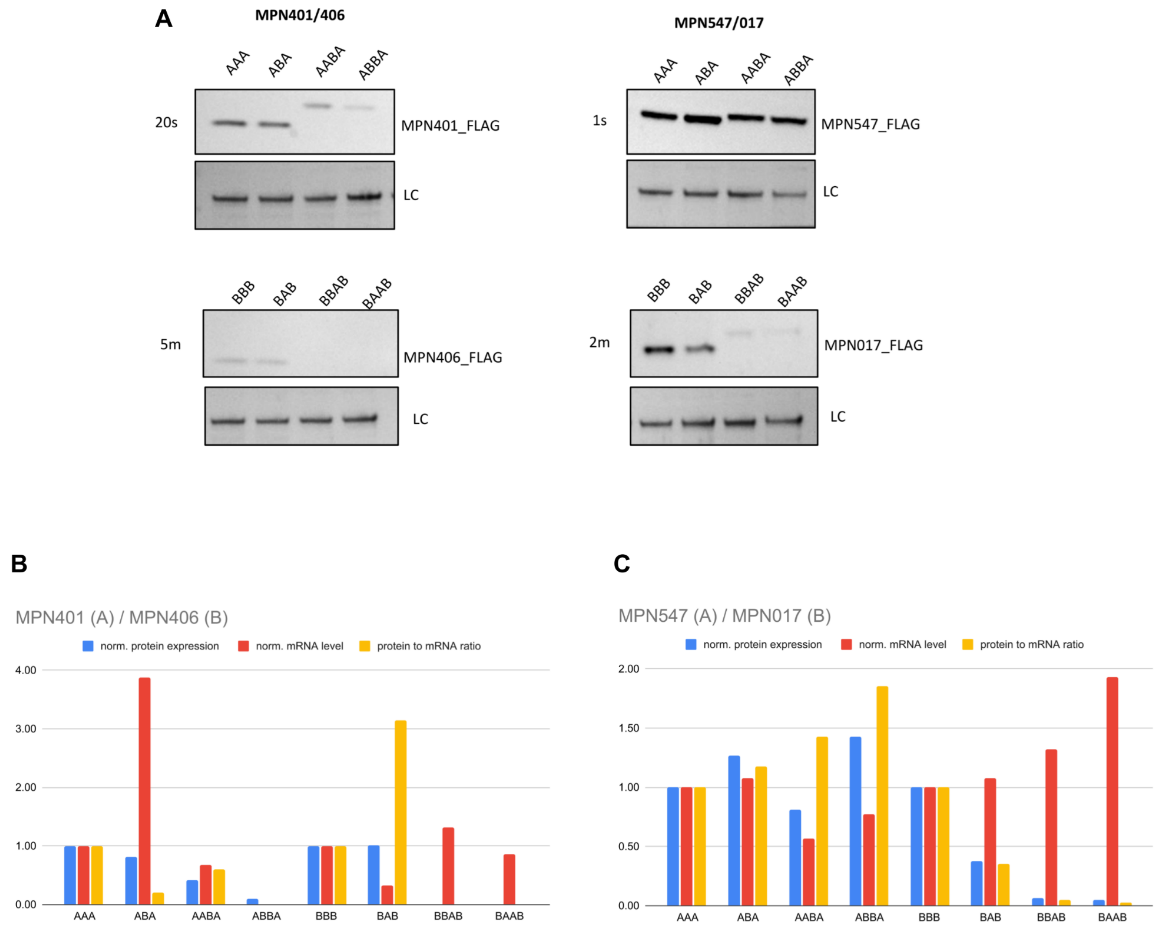

Figure 2: Results of the protein and mRNA quantification. (A) Expression of the FLAG-tagged variant of protein A or B was assessed by Western blot analysis using anti-FLAG antibody. LC, loading control. Normalized protein abundance quantified by Western Blot, normalized mRNA abundance quantified by qPCR, and normalized protein to mRNA ratio for the (B) MPN401 / MPN406 gene pair, and the (C) MPN547 / MPN017 gene pair.

Although the experiment described above showed the importance of the 5'UTR regions modulating transcription and translation, unfortunately the assay turned inconclusive, suggesting that a larger number of gene pairs should be analyzed to capture the overall complexity. In addition, the outcome measure of this approach is only an indirect method to estimate elongation rates, and more specific techniques such as ribosome profiling would be required. However, this is difficult to implement in chimera constructs without replacing first the respective endogenous genes from the genome, which is technically difficult in Mpn. Although alternative methods to measure elongation rates may be developed, we believe that this would require extensive research that cannot be achieved during this revision. Therefore, due to these reasons and the fact that the current manuscript is already highly complex, we strongly believe that additional experiments to prove and validate the hypothesis that translation elongation rate adapts to translation initiation should be the focus of an independent follow up study. Accordingly, we have toned down our claims about this hypothesis in the Results section (lines 473-475), removed it from the abstract, and we have extended its interpretation in the Discussion section (lines 689-708). In particular, we have described in the Discussion the potential artifacts, both at the level of our modeling approach and at the level of experimental biases, that could explain the observed correlation between elongation and initiation rates. We think that the observation that the gene average elongation rate is highly correlated to the initiation rate is still a robust and valid result, but that our current data cannot bring any insight into the underlying mechanism.

4. Line 614: Related to the previous point, a translation rate of 59 codons/s sounds biophysically impossible to me, given measured speeds in other organisms. Again, given that translation rate is unlikely selected for in this organism, how is this possible? Given that the mass spec data is less likely to have issues as it relies on no assumptions, is this result not calling into question the densities and RNA levels that have been inferred?

We agree with the reviewer that a translational elongation rate of 59 codons/s sounds biophysically unlikely. However, we argue that 1) we cannot rule out that the translation elongation rate for specific genes in Mpn could be as high as in other bacteria (>20 aa/s), and 2) the calculated maximum elongation rate in Mpn could be due to outlier genes that suffered from strong biases in the absolute protein quantification.

The elongation speed in *E. coli*, which is a fast-growing bacteria, ranges from 8 to 18 codons/s depending on the growth medium (Dai *et al.*, 2016; Zhu *et al.*, 2019). However, the latter studies measured the translation rate using one specific gene, lacZ, using the β-galactosidase assay and pulse-chase assays, such that the elongation rates for the other genes is not known. Moreover, indirect calculations based on the amount of total protein per cell, the number of ribosomes per cell, the percentage of active ribosomes (85%), and the cell growth rate, suggest that the global elongation rate can be as high as 22 codons/s (Bremer and Dennis, 2008). Thus, we can expect the elongation rate to be even higher for certain highly expressed genes with optimal codons in *E. coli*. Interestingly, a study in yeast showed that the elongation rate varies up to ~20-fold among transcripts (Riba *et al.*, 2019). Although the global average translation rate is unlikely to be under strong selective pressure in Mpn, a selective pressure might still exist for some specific genes.

The most probable explanation for the unlikely maximum elongation rate we observed is the experimental variability and biases in the quantification of protein abundance. While the absolute quantification by labeled mass spectrometry does not rely on any of the specific assumptions of our modeling approach, this technique does suffer from inherent biases and variability. The protein with the highest calculated elongation rate of 59 codons/s, MPN562, showed one of the largest deviations from the calibration curve. The TOP3 intensity for this protein was much lower than expected given its absolute abundance measured by labeled peptide, for all the samples (Figure 3 in this letter). This observation suggests that the absolute quantification of this protein might have suffered from an undetermined experimental error.

The use of stable isotopically-labeled tryptic peptides as an internal standard can be biased by differences in protein digestion and upstream processes during sample preparation (Oeckl, Steinacker and Otto, 2018) between the labeled peptide of the targeted protein. The digestion, as well as the degradation (e.g. deamidation) of the peptide, can differ substantially between the intact targeted protein and the labeled peptide. A previous study comparing different internal standard approaches for monoclonal antibodies quantification showed biases in absolute quantification of up to 60% when using a single labeled tryptic peptide (Li *et al.*, 2012). The TOP3 intensity for the proteins with the second, third, and forth highest elongation rates, MPN134, MPN674, MPN250, did not show such a strong deviation from the calibration curve, although this does not rule out possible biases in the absolute quantification.

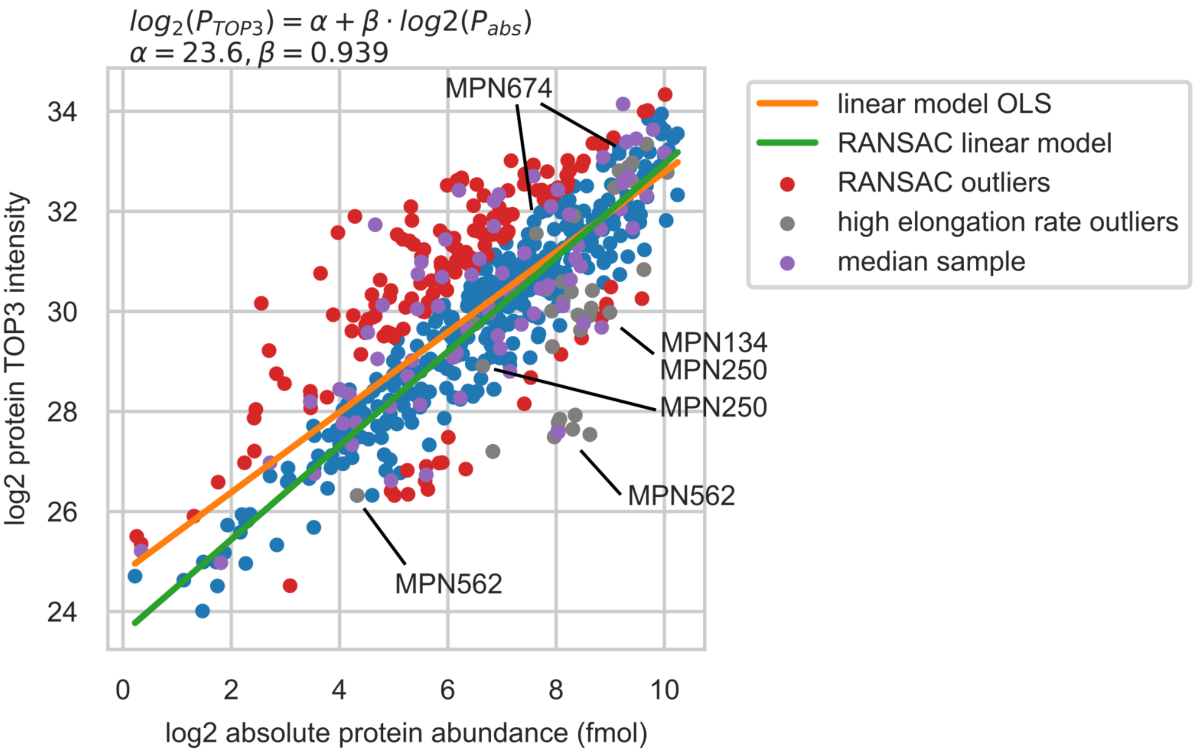

Figure 3: Calibration curve for absolute quantification of proteins (AQUA) highlighting genes with extremely high elongation rate. A robust linear regression (same data as in Appendix Figure S22) using the RANSAC method was performed on the absolute amount of each peptide of the SRM assay (Pabs) and the protein top 3 intensity of the Orbitrap (PTOP3), using all peptide values for all replicates and the three growth time points. The gene with highest calculated elongation rate (ratio $k_{TE}^{abs}/\langle\rho\rangle$), MPN562, appears as an outlier, with TOP3 intensities in all replicates much smaller than expected from the absolute abundance determined by labeled peptide.

Based on these observations, the most probable explanation for the observed extreme values of the elongation rates are the biases in protein abundance quantification. In order to take into account this variability, we have changed the description of the elongation rates in the main text (lines 453-455) and reported the values of the 5th and 95th percentiles, which span a 40-fold range from 0.687 codon/s to 18.6 codon/s for labeled proteins. Interestingly, this range was very similar when considering all proteins, labeled and label-free (0.465 to 18.7 codon/s). We have also added an explanation on the possible biases leading to extreme values of elongation rates in the Discussion section (lines 680-687).

5. There is no general meta-analysis of the densities across all genes to inspect the start and end of the coding regions (e.g., translational density averaged across all genes). It would be helpful to see that you recover the expected trinucleotide periodicity in the occupancy, with the

correct frame being seen as peaks. I'd suggest that some more basic analysis of the ribosome profile data, similar to the original Ingolia paper to allow for a comparison.

The meta-analysis of the normalized ribosome counts across all genes at the start of coding regions is presented in Figure 1B, which we called “metagene profile”, both for tetracycline-treated and chloramphenicol-treated samples.

One of the hallmarks of the ribosome profiling signal has been the three nucleotides periodicity, as observed in yeast, where more than 75% of the reads align to the first nucleotide of codons (Mangkalaphiban *et al.*, 2021), reflecting the elongation dynamics of the ribosome. In *E. coli*, however, the signal periodicity is much weaker, with approximately 40% of reads aligning to the first frame (Hwang and Buskirk, 2017). This weak periodicity has been attributed to the sequence specificity of the MNase, which cleaves preferentially before A and T residues (Dingwall, Lomonossoff and Laskey, 1981). Because A and T are found more frequently at the second nucleotide of codons in the *E. coli* genome, most footprints have their 3’ end aligned to the first sub-codon position (Hwang and Buskirk, 2017). We observed the same nucleotide enrichment in Mpn, where the second sub-codon position showed a higher fraction of A and T compared to the first and third (Fisher exact test two-sided, $p \cong 0$ in both cases) sub-codon positions (Appendix Figure S20). After applying a fixed offset of 19 nt from the 3’ end of footprints of length 20 to 34 nt, the P-site of the ribosome occurred more frequently at the third sub-codon position (median occurrence for the different footprint lengths: 26.4% in frame 1, 31.7% in frame 2 and 40.1% in frame 3) (Appendix Figure S21). In general, these results suggested that the weak periodicity in the ribosome signal in Mpn was mainly driven by the aforementioned biases. Importantly, a strong 3 nt periodicity in the ribosome occupancy signal is not expected to be found in bacterial studies using MNase.

We have added a paragraph in the Methods section called “Periodicity in ribosome profiling signal” to describe these observations (lines 975-991). We have also added a brief explanation at the end of the Results section “Ribosome profiling in Mycoplasma pneumoniae” (lines 171-173).

6. When discussing possible reasons for the disconnect between the translation elongation dynamics and the common determinants it would have been good to include some discussion about the role of varying ribosome/tRNA concentrations and recycling (e.g. see: https://10.1098/rsif.2014.0589; https://doi.org/10.1016/j.cell.2010.02.036)

We have added the following sentences in the Discussion (lines 724-728): “Synonymous codon order has also been suggested to influence the elongation speed. The putative recycling of tRNAs in the vicinity of the ribosome may lead to faster local elongation rate for subsequent occurrences of codons that use the same tRNA (Cannarozzi *et al.*, 2010). This effect, however, remains difficult to isolate from other effects in the statistical analysis of endogenous genes.”

I also had some other smaller issues that need addressing:

- Abstract: You use the acronym "Mpn" at the end without defining.

We have included the acronym definition in the abstract.

- I found most of the figures difficult to read and understand due to tiny text, inconsistently labelled axes (e.g., units not always in parentheses), and difficult to follow layouts. Some careful reformatting and scaling would help significantly when reading the work. Also please ensure that the figures are visible when printed in greyscale where possible (e.g., Figure 6).

We have increased the font size of the labels in most of the figures whenever possible, corrected the axes labeling, and tried to improve the readability of the figures layout. We have also modified Figure 6 to allow for better readability in greyscale, by using distinct marker shapes for labeled and not labeled proteins.

- The main text is far too verbose at present and needs to present the results in a more concise way. At present, it is hard work to read, and some comprehensive editing could help to improve accessibility.

We have streamlined the manuscript and tried to present the results in a more concise way.

Reviewer #3 comments

Summary:
========
The manuscript "Comprehensive quantitative modeling of translation efficiency in a genome-reduced bacterium" by Weber et al. is a true tour de force combining many different types of data (RNA-Seq, ribosome profiling, tRNA quantification, and proteomics) to provide an in depth and highly quantitative look at translation in the genome-reduced model bacterium Mycoplasma pneumoniae (Mpn). The main conclusions are that there seems to be little evidence for some of the determinants of translation efficiency identified in previous studies on other organisms to be active in Mpn and the surprising finding that elongation rates seem to be adapted to initiation rates in such a way that average ribosome density is constant across the transcriptome.

General remarks:
================

This is an extremely data rich manuscript. Its conclusions are well supported by the data presented. However, the main downside of the manuscript is its lack of conclusions. This kind of work has already been done one way or another for some of the more common model organisms. As such, the techniques themselves are not particularly innovative. So, then the question becomes is there something really new we learn in this organism that we did not know before? The most interesting point in this regard is the fact that elongation rate seems to be matched to initiation rate in order to provide a (more or less) constant ribosome density. This is surprising. But it seems very difficult to fathom how a cell could be doing this (especially if none of the "traditional" methods to control elongation seem to be at its disposal) and there is no real attempt to dig into possible mechanistic explanations (other than saying that it must have evolved that way). As such it remains an observation that will be the foundation for further work, but this is not a whole lot to carry a manuscript. The other main conclusion is the absence of evidence for determinants of translation efficiency known from other organisms. This is mostly a negative result and as such not that useful, especially since the absence of evidence is not the same as evidence for absence (see also below). In summary, while the work described here produced a treasure trove of data, the actual conclusions are sort of minimal and mostly negative. The most likely audience of this work will be the Mpn community, which might not be that interested in the specific conclusions but for which the data sets very carefully produced here could be extremely valuable.

It is true that globally speaking we did not find any new mechanisms to regulate translational efficiency. But it is also true that a fully comprehensive analysis of translation including RNA and protein copy numbers, tRNA copy numbers, protein and RNA half lifes, ribosome profiling, and a mathematical model incorporating all these data obtained by the same group under the same conditions and strain, to the best of our knowledge, has not been done. We believe that comparing ribosome profiling data to the absolute translation efficiencies derived from protein, mRNA and protein half-life offers a unique framework to test quantitatively the consistency of different measurements. Moreover, using a genome-reduced organism makes it easier to discard alternative hypotheses.

Thus we believe that our study represents one of the most comprehensive analyses in a bacterium, and makes credible the fact of the existence of unidentified mechanisms of translational regulation which is more difficult to affirm from other less comprehensive studies or more complex organisms.

On the other hand, we agree with the reviewer that the hypothesis of the adaptation of the translation elongation rate to the initiation rate is interesting but that it lacks experimental evidence. Interestingly, a recent study in the filamentous fungus *Neurospora crassa* (Lyu *et al.*, 2021) showed that translation of mRNAs with long CDS and non-optimal codon usage preferentially induces phosphorylation of initiation factor eIF2α, which inhibits translation initiation efficiency, indicating a feedback mechanism from translation elongation to control translation initiation. Such a feedback mechanism is believed to have evolved such as to avoid ribosome density saturation and ribosome collisions, which would negatively impact the expression of these highly abundant proteins. How such a putative feedback mechanism could occur in Mpn remains unknown.

To assess the interplay between translation initiation and elongation rates in Mpn, we performed an additional exploratory experiment based on the construction of gene chimeras aimed to modify the translation initiation rate of a specific gene, while keeping the same coding sequence that is supposed to determine the elongation rate. This experiment and its results are described in the answer to reviewer #2, major point #3. Unfortunately this assay turned inconclusive, suggesting that more specific techniques such as ribosome profiling of synthetic gene libraries would be required to further assess this hypothesis. We believe that this would require extensive research that cannot be achieved during this revision. Therefore, due to these reasons and the fact that the current manuscript is already highly complex, we strongly believe that additional experiments to prove and validate the hypothesis that translation elongation rate adapts to translation initiation should be the focus of an independent follow up study. Accordingly, we have toned down our claims about this hypothesis in the Results section (lines 473-475), removed it from the abstract, and we have extended its interpretation in the Discussion section (lines 689-708). In particular, we have described in the Discussion the potential artifacts, both at the level of our modeling approach and at the level of experimental biases, that could explain the observed correlation between elongation and initiation rates. We think that the observation that the gene average elongation rate is highly correlated to the initiation rate is still a robust and valid result, but that our current data cannot bring any insight into the underlying mechanism.

Major points:
=============
1) The only real major point relates to the question of the lack of evidence for determinants of translation efficiency. The entire premise of the manuscript is that it looks for the first time with these kinds of extensive methods at a genome-reduced organism. While not said explicitly, the logic presumably is that a genome-reduced organism is going to be easier to understand since there are less "parts". But then, the statistical tests for most of the known (for other organisms) determinants of translation efficiency are negative (again, this is absence of evidence, not evidence of absence). Could it thus be that the fact that this organism has such a reduced set of genes is actually hurting the study because it reduces the statistical power? The authors should

discuss this question in the manuscript (even though it somehow negates the entire premise of the study).

We agree with the reviewer that the negative results in the statistical tests only demonstrate an absence of evidence, and not evidence for absence of determinants. The statistical power of our study depends mainly on four factors: i) the number of observations (genes or codon positions), ii) the effect size of the determinants, iii) the total variance of the translation efficiency / elongation rate and iv) the noise in the data. These aspects determine the statistical power of the two main analyses presented in the manuscript: determinants of the absolute translation efficiency at the gene level, and determinants of the local elongation rate at the codon level.

At the gene level, the number of genes in Mpn is smaller than in other organisms (~700 compared to ~4600 in *E. coli* and ~7600 in yeast). However, in the only two studies which quantified the absolute translation efficiency in yeast, the number of quantified genes only represented a subset of all genes (1,117 (Lahtvee *et al.*, 2017) and 1106 (Riba *et al.*, 2019)). In our study, the proportion of genes for which we quantified the absolute translation efficiency was higher, 67% compared to 14%. Thus, the number of genes used for the analysis of determinants in our study was only 2.3 times smaller (475 genes) compared to the other studies in yeast (~1110 genes). The smaller number of genes reduces statistical power, but the difference with similar studies in yeast should be moderate.

The effect size of a determinant, i.e. the strength of the correlation between a determinant and the translation efficiency, is not known a priori and can be different in Mpn compared to other organisms. The evolutionary process of genome reduction may have favored certain mechanisms of translational control in Mpn, while suppressing others. For example, the strength of the Shine-Dalgarno motif can modulate more than 200-fold the translation initiation rate in *E. coli* (Ding *et al.*, 2020). Such a large effect size would have been detected in our dataset, even with a reduced statistical power. Instead, our results did not show any correlation between the strength of the Shine-Dalgarno motif and translation efficiency, showing that the influence of this determinant is much smaller in Mpn compared to *E. coli*. However, the effect sizes of the other determinants in Mpn remain difficult to predict.

The total variance of the translation efficiency and the level of noise in the data also have an important impact on statistical power. First, a larger total variation in the true values of translation efficiency, which could be due to the existence of some determinants with a strong influence, will increase the probability to detect such determinants. In our study, the values of the absolute translation efficiency varied over 160-fold, a range similar to the variation measured in yeast (between ~100-fold (Riba *et al.*, 2019) and 400-fold (Lahtvee *et al.*, 2017)). Second, a low level of noise in the measurement of translation efficiency will increase the probability to detect the effect of determinants. The major factor controlling noise in the absolute translation efficiency is the variability in protein

abundance, as mentioned in the manuscript. We expect this variability to be comparable to measurements based on mass spectrometry using labeled biomass as an internal standard (Lahtvee *et al.*, 2017).

Thus, in the case of the analysis of the determinants of the absolute translation efficiency, we conclude that the statistical power of our study is of the same order of magnitude as similar studies in yeast. However, we cannot rule out that some of these determinants may have an effect size smaller than the minimum effect size detectable with our study.

In the case of the analysis of local elongation rate, the number of observations is much larger (240k codons) but the noise in the ribosome density data is also higher. The total size of the coding genome in Mpn (~0.6M nt) is 6.5 times smaller than in *E. coli* (4.1M nt), and 14 times smaller than in yeast (~9M nt) (Nagalakshmi *et al.*, 2008), which could reduce the statistical power for the analysis of the determinants of local elongation rate. To this regard, sequencing depth of the ribosome footprints library is one of the major factors controlling noise. In our study, the average number of footprint reads after filtering per codon in the transcriptome was 7.6 (rep1) and 2.7 (rep2), which was similar to other studies in yeast (~0.1-90 RPFs per codon (Diament and Tuller, 2016)) and in *E. coli* (~5-10 RPFs per codon (Mohammad, Green and Buskirk, 2019)). Thus, in the case of the analysis of determinants of local elongation rates, the statistical power of our study is one order of magnitude smaller compared to similar studies in *E. coli* and in yeast. Nevertheless, we found that determinants such as the codon identity at the A-site and Shine-Dalgarno-like motif had a small but significant impact on translation elongation.

We have added a new section called “Limitations of the study” in the Discussion section (lines 582-599).

2) There is one issue concerning a minor conclusion in line 150: The authors claim that the absence of the non-explained peak at 14nt in the leaderless-like genes could be due to their smaller number. This reviewer does not understand this argument at all. Yes, if the number of genes is smaller, there is more noise that could mask a small peak. But that is not how the data looks like - the noise level for the leaderless genes is well low enough that the peak should be visible if it is there. Please clarify this argument.

The noise in the metagene profile of leaderless-like genes was indeed larger compared to the noise in the profile of leadered gene, due to the smaller number of genes (Figure 4 in this letter). The aggregated number of ribosome counts along the CDS is ~8-fold higher for leadered genes compared to leaderless-like genes. However, we agree with the reviewer that the noise level for the leaderless-like genes is clearly not enough to mask a peak similar to the one observed at position 0 for the leadered genes, where the peak height is ~12-fold the average ribosome count along the CDS. A peak with a similar intensity would reach ~55 ribosome counts in the profile of leaderless-like genes (Figure 3B in this letter). Thus, for leaderless-like genes, we do not observe any peak with such

an intensity, neither at position 0, nor at position +14 nt. We have corrected this observation in the main text (line 140). We have also added one sentence in the discussion about the secondary peak at +14nt, following comments by reviewer #1 (lines 567-569).

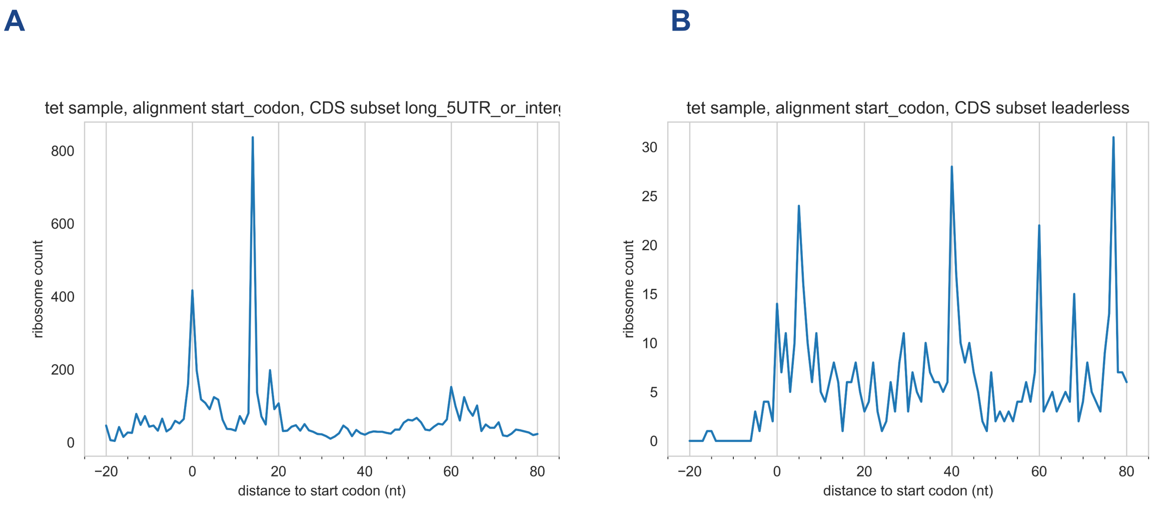

Figure 4. Metagene profile for leadered and leaderless-like genes.
Metagene profile in absolute ribosome counts aligned to the first nucleotide of the start codon of CDSs, tetracycline-treated sample, for (A) leadered (length of 5'UTR > 8) genes (N=254) and (B) leaderless-like (length of 5'UTR <= 8) genes (N=51).

3) Another smaller issue concerning conclusions is to explain in the paragraph in lines 349-362 how it was decided to when to call something "did not correlate" and when to call something a "weak ... correlation". After doing so, one probably will want to add the word "significant" in front of "correlation" in line 368.

We have now added calculations of two-sided statistical tests for the majority of Pearson correlation coefficients in the manuscript. In general, we considered as significant a Pearson correlation coefficient when $|r| > 0.2$ and $p < 0.01$. We have clarified this definition throughout the last three paragraphs of the Results section "Determinants of translation initiation rates".

*4) Another potentially major point: It seems like a dangerous choice by the authors to reconstruct the position of the *first* nucleotide of the P-site from the ribosome profiling reads and then to quantify the codon-level occupancy by summing the ribosome counts at the three sub-codon positions of one codon. It seems that the most likely assignment error of the precise nucleotide would be an off-by-one-error. Thus, taking the convention that the ribosome position is quantified via the second P-site nucleotide and then summing over the three nucleotides of the codon (or equivalently staying with the assignment to the first nucleotide of the P-site but then summing over the last nucleotide of the previous codon and the first two nucleotides of the current codon) would seem to be the safer choice. This reviewer realizes that this is something that happens at the very beginning of the analysis and that changing this convention would result in essentially the entire analysis of the manuscript and nearly every figure to have to be redone, but can the authors comment on this somewhat dangerous choice?*

We acknowledge that the choice of the methods for assigning the ribosome position to the first nucleotide of the P-site is not the optimum method. However, given the broad distribution of footprint sizes, the sequence specificity of the MNase cleavage, sequencing errors, and the variability in the P-site offset estimation, we expect the typical error in the ribosome position to be larger than 1 nt. In this case, the effect of the error in the ribosome position on the general statistics will be similar between the two methods.

As an control, we recomputed the ribosome occupancy at the codon level for the two replicates, rep1 and rep2, by summing the ribosome counts from the 3rd nucleotide of the previous codon and the 1st and 2nd nucleotides of the current codon, as suggested by the reviewer. This is equivalent to assigning the ribosome position to the 2nd nucleotide of codons. The metafootprint analysis, normalizing the ribosome counts within CDSs, showed a very similar profile (data not shown). We then computed the average A-site codon elongation rates, which were almost equal to the values obtained with the 1st nucleotide assignment (pearson correlation r = 0.99, Figure 5 in this letter). Given the complexity of the other downstream analyses, we did not recompute the other results using this new assignment method.

Thus, while the choice of the ribosome position assignment method might have a small impact on the landscape of ribosome occupancy, we believe that the main results of our study would not change.

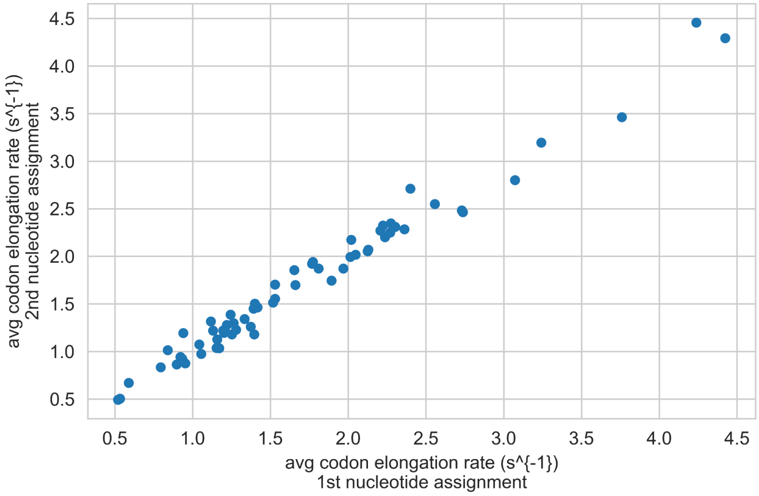

Figure 5: Comparison of codon elongation rates between 1st nucleotide and the 2nd nucleotide assignment methods.

Minor points:
=============
While the manuscript is overall very well written, there are a few small points in which the presentation could be improved:
5) Would it be better to use a logarithmic y axis in figure S2B?

We have modified the scale of the y axis in Appendix Figure S2B.

6) Unless this reviewer missed it, it does not look like figures S4B, S4D, or S4E are referenced in the manuscript.

The Figure S4, now renamed Appendix Figure S3, panels B and D, are referenced in the Discussion section “Determinants of translation initiation rate”, at lines 642, and 672. The panel E is referenced in the Results section “Determinants of translation initiation rates” at line 272.

7) line 473: "(B)" -> "(C)"

Corrected.

*8) Why did the authors choose the average in lines 574 and 576 rather than the sum? It seems that the *total* amount of tRNA for an amino acid should correlate with the synthetase copy number, not their average.*

We agree with the reviewer, and thank him for pointing it out, that the total amount of tRNA isoacceptors, rather than its average, should correlate with the synthetase copy number. We have computed the sum of the relative abundances of tRNAs grouped by amino acid (isoacceptors), averaged between the two replicates, and plotted the correlation with the aaRS copy number in Figure 5F. Although the total abundances were different, the main conclusion from this comparison remained the same: there was no correlation between the two quantities, and Arg still appeared as an outlier.

9) line 1165: "mRNa" -> "mRNA"

Corrected.

10) the legend of Figure S19 does not seem to have much to do with what is actually shown in Figure S19

We have corrected the legend of Figure S19, renamed Appendix Figure S18.

11) Please be mindful of significant digits. Do you really know the conversion coefficient in line 1258 to four digits? Do you think the experimental error on alpha and beta in lines 1359 and 1360 is one in a million? Do you really think your methods determine the total numbers of proteins in a cell to one part per million?

We have corrected the number of significant digits in all numerical values, adequately to the estimated order of magnitude of the associated experimental error.

12) Please be careful with consistent notation. In some places the exponents appear in-line rather than superscripted (e.g., lines 222, 251, and 252). In many places e-notation (1e-4) is used rather than properly typesetting these numbers with proper powers of 10 (and the "e" is sometimes lower case and sometimes upper case), "p" switches between lower and upper case, and the temperature unit sometimes has a degree symbol and sometimes does not and sometimes has a space between the number and the symbol and sometimes does not. The

authors should carefully go through the entire manuscript and supplemental material and make these notations consistent.

We have corrected the notation for exponents, p-values and temperature units.

References

Bartholomäus, A. *et al.* (2016) 'Bacteria differently regulate mRNA abundance to specifically respond to various stresses', *Philosophical transactions. Series A, Mathematical, physical, and engineering sciences*, 374(2063). Available at: https://doi.org/10.1098/rsta.2015.0069.

Bremer, H. and Dennis, P.P. (2008) 'Modulation of Chemical Composition and Other Parameters of the Cell at Different Exponential Growth Rates', *EcoSal Plus*, 3(1). Available at: https://doi.org/10.1128/ecosal.5.2.3.

Cannarozzi, G. *et al.* (2010) 'A role for codon order in translation dynamics', *Cell*, 141(2), pp. 355–367.

Dai, X. *et al.* (2016) 'Reduction of translating ribosomes enables Escherichia coli to maintain elongation rates during slow growth', *Nature Microbiology*, 2(2), pp. 1–9.

Davis, J.H. and Williamson, J.R. (2017) 'Structure and dynamics of bacterial ribosome biogenesis', *Philosophical transactions of the Royal Society of London. Series B, Biological sciences*, 372(1716). Available at: https://doi.org/10.1098/rstb.2016.0181.

Diament, A. and Tuller, T. (2016) 'Estimation of ribosome profiling performance and reproducibility at various levels of resolution', *Biology direct*, 11, p. 24.

Ding, N. *et al.* (2020) 'Programmable cross-ribosome-binding sites to fine-tune the dynamic range of transcription factor-based biosensor', *Nucleic acids research*, 48(18), pp. 10602–10613.

Dingwall, C., Lomonossoff, G.P. and Laskey, R.A. (1981) 'High sequence specificity of micrococcal nuclease', *Nucleic acids research*, 9(12), pp. 2659–2673.

Earnest, T.M. *et al.* (2015) 'Toward a Whole-Cell Model of Ribosome Biogenesis: Kinetic Modeling of SSU Assembly', *Biophysical journal*, 109(6), pp. 1117–1135.

Gerber, S.A. *et al.* (2003) 'Absolute quantification of proteins and phosphoproteins from cell lysates by tandem MS', *Proceedings of the National Academy of Sciences of the United States of America*, 100(12), pp. 6940–6945.

Gorochowski, T.E. *et al.* (2019) 'Absolute quantification of translational regulation and burden using combined sequencing approaches', *Molecular systems biology*, 15(5), p. e8719.

Hwang, J.-Y. and Buskirk, A.R. (2017) 'A ribosome profiling study of mRNA cleavage by the endonuclease RelE', *Nucleic acids research*, 45(1), pp. 327–336.

Lahtvee, P.-J. *et al.* (2017) 'Absolute Quantification of Protein and mRNA Abundances Demonstrate Variability in Gene-Specific Translation Efficiency in Yeast', *Cell systems*, 4(5), pp. 495–504.e5.

Li, H. *et al.* (2012) ‘General LC-MS/MS method approach to quantify therapeutic monoclonal antibodies using a common whole antibody internal standard with application to preclinical studies’, *Analytical chemistry*, 84(3), pp. 1267–1273.

Lyu, X. *et al.* (2021) ‘Codon usage and protein length-dependent feedback from translation elongation regulates translation initiation and elongation speed’, *Nucleic acids research*, 49(16), pp. 9404–9423.

Maier, T. *et al.* (2011) ‘Quantification of mRNA and protein and integration with protein turnover in a bacterium’, *Molecular systems biology*, 7, p. 511.

Mangkalaphiban, K. *et al.* (2021) ‘Transcriptome-wide investigation of stop codon readthrough in Saccharomyces cerevisiae’, *PLoS genetics*, 17(4), p. e1009538.

Mohammad, F., Green, R. and Buskirk, A.R. (2019) ‘A systematically-revised ribosome profiling method for bacteria reveals pauses at single-codon resolution’, *eLife*, 8. Available at: https://doi.org/10.7554/eLife.42591.

Nagalakshmi, U. *et al.* (2008) ‘The transcriptional landscape of the yeast genome defined by RNA sequencing’, *Science*, 320(5881), pp. 1344–1349.

Oeckl, P., Steinacker, P. and Otto, M. (2018) ‘Comparison of Internal Standard Approaches for SRM Analysis of Alpha-Synuclein in Cerebrospinal Fluid’, *Journal of proteome research*, 17(1), pp. 516–523.

O’Reilly, F.J. *et al.* (2020) ‘In-cell architecture of an actively transcribing-translating expressome’, *Science*, 369(6503), pp. 554–557.

Riba, A. *et al.* (2019) ‘Protein synthesis rates and ribosome occupancies reveal determinants of translation elongation rates’, *Proceedings of the National Academy of Sciences of the United States of America*, 116(30), pp. 15023–15032.

Sin, C., Chiarugi, D. and Valleriani, A. (2016) ‘Quantitative assessment of ribosome drop-off in E. coli’, *Nucleic acids research*, 44(6), pp. 2528–2537.

Volkmer, B. and Heinemann, M. (2011) ‘Condition-dependent cell volume and concentration of Escherichia coli to facilitate data conversion for systems biology modeling’, *PloS one*, 6(7), p. e23126.

Xue, L. *et al.* (2022) ‘Visualizing translation dynamics at atomic detail inside a bacterial cell’, *Nature*, 610(7930), pp. 205–211.

Zhu, M. *et al.* (2019) ‘Disruption of transcription-translation coordination in Escherichia coli leads to premature transcriptional termination’, *Nature microbiology*, 4(12), pp. 2347–2356.

4th Jul 2023

Manuscript Number: MSB-2022-11301R
Title: Comprehensive quantitative modeling of translation efficiency in a genome-reduced bacterium

Dear Luis,

Thank you for sending us your revised manuscript. We have now heard back from the reviewers who were asked to evaluate your revised study. Reviewer #4 was asked to assess the responses to the comments of reviewer #2 who was not available this time. As you will see below, both reviewers are satisfied with the modifications made and support publication. They only list a few remaining minor issues, which we would ask you to fix in a final round of minor revisions. We would also ask you to address the editorial issues listed below.

- Our data editors and I have made some minor text edits and formatted the figure legends (see the attached .doc file). Please make all requested text changes using the attached file and *keeping the "track changes" mode* so that we can easily access the edits made.

- Please provide 5 keywords.

- Please note that our editorial policy does not allow "Data not shown" statements.

- Please include callouts for Appendix Figure S2B-C, S3D, S7A-F, S9A-B, S11B, S12C, S14A-D, S15A, S15E, S17A, S23 in the text. The callout for Appendix Fig. S3B should be after S3A, for S4B after S4A, for S12B after S12A, for S13B after S13A and for S13D after S13C.

- Please include page numbers in the Appendix Table of Contents.

- Please provide a "standfirst text" summarizing the study in one or two sentences (approximately 250 characters) and three to four "bullet points" highlighting the main findings.

Please resubmit your revised manuscript online, with a covering letter listing amendments and responses to each point raised by the referees. Please resubmit the paper **within one month** and ideally as soon as possible. If we do not receive the revised manuscript within this time period, the file might be closed and any subsequent resubmission would be treated as a new manuscript. Please use the Manuscript Number (above) in all correspondence.

Click on the link below to submit your revised paper.

https://msb.msubmit.net/cgi-bin/main.plex

As a matter of course, please make sure that you have correctly followed the instructions for authors as given on the submission website.

Thank you for submitting this paper to Molecular Systems Biology.

Kind regards,

Maria

Maria Polychronidou, PhD
Senior Editor
Molecular Systems Biology

If you do choose to resubmit, please click on the link below to submit the revision online before 3rd Aug 2023.

https://msb.msubmit.net/cgi-bin/main.plex

IMPORTANT:
Please note that corresponding authors are required to supply an ORCID ID for their name upon submission of a revised manuscript (EMBO Press signed a joint statement to encourage ORCID adoption).
(https://www.embopress.org/page/journal/17444292/authorguide#editorialprocess)

Currently, our records indicate that the ORCID for your account is 0000-0002-5276-1392.

Please click the link below to modify this ORCID:
Link Not Available

The system will prompt you to fill in your funding and payment information. This will allow Wiley to send you a quote for the article processing charge (APC) in case of acceptance. This quote takes into account any reduction or fee waivers that you may be eligible for. Authors do not need to pay any fees before their manuscript is accepted and transferred to the publisher.

As a matter of course, please make sure that you have correctly followed the instructions for authors as given on the submission website.

*** PLEASE NOTE *** As part of the EMBO Press transparent editorial process initiative (see our Editorial at https://dx.doi.org/10.1038/msb.2010.72 , Molecular Systems Biology will publish online a Review Process File to accompany accepted manuscripts. When preparing your letter of response, please be aware that in the event of acceptance, your cover letter/point-by-point document will be included as part of this File, which will be available to the scientific community. More information about this initiative is available in our Instructions to Authors. If you have any questions about this initiative, please contact the editorial office (msb@embo.org).

Reviewer #3:

Weber et al. took the referees' comments into account quite extensively. The manuscript has become a lot more clear due to these improvements. There remains a tension between the fact that this manuscript represents a treasure trove of data and analyses but with a somewhat lackluster main outcome (namely that the known factors affecting translational regulation do not apply here). It will in the end be an editorial decision if this technical tour de force with a mostly negative outcome warrants publishing this manuscript. It would certainly be a resource for the Mpn community and it does represent an excellent example of how to integrate multiple genome wide experimental approaches and a modeling framework.

A few minor things to change:
1) This referee does not quite understand the relevance of the Neurospora crassa story that is mentioned. Yes, this is a feedback mechanism between translation elongation and translation initiation, but it is a global one. For what the authors find, a local (gene by gene) feedback mechanism would be required, which seems a lot more complicated to imagine. The manuscript should be explicit about this important difference between what is observed in Neurospora crassa and what would be needed here.
2) This referee appreciates that the authors have changed the number of digits in most quantities that they report. However, the total numbers of proteins in lines 1273-1274 are still reported to a six digit precision, which seems extensive.
3) "MPN" in line 623 should be "Mpn"
4) A space is needed before the "at" in line 1315

Reviewer #4:

Review of "Comprehensive quantitative modelling of translational efficiency in a genome-reduced bacterium"

Webber et al. present an excellent and well thought-out analysis of translational efficiency using a combination of high throughput approaches including RNA-seq, proteomics, ribosome profiling, tRNA quantification and mass-spectrometry in the genome reduced bacterium, Mycoplasma pneumoniae. The work presented is well within the scope of Molecular Systems Biology, was an interesting read, and makes use of good supporting literature with useful comparisons made to other model systems where appropriate. The manuscript is well written, and the results of their efforts have culminated in findings which point towards additional unknown global mechanisms of translation control, as well as demonstrating the lack of contribution of several factors at the stages of initiation and elongation which were initially thought to be critical in other systems. For example, revealing the that presence of the Shine-Dalgarno (SD) motif has little to no influence on translational efficiency within M. pneumoniae, and that the low frequency of SD utilisation could be reflective of the differences in growth rates between various related species.

In summary, this work will not only be beneficial to individuals with interest in the field of translational control alone, but there is clear scope of this work to provide a useful blue-print which could prove beneficial in understanding translational efficiencies in engineered synthetic minimal genomes designed using either bottom-up or top-down approaches, and also potentially fine-tuning this process in these systems for their optimisation.

Whilst major comments have been addressed rather well in the revised manuscript, there are additional minor comments I would like to propose for manuscript improvement that should be addressed.

1) Line 623: "...or, as it happens in MPN, higher..."
- For consistency with the rest of the text, MPN should be written as Mpn, and elsewhere in the text where appropriate. This appears again on line 1078 "The transcriptional landscape in MPN....". Finally, "Mpn" should be italicised (Mpn) even when written as the shorthand for Mycoplasma pneumoniae.

2) Line 89- 92: "By integrating the absolute quantifications of mRNA abundances, protein abundances, and protein half-lives with ribosome density, we derived initiation and elongation rates for 475 genes, 73 with high precision. By integrating the absolute quantifications of mRNA abundances, protein abundances, and protein half-lives with ribosome density, we derived initiation and elongation rates for 475 genes, 73 with high precision."
- Although it is mentioned earlier in the text that mRNA-levels and their half-lives for Mpn were quantified in a previous study by Junier et al, 2016, it should be mentioned for clarity that this was also integrated to derive initiation and elongation rates as it is missing in the text here.

3) Lines 265-267: "We did not find any significant correlation (Pearson coefficient $|r| < 0.2$, $p > 0.01$) between the absolute translation efficiency and nucleotide composition upstream of the start codon (Appendix Figure S4A, Appendix Figure S5)."
- Please mention the number of/window of nucleotides upstream (15 nt) used in the analysis in the main body of the text.

4) Lines 275-283: "Moreover, the biophysical interactions between positively charged residues and the ribosome exit tunnel have been suggested to favor elongation of the nascent peptide at the N-terminal (Dao Duc & Song, 2018).We found that the fraction of positively charged amino acids (Lys, Arg, His) in the first 35 residues at the N-terminal did not correlate with kTEabs ($r = -0.04$, $N = 474$, $p = 0.3$), however the fraction of hydrophobic residues (Ala, Ile, Leu, Met, Val, Phe, Trp, Tyr) showed a weak negative correlation ($r = -0.16$, $N = 474$, $p = 3.5 \times 10-4$) and the fraction of negatively charged residues (Glu, Asp) a weak positive correlation ($r = 0.21$, $N = 474$, $p = 4.4 \times 10-6$) with the translation efficiency (Appendix Figure S7). This suggests that hydrophobic residues at the N-terminal could slow down the rate of translation initiation, while negative residues could have the opposite effect. Finally no correlation was found between kTEabs and RNA degradation rate (Appendix Figure S4C) ($r = 0.08$, $N = 462$, $p = 0.08$)."
- Whilst this section is well written, the text first makes reference to the biophysical interactions that occur that favour the elongation of the nascent peptide. Therefore, would this text therefore not be better suited for the subsequent subsection titled "Elongation rates"? As a side note (in future work), it would be excellent to see experimentally how modifications to these residues within the first 35 amino acids could alter translation elongation from a biological design perspective. This could prove relevant for other genome reduced bacteria with industrial relevance.

- Whilst on the topic of biophysical interactions which may influence elongation the peptide, there is scarce mention of this in the discussion section. Could a mechanistic basis for why positively charged amino acids at the N-terminal favour elongation (based on Dao Duc & Song, 2018) be provided, and why may this observation differed in M. pneumoniae, or why hydrophobicity may slow down elongation? Is there sufficient literature to support this explanation?

Point by point answer, second revision, manuscript "Comprehensive quantitative modeling of translation efficiency in a genome-reduced bacterium" MSB-2022-11301R

Reviewer #3 comments

Weber et al. took the referees' comments into account quite extensively. The manuscript has become a lot more clear due to these improvements. There remains a tension between the fact that this manuscript represents a treasure trove of data and analyses but with a somewhat lackluster main outcome (namely that the known factors affecting translational regulation do not apply here). It will in the end be an editorial decision if this technical tour de force with a mostly negative outcome warrants publishing this manuscript. It would certainly be a resource for the Mpn community and it does represent an excellent example of how to integrate multiple genome wide experimental approaches and a modeling framework.

A few minor things to change:
1) This referee does not quite understand the relevance of the Neurospora crassa story that is mentioned. Yes, this is a feedback mechanism between translation elongation and translation initiation, but it is a global one. For what the authors find, a local (gene by gene) feedback mechanism would be required, which seems a lot more complicated to imagine. The manuscript should be explicit about this important difference between what is observed in Neurospora crassa and what would be needed here.

> We agree with the reviewer that in *Neurospora crassa*, the described feedback mechanism, mediated by the phosphorylation of eIF2α, is global. However, as the authors of the mentioned study showed, in physiological conditions, eIF2α phosphorylation regulates translation initiation and elongation rates in a specific manner. The loss of eIF2α phosphorylation preferentially increased the abundances of large proteins encoded by mRNAs with non-optimal codon usage, which suggests that a high local concentration of phosphorylated eIF2α specifically inhibits translation initiation of long CDS mRNAs with non-optimal codon usage. We speculate that a similar global feedback mechanism could exist in *Mpn* and could inhibit preferentially the initiation rates of genes with slow elongation rates or possessing specific sequence features. In any way, the existence of such a mechanism, whether global or local, remains speculative. We have clarified the manuscript in this regard (lines 715-716).

2) This referee appreciates that the authors have changed the number of digits in most quantities that they report. However, the total numbers of proteins in lines 1273-1274 are still reported to a six digit precision, which seems extensive.

We have corrected the number of significant digits for the total number of proteins.

3) "MPN" in line 623 should be "Mpn"

Following recommendation by reviewer #4, we have corrected the format of the abbreviated form to italicized *Mpn*, everywhere in the main text and in the appendix figure legends.

4) A space is needed before the "at" in line 1315

We could not find the mentioned lacking space in the manuscript.

Reviewer #4 comments

Review of "Comprehensive quantitative modelling of translational efficiency in a genome-reduced bacterium"

Webber et al. present an excellent and well thought-out analysis of translational efficiency using a combination of high throughput approaches including RNA-seq, proteomics, ribosome profiling, tRNA quantification and mass-spectrometry in the genome reduced bacterium, Mycoplasma pneumoniae. The work presented is well within the scope of Molecular Systems Biology, was an interesting read, and makes use of good supporting literature with useful comparisons made to other model systems where appropriate. The manuscript is well written, and the results of their efforts have culminated in findings which point towards additional unknown global mechanisms of translation control, as well as demonstrating the lack of contribution of several factors at the stages of initiation and elongation which were initially thought to be critical in other systems. For example, revealing the that presence of the Shine-Dalgarno (SD) motif has little to no influence on translational efficiency within M. pneumoniae, and that the low frequency of SD utilisation could be reflective of the differences in growth rates between various related species.

In summary, this work will not only be beneficial to individuals with interest in the field of translational control alone, but there is clear scope of this work to provide a useful blue-print which could prove beneficial in understanding translational efficiencies in engineered synthetic minimal genomes designed using either bottom-up or top-down approaches, and also potentially fine-tuning this process in these systems for their optimisation.

Whilst major comments have been addressed rather well in the revised manuscript, there are additional minor comments I would like to propose for manuscript improvement that should be addressed.

1) Line 623: "...or, as it happens in MPN, higher..."
- For consistency with the rest of the text, MPN should be written as Mpn, and elsewhere in the text where appropriate. This appears again on line 1078 "The transcriptional landscape in

MPN....". Finally, "Mpn" should be italicised (Mpn) even when written as the shorthand for Mycoplasma pneumoniae.

We have corrected the format of the abbreviated form to *Mpn*, everywhere in the main text and in the appendix figure legends.

2) Line 89- 92: "By integrating the absolute quantifications of mRNA abundances, protein abundances, and protein half-lives with ribosome density, we derived initiation and elongation rates for 475 genes, 73 with high precision. By integrating the absolute quantifications of mRNA abundances, protein abundances, and protein half-lives with ribosome density, we derived initiation and elongation rates for 475 genes, 73 with high precision."
- Although it is mentioned earlier in the text that mRNA-levels and their half-lives for Mpn were quantified in a previous study by Junier et al, 2016, it should be mentioned for clarity that this was also integrated to derive initiation and elongation rates as it is missing in the text here.

In our simple modeling approach, the mRNA half-lives were not used to derive the translation efficiency, k_{TE} = $lambda_P$ * P / mRNA, since the mRNA levels were assumed to be at equilibrium. mRNA half-lives were only used in our correlation analyses.

3) Lines 265-267: "We did not find any significant correlation (Pearson coefficient $|r| < 0.2$, $p > 0.01$) between the absolute translation efficiency and nucleotide composition upstream of the start codon (Appendix Figure S4A, Appendix Figure S5)."
- Please mention the number of/window of nucleotides upstream (15 nt) used in the analysis in the main body of the text.

Corrected.

4) Lines 275-283: "Moreover, the biophysical interactions between positively charged residues and the ribosome exit tunnel have been suggested to favor elongation of the nascent peptide at the N-terminal (Dao Duc & Song, 2018).We found that the fraction of positively charged amino acids (Lys, Arg, His) in the first 35 residues at the N-terminal did not correlate with kTEabs (r = -0.04, N = 474, p = 0.3), however the fraction of hydrophobic residues (Ala, Ile, Leu, Met, Val, Phe, Trp, Tyr) showed a weak negative correlation (r = -0.16, N = 474, p = 3.5 × 10-4) and the fraction of negatively charged residues (Glu, Asp) a weak positive correlation (r = 0.21, N = 474, p = 4.4 × 10-6) with the translation efficiency (Appendix Figure S7). This suggests that hydrophobic residues at the N-terminal could slow down the rate of translation initiation, while negative residues could have the opposite effect. Finally no correlation was found between kTEabs and RNA degradation rate (Appendix Figure S4C) (r = 0.08, N = 462, p = 0.08)."
- Whilst this section is well written, the text first makes reference to the biophysical interactions that occur that favour the elongation of the nascent peptide. Therefore, would this text therefore not be better suited for the subsequent subsection titled "Elongation rates"? As a side note (in future work), it would be excellent to see experimentally how modifications to these residues within the first 35 amino acids could alter translation elongation from a biological design

perspective. This could prove relevant for other genome reduced bacteria with industrial relevance.

Previous studies have shown that the first ~30 codons of the coding sequence can strongly influence the initial stages of translation elongation (Dao Duc & Song, 2018; Verma *et al*, 2019), and that those features can have an impact on the gene-level translation efficiency as well.
In our modeling approach, we considered that the elongation rate in the first 30 codons can be included in an effective initiation rate. Strong determinants in this 5' region would control the rate of early elongation which could become the limiting step in translation, and thus control the gene-level translation efficiency (e.g. 5' ramp hypothesis). If this is the case, such features in the 5' region would correlate with the absolute translation efficiency at the gene-level, and have a similar effect at the gene-level as features controlling translation initiation.
When computing the elongation rates, we discarded the first 30 codons and only considered downstream positions, in order to avoid the influence from 5' features and possible experimental artifacts.
We have clarified the manuscript in this regard (lines 270-272).

- Whilst on the topic of biophysical interactions which may influence elongation the peptide, there is scarce mention of this in the discussion section. Could a mechanistic basis for why positively charged amino acids at the N-terminal favour elongation (based on Dao Duc & Song, 2018) be provided, and why may this observation differed in M. pneumoniae, or why hydrophobicity may slow down elongation? Is there sufficient literature to support this explanation?

The effect of the biophysical interactions between the polypeptide residues and the ribosome exit tunnel remain poorly understood and difficult to predict. Moreover, no crystallographic structure of the ribosome in Mpn is available.
We have included a full paragraph in the discussion about the effect of positively and negatively charged residues on elongation rates (lines 740-753), comparing our results to several studies in yeast.

References

Dao Duc K & Song YS (2018) The impact of ribosomal interference, codon usage, and exit tunnel interactions on translation elongation rate variation. *PLoS Genet* 14: e1007166

Verma M, Choi J, Cottrell KA, Lavagnino Z, Thomas EN, Pavlovic-Djuranovic S, Szczesny P, Piston DW, Zaher HS, Puglisi JD, *et al* (2019) A short translational ramp determines the efficiency of protein synthesis. *Nat Commun* 10: 5774

24th Jul 2023

Manuscript number: MSB-2022-11301RR
Title: Comprehensive quantitative modeling of translation efficiency in a genome-reduced bacterium

Dear Luis,

Thank you again for sending us your revised manuscript. We are now satisfied with the modifications made and I am pleased to inform you that your paper has been accepted for publication.

*** PLEASE NOTE *** As part of the EMBO Publications transparent editorial process initiative (see our Editorial at https://dx.doi.org/10.103/msb.2010.72), Molecular Systems Biology publishes online a Review Process File with each accepted manuscripts. This file will be published in conjunction with your paper and will include the anonymous referee reports, your point- by-point response and all pertinent correspondence relating to the manuscript. If you do NOT want this File to be published, please inform the editorial office at msb@embo.org within 14 days upon receipt of the present letter.

LICENSE AND PAYMENT:
All articles published in Molecular Systems Biology are fully open access: immediately and freely available to read, download and share.

Molecular Systems Biology charges an article processing charge (APC) to cover the publication costs. You, as the corresponding author for this manuscript, should have already received a quote with the article processing fee separately. Please let us know in case this quote has not been received.

Once your article is at Wiley for editorial production, you will receive an email from Wiley's Author Services system, which will ask you to log in and will present you with the publication license form for completion. Within the same system the publication fee can be paid by credit card, an invoice or pro forma can be requested.

Payment of the publication charge and the signed Open Access Agreement form must be received before the article can be published online.

Upon acceptance it is mandatory for you to return the completed payment form. Failure to send back the form may result in a delay in the publication of your paper.

Molecular Systems Biology articles are published under the Creative Commons licence CC BY, which facilitates the sharing of scientific information by reducing legal barriers, while mandating attribution of the source in accordance to standard scholarly practice.

Proofs will be forwarded to you within the next 2-3 weeks.

Thank you very much for submitting your work to Molecular Systems Biology.

Kind regards,

Maria

Maria Polychronidou, PhD
Senior Editor
Molecular Systems Biology

EMBO Press Author Checklist

Corresponding Author Name: Luis Serrano	
Journal Submitted to: Molecular Systems Biology	
Manuscript Number: #MSB-2022-11301	

USEFUL LINKS FOR COMPLETING THIS FORM
The EMBO Journal - Author Guidelines
EMBO Reports - Author Guidelines
Molecular Systems Biology - Author Guidelines
EMBO Molecular Medicine - Author Guidelines

Reporting Checklist for Life Science Articles (updated January 2022)

This checklist is adapted from Materials Design Analysis Reporting (MDAR) Checklist for Authors. MDAR establishes a minimum set of requirements in transparent reporting in the life sciences (see Statement of Task: 10.31222/osf.io/9sm4x). Please follow the journal's guidelines in preparing your manuscript.

Please note that a copy of this checklist will be published alongside your article.

Abridged guidelines for figures

1. Data

The data shown in figures should satisfy the following conditions:

- the data were obtained and processed according to the field's best practice and are presented to reflect the results of the experiments in an accurate and unbiased manner.
- ideally, figure panels should include only measurements that are directly comparable to each other and obtained with the same assay.
- plots include clearly labeled error bars for independent experiments and sample sizes. Unless justified, error bars should not be shown for technical replicates.
- if n<5, the individual data points from each experiment should be plotted. Any statistical test employed should be justified.
- Source Data should be included to report the data underlying figures according to the guidelines set out in the authorship guidelines on Data Presentation.

2. Captions

Each figure caption should contain the following information, for each panel where they are relevant:

- a specification of the experimental system investigated (eg cell line, species name).
- the assay(s) and method(s) used to carry out the reported observations and measurements.
- an explicit mention of the biological and chemical entity(ies) that are being measured.
- an explicit mention of the biological and chemical entity(ies) that are altered/varied/perturbed in a controlled manner.
- the exact sample size (n) for each experimental group/condition, given as a number, not a range;
- a description of the sample collection allowing the reader to understand whether the samples represent technical or biological replicates (including how many animals, litters, cultures, etc.).
- a statement of how many times the experiment shown was independently replicated in the laboratory.
- definitions of statistical methods and measures:
  - common tests, such as t-test (please specify whether paired vs. unpaired), simple χ^2 tests, Wilcoxon and Mann-Whitney tests, can be unambiguously identified by name only, but more complex techniques should be described in the methods section;
  - are tests one-sided or two-sided?
  - are there adjustments for multiple comparisons?
  - exact statistical test results, e.g., P values = x but not P values < x;
  - definition of 'center values' as median or average;
  - definition of error bars as s.d. or s.e.m.

Please complete ALL of the questions below.
Select "Not Applicable" only when the requested information is not relevant for your study.

Materials

Newly Created Materials	Information included in the manuscript?	In which section is the information available? (Reagents and Tools Table, Materials and Methods, Figures, Data Availability Section)
New materials and reagents need to be available; do any restrictions apply?	Not Applicable	

Antibodies	Information included in the manuscript?	In which section is the information available? (Reagents and Tools Table, Materials and Methods, Figures, Data Availability Section)
For **antibodies** provide the following information:<br>- Commercial antibodies: RRID (if possible) or supplier name, catalogue number and or/clone number<br>- Non-commercial: RRID or citation	Not Applicable	

DNA and RNA sequences	Information included in the manuscript?	In which section is the information available? (Reagents and Tools Table, Materials and Methods, Figures, Data Availability Section)
**Short novel DNA or RNA including primers, probes**: provide the sequences.	Not Applicable	

Cell materials	Information included in the manuscript?	In which section is the information available? (Reagents and Tools Table, Materials and Methods, Figures, Data Availability Section)
**Cell lines:** Provide species information, strain. Provide accession number in repository **OR** supplier name, catalog number, clone number, and/**OR** RRID.	Not Applicable	
**Primary cultures:** Provide species, strain, sex of origin, genetic modification status.	Yes	Materials and Methods
Report if the cell lines were recently **authenticated** (e.g., by STR profiling) and tested for mycoplasma contamination.	Not Applicable	

Experimental animals	Information included in the manuscript?	In which section is the information available? (Reagents and Tools Table, Materials and Methods, Figures, Data Availability Section)
**Laboratory animals or Model organisms:** Provide species, strain, sex, age, genetic modification status. Provide accession number in repository **OR** supplier name, catalog number, clone number, **OR** RRID.	Not Applicable	
**Animal observed in or captured from the field:** Provide species, sex, and age where possible.	Not Applicable	
Please detail **housing and husbandry conditions**.	Not Applicable	

Plants and microbes	Information included in the manuscript?	In which section is the information available? (Reagents and Tools Table, Materials and Methods, Figures, Data Availability Section)
**Plants:** provide species and strain, ecotype and cultivar where relevant, unique accession number if available, and source (including location for collected wild specimens).	Not Applicable	
**Microbes:** provide species and strain, unique accession number if available, and source.	Yes	Materials and Methods

Human research participants	Information included in the manuscript?	In which section is the information available? (Reagents and Tools Table, Materials and Methods, Figures, Data Availability Section)
If collected and within the bounds of privacy constraints report on age, sex and gender or ethnicity for all study participants.	Not Applicable	

Core facilities	Information included in the manuscript?	In which section is the information available? (Reagents and Tools Table, Materials and Methods, Figures, Data Availability Section)
If your work benefited from core facilities, was their service mentioned in the acknowledgments section?	Yes	

Design

Study protocol	Information included in the manuscript?	In which section is the information available? (Reagents and Tools Table, Materials and Methods, Figures, Data Availability Section)
If study protocol has been **pre-registered, provide DOI in the manuscript**. For clinical trials, provide the trial registration number **OR** cite DOI.	Not Applicable	
Report the **clinical trial registration number** (at ClinicalTrials.gov or equivalent), where applicable.	Not Applicable	

Laboratory protocol	Information included in the manuscript?	In which section is the information available? (Reagents and Tools Table, Materials and Methods, Figures, Data Availability Section)
Provide DOI OR other citation details if **external detailed step-by-step protocols** are available.	Yes	Materials and Methods

Experimental study design and statistics	Information included in the manuscript?	In which section is the information available? (Reagents and Tools Table, Materials and Methods, Figures, Data Availability Section)
Include a statement about **sample size** estimate even if no statistical methods were used.	Yes	Materials and Methods
Were any steps taken to minimize the effects of subjective bias when allocating animals/samples to treatment (e.g. **randomization procedure**)? If yes, have they been described?	Not Applicable	
Include a statement about **blinding** even if no blinding was done.	Not Applicable	
Describe **inclusion/exclusion criteria** if samples or animals were excluded from the analysis. Were the criteria pre-established?<br><br>If sample or data points were omitted from analysis, report if this was due to attrition or intentional exclusion and provide justification.	Not Applicable	
For every figure, are **statistical tests** justified as appropriate? Do the data meet the assumptions of the tests (e.g., normal distribution)? Describe any methods used to assess it. Is there an estimate of variation within each group of data? Is the variance similar between the groups that are being statistically compared?	Yes	

Sample definition and in-laboratory replication	Information included in the manuscript?	In which section is the information available? (Reagents and Tools Table, Materials and Methods, Figures, Data Availability Section)
In the figure legends: state number of times the experiment was **replicated** in laboratory.	Yes	
In the figure legends: define whether data describe **technical or biological replicates**.	Yes	

Ethics

Ethics	Information included in the manuscript?	In which section is the information available? (Reagents and Tools Table, Materials and Methods, Figures, Data Availability Section)
Studies involving **human participants**: State details of **authority granting ethics approval** (IRB or equivalent committee(s), provide reference number for approval.	Not Applicable	
Studies involving **human participants**: Include a statement confirming that **informed consent** was obtained from all subjects and that the experiments conformed to the principles set out in the WMA Declaration of Helsinki and the Department of Health and Human Services Belmont Report.	Not Applicable	
Studies involving **human participants:** For publication of **patient photos**, include a statement confirming that consent to publish was obtained.	Not Applicable	
Studies involving experimental **animals**: State details of **authority granting ethics approval** (IRB or equivalent committee(s), provide reference number for approval. Include a statement of compliance with ethical regulations.	Not Applicable	
Studies involving **specimen and field samples:** State if relevant **permits** obtained, provide details of authority approving study; if none were required, explain why.	Not Applicable	

Dual Use Research of Concern (DURC)	Information included in the manuscript?	In which section is the information available? (Reagents and Tools Table, Materials and Methods, Figures, Data Availability Section)
Could your study fall under dual use research restrictions? Please check biosecurity documents and list of select agents and toxins (CDC): https://www.selectagents.gov/sat/list.htm	Not Applicable	
If you used a select agent, is the security level of the lab appropriate and reported in the manuscript?	Not Applicable	
If a study is subject to dual use research of concern regulations, is the name of the **authority granting approval and reference number** for the regulatory approval provided in the manuscript?	Not Applicable	

Reporting

The MDAR framework recommends adoption of discipline-specific guidelines, established and endorsed through community initiatives. Journals have their own policy about requiring specific guidelines and recommendations to complement MDAR.

Adherence to community standards	Information included in the manuscript?	In which section is the information available? (Reagents and Tools Table, Materials and Methods, Figures, Data Availability Section)
State if relevant guidelines or checklists (e.g., **ICMJE, MIBBI, ARRIVE, PRISMA**) have been followed or provided.	Not Applicable	
For **tumor marker prognostic studies,** we recommend that you follow the **REMARK** reporting guidelines (see link list at top right). See author guidelines, under 'Reporting Guidelines'. Please confirm you have followed these guidelines.	Not Applicable	
For **phase II and III randomized controlled trials**, please refer to the **CONSORT** flow diagram (see link list at top right) and submit the CONSORT checklist (see link list at top right) with your submission. See author guidelines, under 'Reporting Guidelines'. Please confirm you have submitted this list.	Not Applicable	

Data Availability

Data availability	Information included in the manuscript?	In which section is the information available? (Reagents and Tools Table, Materials and Methods, Figures, Data Availability Section)
Have **primary datasets** been deposited according to the journal's guidelines (see 'Data Deposition' section) and the respective accession numbers provided in the Data Availability Section?	Yes	Data Availability
Were **human clinical and genomic datasets** deposited in a public access-controlled repository in accordance to ethical obligations to the patients and to the applicable consent agreement?	Not Applicable	
Are **computational models** that are central and integral to a study available without restrictions in a machine-readable form? Were the relevant accession numbers or links provided?	Not Applicable	
If publicly available data were reused, provide the respective **data citations in the reference list**.	Yes	Main text and Materials and Methods
